# Multiple imputation for missing values in ordinal variables from cancer registry data when performing Cox proportional hazards regression

**DOI:** 10.1186/s12874-026-02790-8

**Published:** 2026-02-06

**Authors:** Anika Kästner, Wolfgang Hoffmann, Johannes Hüsing, Andreas Stang, Anika Hüsing

**Affiliations:** 1 Institute for Community Medicine, Section Epidemiology of Health Care and Community Health, University Medicine Greifswald, Ellernholzstraße 1-2, Greifswald, 17487 Germany; 2 Institute of Medical Informatics, Biometry and Epidemiology, University Hospital Essen, Hufelandstraße 55, 45147 Essen, Germany; 3 Cancer Registry North Rhine-Westphalia, Gesundheitscampus 10, Bochum, 44801 Germany

**Keywords:** Imputation, Registries, Missing data, Survival analysis, Neoplasms

## Abstract

**Background:**

Scientists working with cancer registry data are often confronted with large proportions of missing values in ordinal variables, such as tumor stage, grading or the general health status (ECOG-PS scored 0 to 5). Despite the long-standing issue, research on handling missing ordinal cancer registry data remains sparse.

**Methods:**

A simulation study was conducted using complete lung cancer cases (2019–2022) from the North Rhine-Westphalia Cancer Registry. Missing values in ECOG-PS were generated with varying missingness mechanisms (MCAR, MAR, MNAR), missingness proportions (10% to 50%) and sample sizes (*N* = 500, *N* = 1,000, *N* = 5,000). The data were then replaced using MICE with ordinal logistic regression (POLR), multinomial regression (POLYREG), predictive mean matching (PMM), random forests (RF), and the joint model (JM). The performance parameters bias, MSE, width of the 95%CI and coverage were assessed.

**Results:**

Severe bias, high MSE, wide 95%CI, and poor coverage were found in scenarios with sample sizes of *N* = 500 and 1,000 and 30% or more missing data with low prevalence of ECOG-PS = 4. MICE with POLYREG maintained low bias across all scenarios with *N* = 5,000, while MICE with RF and PMM performed well with up to 30%-50% missing data. MICE with POLR and the JM yielded low bias with up to 10%-20% missing data. Compared to complete case analysis, MI did not offer a systematic advantage in terms of bias or MSE compared to the MI methods evaluated.

**Conclusion:**

Sample size and ordinal category distribution impact missing data handling in registry studies. Severe bias might be introduced when sample sizes are smaller and prevalence of categories is low, indicating finite-sample effects rather than systematic bias of the imputation methods. Among the MI methods applied, MICE with POLYREG performed best, however, further research is needed for time-to-event analyses and multivariate missingness patterns.

**Supplementary Information:**

The online version contains supplementary material available at 10.1186/s12874-026-02790-8.

## Background

Working with cancer registry data, epidemiologists are often confronted with substantial proportions of missing information [1, 2]. Multiple imputation (MI) is widely recommended for dealing with missing values, as it can improve statistical efficiency and inference compared to performing complete case analysis (CCA) under appropriate missingness assumptions [3]. Specifically, it has been previously reported that when missingness in covariate data is associated with the survival outcome, CCA may lead to biased regression coefficient estimates [4, 5]. Despite all advantages that MI offers, there still seems to be a considerable gap between theory and practical application of this method. A review of observational time-to-event studies in oncology showed that although missing data were present in most studies, CCA was the most commonly applied method [6]. Only about a quarter (22%) applied MI to deal with missing values.

The evaluation of the performance of MI for missing cancer registry data is particularly important due to the largely comparable information collected in cancer registries in most countries. In German cancer registries, missing or unknown data on tumor stage or the general health status (Eastern Cooperative Oncology Group performance status, ECOG-PS) were reported in 15% to 25% of cancer registrations [7]. These variables are potential baseline confounders, as they directly impact both the choice of treatment and overall survival [8]. Moreover, such ordinal variables represent one of the most challenging variable types to impute [9]. White et al. recommended using multinomial logistic regression (POLYREG) or the proportional odds model (POLR) as imputation models for ordinal variables [10]. However, in real-world epidemiological data, the proportional odds assumption is often violated, and previous studies comparing methods for imputing missing tumor stage values have found this model to be unsuitable [11]. Other proposed methods are MICE with predictive mean matching (PMM), random forests (RF) [12], and the joint model (JM) with latent normal variables [13].

No study has compared all of these proposed methods and thus it remains unclear which method is best suited for imputing ordinal values of cancer registry data under which conditions [14]. Therefore, the aim of this study was to compare the performance of different MI methods for imputing missing ordinal ECOG-PS values in real-world cancer registry data when performing Cox proportional hazards survival analysis.

## Methods

The study was approved by the ethics committee of the University Medicine Greifswald (BB 090/24). The study was conducted in accordance with the principles of the Declaration of Helsinki. The legal basis for the use of cancer registry data for research purposes was the respective cancer registration act of North Rhine-Westphalia. No individual informed consent was required, as data processing and secondary use of pseudonymized data for scientific research are explicitly permitted. The data were provided after approval of a data access request in accordance with the applicable use and access regulations.

### Cancer registry data

The Cancer Registry of North Rhine-Westphalia provided a total of *N* = 60,444 primary malignant lung cancer cases (ICD-10: C34.-) diagnosed between January 1, 2019, and December 31, 2022.

The ECOG-PS is an ordinal scale with absolute values ranging from 0 to 5, representing the patient’s general health condition as shown in Table 1, which also relates the ECOG-PS definitions to the corresponding Karnofsky criteria [15]. Since grade 5 (= dead) is not expected at initial lung cancer diagnosis, which was confirmed by the data, solely grades 0 to 4 were considered.

**Table 1:** **–** Definition of the grades of the Eastern cooperative oncology group performance status (ECOG-PS) [15]

Grade	Definition of the Eastern Cooperative Oncology Group performance status grade	Karnofsky criteria
0	Fully active, able to carry on all pre-disease performance without restriction	100 – Normal, no complaints; no evidence of disease 90 – Able to carry on normal activity; minor signs or symptoms of disease
1	Restricted in physically strenuous activity but ambulatory and able to carry out work of a light or sedentary nature, e.g., light house work, office work	80 – Normal activity with effort, some signs or symptoms of disease 70 – Cares for self but unable to carry on normal activity or to do active work
2	Ambulatory and capable of all self-care but unable to carry out any work activities. Up and about more than 50% of waking hours	60 – Requires occasional assistance but is able to care for most of personal needs 50 – Requires considerable assistance and frequent medical care
3	Capable of only limited selfcare, confined to bed or chair more than 50% of waking hours	40 – Disabled; requires special care and assistance 30 – Severely disabled; hospitalization is indicated although death not imminent
4	Completely disabled. Cannot carry on any selfcare. Totally confined to bed or chair	20 – Very ill; hospitalization and active supportive care necessary 10 – Moribund
5	Dead	0 – Dead

### Simulation design and data-generating mechanisms

**Fig. 1:**
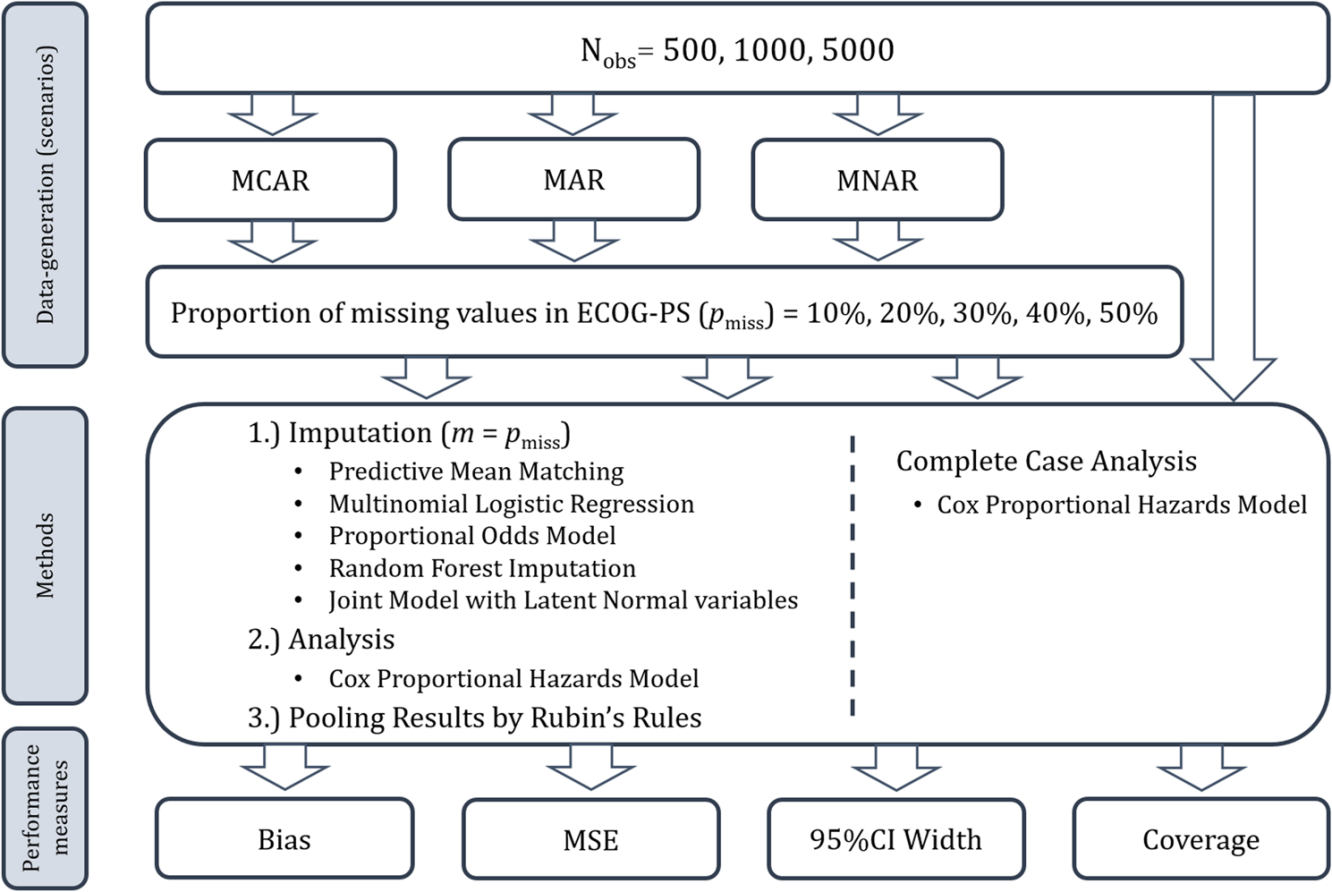
Overview of the simulation design

The methods described in the following are based on the principles proposed by Oberman and Vink [16] for evaluating imputation techniques and by Morris et al. for evaluating statistical methods using simulation studies [17]. In this simulation study, the following factors varied: the sample size (*N* = 500, *N* = 1,000 and *N* = 5,000), the missingness proportions of ECOG-PS (10%, 20%, 30%, 40%, and 50%) and the missingness mechanisms (MCAR: Missing Completely At Random, MAR: Missing At Random, MNAR: Missing Not At Random), resulting in 45 distinct missingness scenarios. The complete cancer registry dataset served as basis for drawing random samples without replacement. An overview of the simulation design is given in Fig. 1.

The missing ECOG-PS values were simulated using the ‘ampute’ function of the R package ‘mice’, which is based on weighted sum scores [18].

Data are MCAR if the probability of a value being missing is completely unrelated to the observed and unobserved data [19]. In other words, the probability of a value being missing is the same for all cases. To generate MCAR ECOG-PS values, all weights were set to zero so that no variable influences the occurrence of missing values, ensuring they are generated entirely at random [18].

Data are MAR if the probability of a value being missing is dependent on the observed data and independent on the unobserved data [19]. To define missing values for ECOG-PS under the MAR assumption, weights had to be chosen for variables that quantify the influence. To mimic a realistic missingness pattern, a logistic regression was conducted based on all lung cancer cases to examine which variables influence the missingness of ECOG-PS values. The beta coefficients of the statistically significant variables, including their positive or negative signs, were used as weights (see additional file 1, Supplementary Table S1). As missingness depended on a survival-related quantity (the Nelson–Aalen estimate), missing ECOG-PS values were simulated to dependent on the observed survival outcome (outcome-dependent MAR).

Data are MNAR if the probability of a value being missing depends on the missing value itself, and this dependency remains even given the observed data [19]. To impute missing values under the MNAR assumption, a weight of 1 was assigned to the ECOG-PS variable, while all other variables received a weight of zero [18].

### Estimand

The log hazard ratios (log(HR)) of the ordinal variable ECOG-PS for overall survival served as estimand of this simulation study.

### Multiple imputation methods

The following MI methods were applied: MICE with PMM, POLR, POLYREG, and RF and the JM as imputation methods. Each MI method was applied to every simulated incomplete dataset (full factorial design) [16]. Additionally, CCA was performed to assess the effect of the application of MI methods compared to the deletion of cases with missing ECOG-PS values [20].

Briefly, PMM is a donor-based imputation method that replaces missing values with observed values from cases with similar predicted means [19]. POLR and POLYREG are regression-based imputation approaches for ordinal and nominal variables, respectively [10, 19]. RF imputation is a nonparametric, tree-based method that can accommodate nonlinear relationships and interactions [21]. JM refers to multivariate imputation based on a common multivariate normal distribution assumption for all variables [19]. In CCA, cases with missing values in any variable included in the analysis model are excluded.

To ensure compatibility between the imputation and analysis model, both models included the same variables without any interactions [10]. The Nelson-Aalen estimator of the cumulative hazard and the event indicator were included in the imputation model instead of the survival time, as recommended by White and Royston [22]. The imputation model contained the following variables: age in years, sex (male/female), Nelson-Aalen estimator of the cumulative hazard, survival status (alive/dead), C34.0 (yes/no), C34.1 (yes/no), C34.3 (yes/no), adenocarcinoma (yes/no), squamous cell carcinoma (yes/no), small cell carcinoma (yes/no), first-line radiotherapy (yes vs. no therapy reported), intention of first-line therapy (palliative intention vs. curative intention vs. no therapy reported), residual status after surgery (surgery, R1 vs. surgery, R0 vs. no surgery reported), first-line systemic therapy (chemotherapy vs. immuno-chemotherapy vs. mono-immunotherapy vs. targeted therapy vs. no first-line systemic therapy reported), diagnosis year (2019/2020/2021/2022), ECOG-PS (0/1/2/3/4), grading (G1/G2/G3/G4), and tumor stage (IA1/IA2/IA3/IB/IIA/IIB/IIIA/IIIB/IIIC/IV/IVA/IVB).

The configuration selected for the MI methods using MICE corresponds to the default settings of the R package ‘mice’ [23]. For the JM, a burn of 500 updates and between-imputation updates of 500 were set [13].

### Performance measures

The Cox proportional hazards analysis was performed on each of the imputed datasets (with ECOG-PS = 0 as reference category). Thereafter, the results of the Cox proportional hazards analysis were combined using Rubin’s rules and were compared to the ‘true’ log(HR) of the randomly drawn complete dataset.

As the primary interest of this simulation study was the bias of the log(HR), the number of simulation replications per scenario (n_sim_) was determined based on a desired Monte Carlo standard error (MCSE) for the bias estimate. According to Morris et al., this is defined as MCSE(Bias)=sqrt(Var(𝜃_regr_)/(n_sim_)) [17]. Based on the study by Sy and Taylor, it was assumed that the variance of the estimated regression coefficient ($\:\widehat{\theta\:}$_regr_) would be ≤ 0.16 (corresponding to a standard error (SE) of 0.4, the largest SE expected for an ordinal variable with sample sizes of at least *N* = 500 [24, 25]), and an MCSE(bias) ≤ 0.016 (10% of the expected estimate [10]) was considered appropriate [22]. The number of simulation replications per scenario n_sim_ was calculated with the formula MCSE(Bias) =$\:\sqrt{\frac{\mathrm{V}\mathrm{a}\mathrm{r}\left(\widehat{{\uptheta\:}}\mathrm{r}\mathrm{e}\mathrm{g}\mathrm{r}\right)}{\:\mathrm{n}\mathrm{s}\mathrm{i}\mathrm{m}}}$ as n_sim_ = 625 results. In line with previous studies and to ensure sufficient precision, n_sim_ was set to 1,000 [13, 26, 27].

To evaluate the performance of the MI methods, the absolute and relative bias (AB and RB), mean-squared error (MSE), the average width (AW) of the 95% confidence interval (CI) and the coverage rate (CR) of the (log) HR of the Cox proportional hazards model were assessed. The performance parameters were calculated according to Morris et al. (see Table 6, [17]). An acceptable threshold of 10% bias was defined to compare the different MI methods.

The imputation-generating process was evaluated for validity and plausibility by examining the means, standard deviations (SD), medians, minimums, and maximums of the observed and imputed ECOG-PS values for each scenario and simulation repetition. The plausibility of the imputed values was assessed by checking whether the ECOG-PS values fell within the defined range. Additionally, the differences in mean values of the imputed and observed ECOG-PS values (mean_diff_ = mean_imp_ - mean_obs_) across all simulation runs were examined to compare the validity of the different MI methods.

The analyses were performed with R statistical programming language (Version 4.4.1) with the R packages ‘dplyr’ and ‘tidyr’ for data processing [28, 29], ‘jmv’, ‘ggplot2’, ‘ggcorrplot’, and ‘survminer’ for descriptive analysis and visualization [30–33], ‘mice’, ‘MASS’, ‘jomo’ and ‘mitml’ for MI, ‘survival’ for the Cox proportional hazards analysis [23, 34–37], and ‘parallel’ for parallel computations [38].

## Results

When comparing the complete cases with the original dataset (all lung cancer cases, *N* = 60,444), it was found that cases with complete data (*N* = 13,901) were more likely to be diagnosed in the years 2021 to 2022 (47.6% among all cases vs. 60.1% among complete cases, see Table 2). The proportion of deceased cases was lower in the complete-case group, at 61.4% compared to 70.5%, and the median survival time was 14 months compared to 10 months. An imbalance in the distribution of ECOG-PS categories was observed, with categories 2 to 4 in particular exhibiting low prevalence. Notably, the number of observed cases with ECOG-PS = 4 was very low with *n* = 13 (2.6% of *n* = 500), *n* = 27 (2.7% of *n* = 1,000) and *n* = 125 (2.5% of *n* = 5,000).

**Table 2:** Descriptive statistics of all lung cancer cases, the complete cases and the comparative truth with N=5,000, N=1,000 and N=500

	All lung cancer cases *N* = 60,444	Complete lung cancer cases *N* = 13,901	Comparative truth with *N* = 5,000	Comparative truth with *N* = 1,000	Comparative truth with *N* = 500
Age, mean (±SD)	69.5 (±10.3)	68.3 (±9.5)	68.2 (±9.5)	68.9 (±9.4)	67.9 (±10.0)
Missing, *n* (%)	0 (0)	0 (0)	0 (0)	0 (0)	0 (0)
Sex male, *n* (%)	34,856 (57.7)	8,108 (58.3)	2,898 (58.0)	602 (60.2)	272 (54.4)
Missing	1 (<0.1)	0 (0)	0 (0)	0 (0)	0 (0)
Diagnosis year, *n* (%)					
2019	16,346 (27.0)	2,726 (19.6)	1,002 (20.0)	205 (20.5)	89 (17.8)
2020	15,369 (25.4)	2,819 (20.3)	1,000 (20.0)	191 (19.1)	105 (21.0)
2021	15,703 (26.0)	3,857 (27.7)	1,365 (27.3)	281 (28.1)	149 (29.8)
2022	13,026 (21.6)	4,499 (32.4)	1,633 (32.7)	323 (32.3)	157 (31.4)
Missing	0 (0)	0 (0)	0 (0)	0 (0)	0 (0)
ECOG performance status, *n* (%)					
0	6,086 (10.1)	3,498 (25.2)	1,276 (25.5)	237 (23.7)	119 (23.8)
1	12,067 (20.0)	6,790 (48.8)	2,442 (48.8)	495 (49.5)	250 (50.0)
2	4,591 (7.6)	2,272 (16.3)	821 (16.4)	164 (16.4)	89 (17.8)
3	2,195 (3.6)	1,005 (7.2)	336 (6.7)	77 (7.7)	29 (5.8)
4	779 (1.3)	336 (2.4)	125 (2.5)	27 (2.7)	13 (2.6)
Missing	34,726 (57.5)	0 (0)	0 (0)	0 (0)	0 (0)
Survival Status, *n* (%)					
Alive	17,805 (29.5)	5,369 (38.6)	1,932 (38.6)	340 (34.0)	181 (36.2)
Dead	42,639 (70.5)	8,532 (61.4)	3,068 (61.4)	660 (66.0)	319 (63.8)
Missing	0 (0)	0 (0)	0 (0)	0 (0)	0 (0)
Survival in months (median, IQR)	10 (2-25)	14 (5-27)	15 (5-27)	13 (5-25)	14 (4-27)
Missing, *n* (%)	26 (<0.1)	0 (0)	0 (0)	0 (0)	0 (0)
Topography ICD-10, *n* (%)					
C34.0 - main bronchus	4,797 (7.9)	1,206 (8.7)	441 (8.8)	101 (10.1)	45 (9.0)
C34.1 - upper lobe, bronchus or lung	23,105 (38.2)	6,441 (46.3)	2,277 (45.5)	487 (48.7)	239 (47.8)
C34.2 - middle lobe, bronchus or lung	2,247 (3.7)	611 (4.4)	209 (4.2)	33 (3.3)	15 (3.0)
C34.3 - lower lobe, bronchus or lung	12,941 (21.4)	3,716 (26.7)	1,351 (27.0)	236 (23.6)	129 (25.8)
C34.8 - overlapping lesions	2,321 (3.8)	574 (4.1)	199 (4.0)	43 (4.3)	20 (4.0)
C34.9 - bronchus or lung, unspecified	15,033 (24.9)	1,353 (9.7)	523 (10.5)	100 (10.0)	52 (10.4)
Missing	0 (0)	0 (0)	0 (0)	0 (0)	0 (0)
Grading, *n* (%)					
G1	1,355 (2.2)	528 (3.8)	180 (3.6)	24 (2.4)	21 (4.2)
G2	11,497 (19.0)	4,566 (32.8)	1,670 (33.4)	321 (32.1)	151 (30.2)
G3	20,136 (33.3)	7,893 (56.8)	2,824 (56.5)	582 (58.2)	288 (57.6)
G4	2,061 (3.4)	914 (6.6)	326 (6.5)	73 (7.3)	40 (8.0)
Missing	25,395 (42.0)	0 (0)	0 (0)	0 (0)	0 (0)
Morphology ICD-O*, *n* (%)					
Adenocarcinoma	23,665 (39.1)	7,036 (50.6)	2,501 (50.0)	497 (49.7)	257 (51.4)
Squamous cell carcinoma	11,528 (19.1)	3,993 (28.7)	1,461 (29.2)	283 (28.3)	133 (26.6)
Large cell carcinoma	142 (0.2)	35 (0.3)	14 (0.3)	1 (<0.01)	2 (0.4)
Small cell carcinoma	8,250 (13.7)	1,870 (13.5)	681 (13.6)	149 (14.9)	74 (14.8)
Neuroendocrine tumor	2,140 (3.5)	560 (4.0)	177 (3.5)	39 (3.9)	18 (3.6)
Adenosquamous carcinoma	842 (1.4)	299 (2.2)	124 (2.5)	21 (2.1)	10 (2.0)
Sarcomatoid carcinoma	314 (0.5)	85 (0.6)	35 (0.7)	7 (0.7)	4 (0.8)
Salivary gland-type tumor	84 (0.1)	18 (0.1)	5 (0.01)	3 (0.3)	2 (0.4)
Other tumors	79 (0.1)	5 (<0.1)	2 (<0.01)	0 (0)	0 (0)
Not further specified	13,400 (22.2)	0 (0)	0 (0)	0 (0)	0 (0)
Tumor Stage, *n* (%)					
IA1	799 (1.3)	270 (1.9)	108 (2.2)	15 (1.5)	14 (2.8)
IA2	2,306 (3.8)	803 (5.8)	271 (5.4)	54 (5.4)	39 (7.8)
IA3	1,529 (2.5)	605 (4.4)	200 (4.0)	39 (3.9)	13 (2.6)
IB	1,656 (2.7)	695 (5.0)	248 (5.0)	55 (5.5)	29 (5.8)
IIA	648 (1.1)	270 (1.9)	88 (1.8)	26 (2.6)	15 (3.0)
IIB	2,608 (4.3)	1,092 (7.9)	407 (8.1)	75 (7.5)	36 (7.2)
IIIA	4,239 (7.0)	1,691 (12.2)	652 (13.0)	102 (10.2)	60 (12.0)
IIIB	3,427 (5.7)	1,224 (8.8)	422 (8.4)	81 (8.1)	36 (7.2)
IIIC	1,508 (2.5)	545 (3.9)	213 (4.3)	41 (4.1)	18 (3.6)
IV	3,957 (6.6)	779 (5.6)	265 (5.3)	61 (6.1)	29 (5.8)
IVA	8,108 (13.4)	2,579 (18.6)	906 (18.1)	198 (19.8)	94 (18.8)
IVB	9,658 (16.0)	3,348 (24.1)	1,220 (24.4)	253 (25.3)	117 (23.4)
Missing	20,001 (33.1)	0 (0)	0 (0)	0 (0)	0 (0)
Intention of first-line therapy, *n* (%)					
No therapy reported	26,820 (44.4)	3,436 (24.7)	1,179 (23.6)	246 (24.6)	128 (25.6)
Palliative intention	16,799 (27.8)	5,044 (36.3)	1,823 (36.5)	384 (38.4)	193 (38.6)
Curative intention	14,278 (23.6)	5,421 (39.0)	1,998 (40.0)	370 (37.0)	179 (35.8)
Therapy reported, intention missing	2,547 (4.2)	0 (0)	0 (0)	0 (0)	0 (0)
First-line systemic therapy, *n* (%)					
No therapy reported	39,951 (66.1)	7,233 (52.0)	2,524 (50.5)	516 (51.6)	260 (52.0)
Yes – chemotherapy	13,183 (21.8)	4,334 (31.2)	1,589 (31.8)	302 (30.2)	148 (29.6)
Yes – immuno-chemotherapy	4,311 (7.1)	1,491 (10.7)	574 (11.5)	125 (12.5)	59 (11.8)
Yes – mono-immunotherapy	1,775 (2.9)	535 (3.8)	193 (3.9)	41 (4.1)	20 (4.0)
Yes – targeted Therapy	1,224 (2.0)	308 (2.2)	120 (2.4)	16 (1.6)	13 (2.6)
First-line radiotherapy, *n* (%)					
No therapy reported	47,935 (79.3)	9,982 (71.8)	3,610 (72.2)	728 (72.8)	357 (71.4)
Yes	12,509 (20.7)	3,919 (28.2)	1,390 (27.8)	272 (27.2)	143 (28.6)
First-line surgery, *n* (%)					
No surgery reported	44,545 (73.7)	9,377 (67.5)	3,335 (66.7)	692 (69.2)	343 (68.6)
Yes	15,899 (26.3)	4,524 (32.5)	1,665 (33.3)	308 (30.8)	157 (31.4)
Residual status after surgery, *n* (%)					
No surgery reported	44,545 (73.7)	9,377 (67.5)	3,335 (66.7)	692 (69.2)	343 (68.6)
R1	907 (1.5)	368 (2.6)	138 (2.8)	28 (2.8)	13 (2.6)
R0	9,217 (15.3)	4,156 (29.9)	1,527 (30.5)	280 (28.0)	144 (28.8)
Surgery reported, residual status missing	5,775 (9.6)	0 (0)	0 (0)	0 (0)	0 (0)

*See additional file 1, Supplementary Table S2 for classification of lung cancer tumors by ICD-O-3 morphology codes

### Performance evaluation

Separate figures are provided for each performance parameter and missingness mechanism (see Figs. 2, 3, 4, 5 and 6 and additional file 1, Supplementary Figures S1 to S10). To ensure comparability, all panels were displayed using identical axis scales across sample sizes and ECOG-PS categories. Consequently, in a few scenarios the full range of values could not be displayed. Therefore the mean values of all performance parameters are additionally provided in Supplementary Table S3 (see additional file 1).

**Fig. 2:**
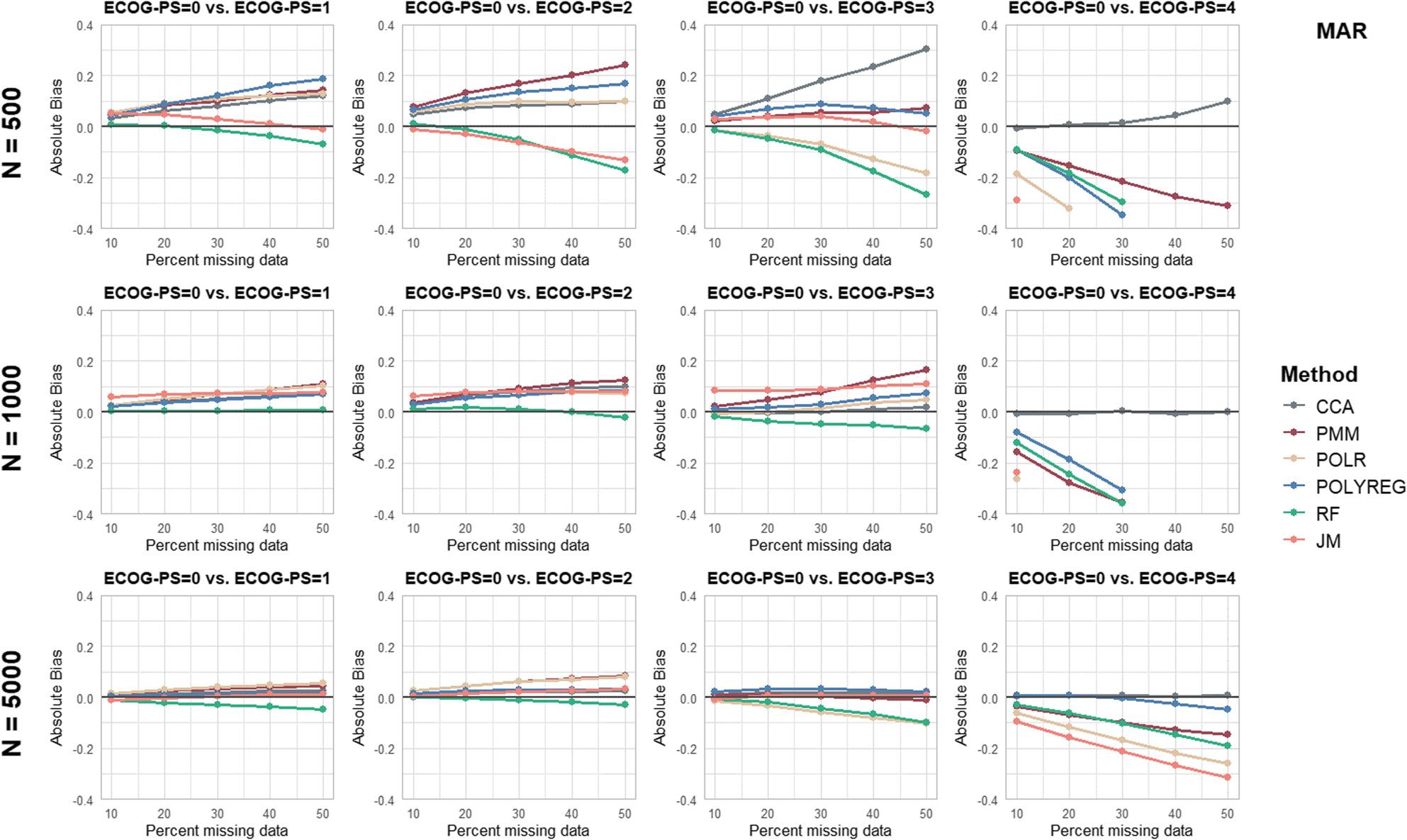
Results of the performance parameter ‘Absolute Bias’ for Missing At Random (MAR) scenarios

**Fig. 3:**
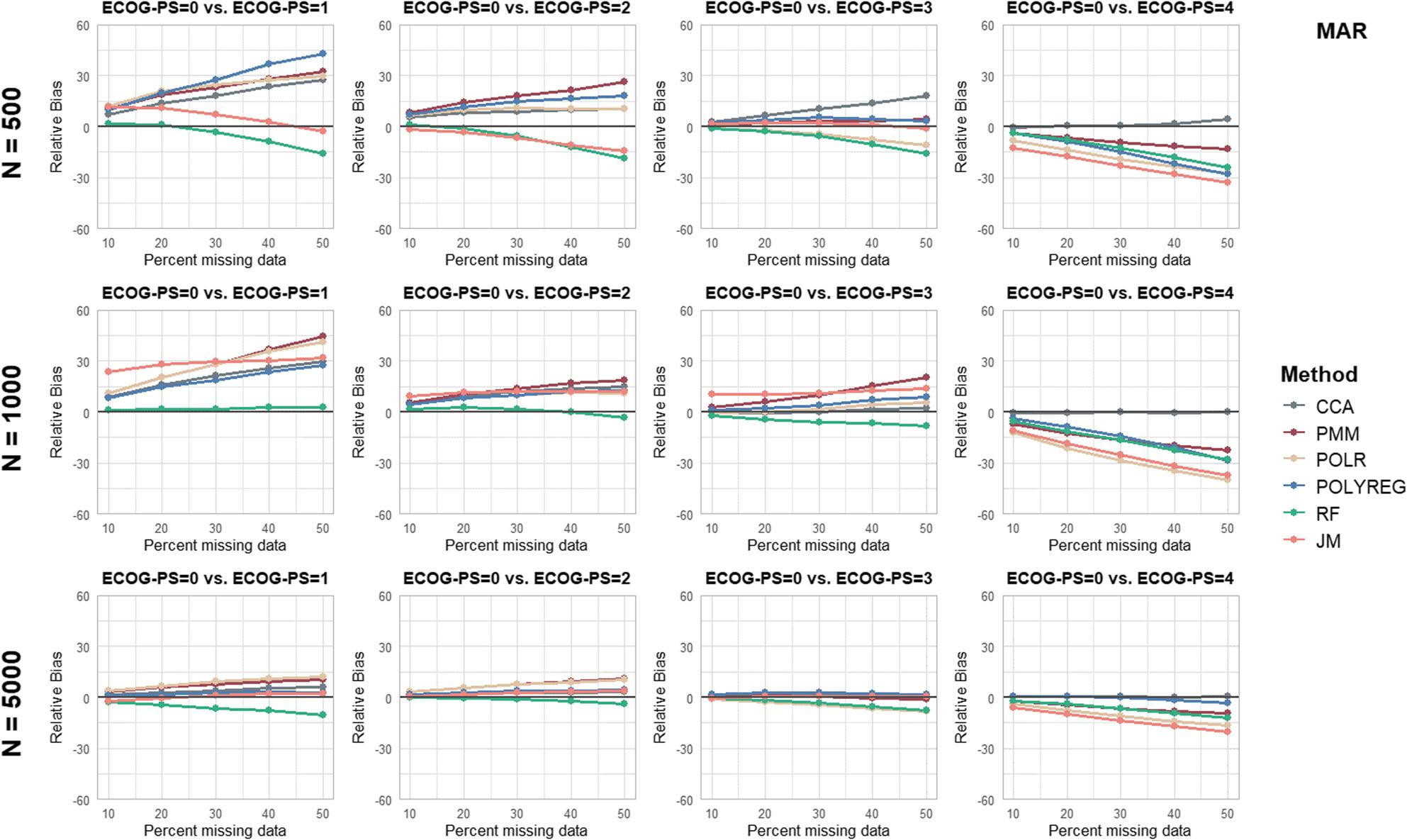
Results of the performance parameter ‘Relative Bias’ for Missing At Random (MAR) scenarios

**Fig. 4:**
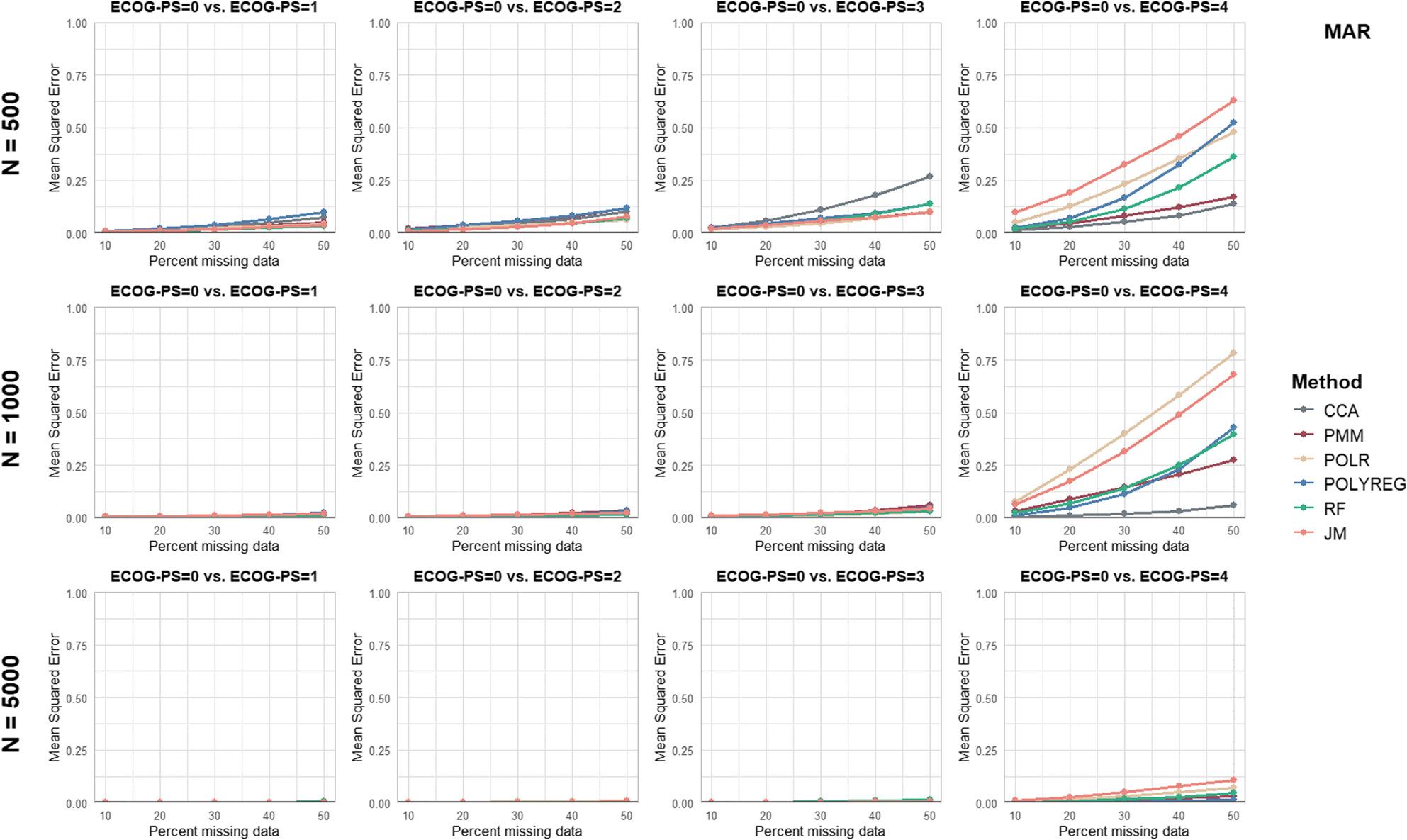
Results of the performance parameter ‘Mean Squared Error’ for Missing At Random (MAR) scenarios

**Fig. 5:**
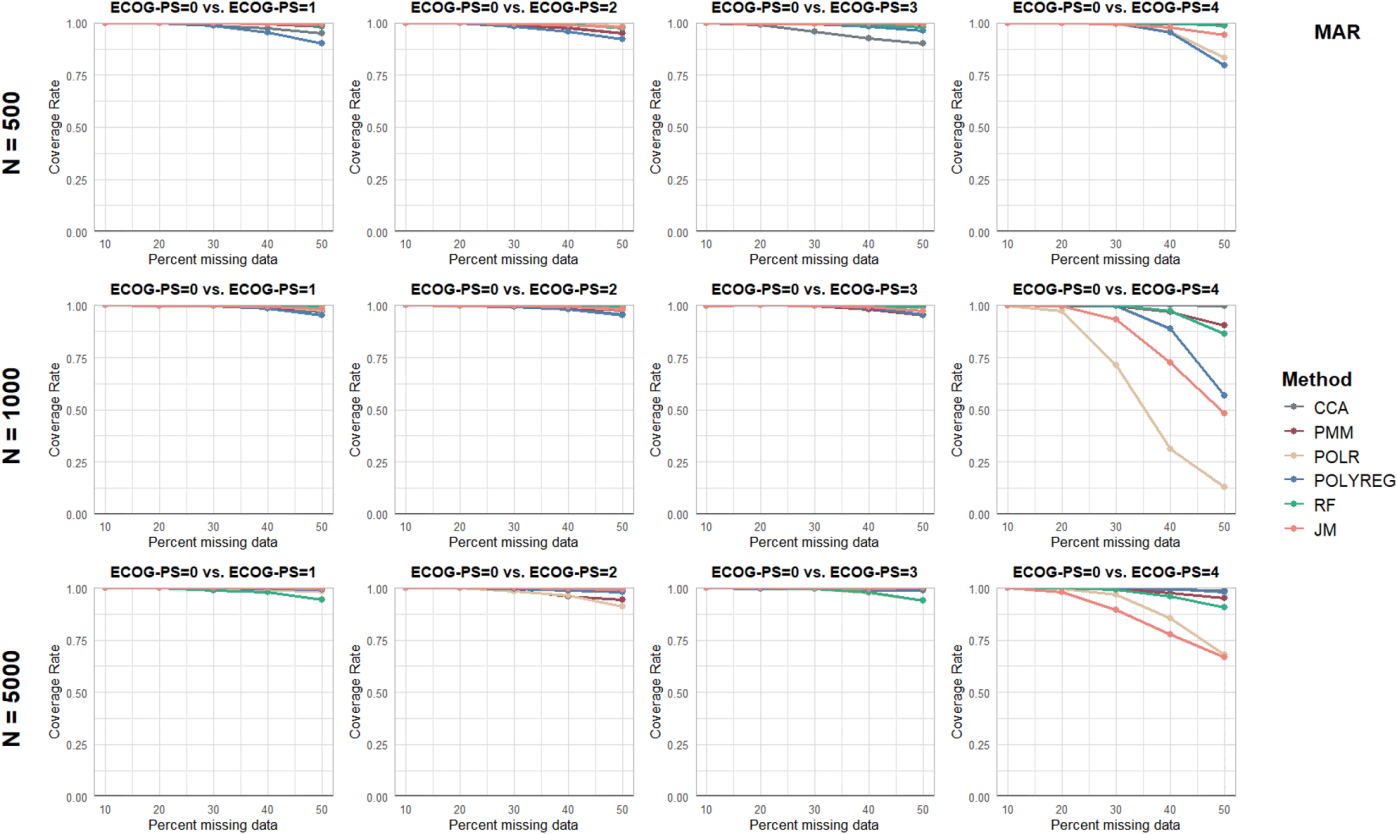
Results of the Performance Parameter ‘Coverage Rate’ for Missing At Random (MAR) scenarios

**Fig. 6:**
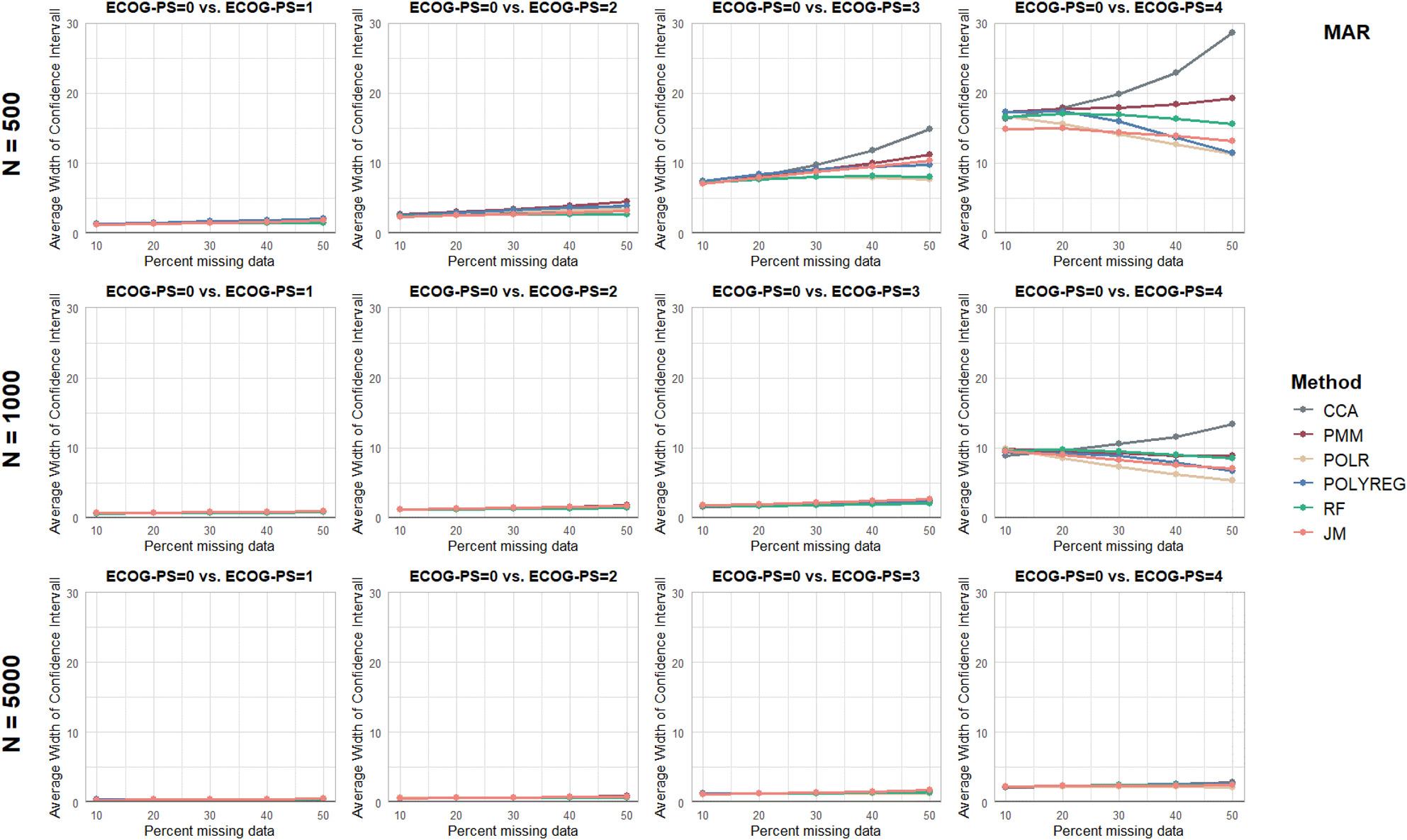
Results of the Performance Parameter ‘Average Width of the 95% CI’ for Missing At Random (MAR) scenarios

In general, it was found in most scenarios that the AB of the log(HR) increased with an increasing proportion of missing values. Furthermore, the AB increased with higher ECOG-PS categories compared to the reference category ECOG-PS = 0. However, despite the large AB, the RB for ECOG-PS = 0 vs. ECOG-PS = 4 is relatively smaller due to the larger true log(HR). In contrast, the RB for ECOG-PS = 0 vs. ECOG-PS = 1 is comparatively larger.

For *n* = 500 and *n* = 1,000 with 30% or more missing data, the log(HR) were extremely biased for the comparison of ECOG-PS = 0 vs. ECOG-PS = 4. Accordingly, no MI method achieved acceptable bias estimates. The missingness mechanisms MCAR and MNAR showed similar results with regard to bias across the three different sample sizes compared to the MAR scenarios.

With a sample size of *n* = 5,000, MICE with POLYREG yielded acceptable bias estimates across all missingness mechanisms and proportions, with the highest RB value of -5.9% under MCAR with 50% missing data. MICE with RF performed similarly well, yielding acceptable bias estimates for all missingness mechanisms with up to 40% missing values and 50% missing data under MNAR. MICE with PMM showed acceptable bias estimates of the log(HR) across all ECOG-PS categories for up to 30% missing values across all missingness mechanisms. The JM and POLR had biased estimates for ECOG-PS = 4, ranging from − 10.3% to -22.4% across all missingness mechanisms with 30% or more missing data. The CCA performed well with a sample size of *N* = 5,000 across all missingness mechanisms and proportions. In contrast, at sample sizes of *N* = 500 and *N* = 1,000 and missingness proportions of 20% or higher, biased regression coefficient estimates were observed in outcome-dependent MAR scenarios, while MSE remained consistently low.

Like bias, MSE increased with the missingness proportions and across the ECOG-PS categories.

Across all sample sizes, missingness mechanisms, and missingness proportions, the CR was close to or exceeded the nominal level of 95% for ECOG-PS categories 1 to 3, and was often close to 100%, indicating overcoverage. For ECOG-PS category 4 (ECOG-PS = 0 vs. ECOG-PS = 4), the CR were notably lower for MICE with POLR and POLYREG, and the JM, particularly with increasing proportion of missing values.

For the sample size of *n* = 500 and ECOG-PS category 4, very large AW of the 95%CI were found for all MI methods. In these scenarios, CCA was associated with on average wider 95%CI compared to the MI methods, reflecting increased uncertainty arising from the reduced effective sample size under CCA. For the *n* = 5,000 cases, narrow 95%CI were observed across all proportions of missing values, MI methods, missingness mechanisms, and ECOG-PS categories.

### Evaluation of the imputation-generating process

Overall, the whole simulation procedure required 725.9 h (Intel(R) Core(TM) i7-14700, 20 CPUs, 28 vCPUs, base frequency 2.10 GHz, 31.7 GB of memory). The R code is provided in additional file 2.

All MI methods provided valid values across all scenarios, except in one scenario (MCAR, 50% missing values with a sample size of *n* = 500) in a single replicate, the CCA failed to produce the HR and its 95% CI for the comparison of ECOG-PS = 0 and ECOG-PS = 4. As a result, missing values for the performance parameters in this particular scenario were produced and the AW of the 95%CI for this scenario was infinite for MICE with PMM and MICE with RF.

The imputed values were plausible, ranging from 0 to 4. Generally, it was found that the scattering of the differences of the mean values decreased with an increasing sample size (see additional file 1, Supplementary Figures S11 to S13) and that the mean values of the imputed ECOG-PS values using MICE with RF were consistently smaller than the observed mean ECOG-PS values across all scenarios.

## Discussion

In Germany, data from population-based cancer registries are particularly valuable due to their high external validity, resulting from the statutory reporting obligation [39–41]. These data are collected using a standardized catalog of core items and tumor-specific modules (referred to as ‘*onkologischer Basisdatensatz’*, oBDS [41]), ensuring harmonization across all federal cancer registries. Despite these strengths, data completeness remains an issue with nearly 60% missing values of the ECOG-PS in this study, while tumor stage and tumor grading were missing in 33% and 42% of cases, respectively. Previous studies have focused on the evaluation of MI methods for missing tumor stage values.

Eisemann et al. compared four different imputation methods for missing tumor stage values in breast cancer cases based on cancer registry data: MICE with POLYREG, PMM, RF, and proportional sampling [12]. They examined five replications (n_sim_=5) of data MAR with 26% missing values in a large sample of 17,162 breast cancer cases, and compared individual imputed values to the deleted originals. They found that MICE with POLYREG yielded imputed stage values closest to the observed values [12]. MICE with PMM yielded results nearly as accurate as those obtained by POLYREG, while with RF imputation, estimations tended to have very large variances and were the most biased of all four methods [12].

Falcaro et al. investigated different MI methods with regard to estimating excess HR and net survival when covariate data, specifically the tumor stage, were missing [11]. In complete data of 44,461 colorectal cancer registry cases 30% of values of the tumor stage were set missing according to MCAR, covariate-dependent MAR and MAR (depending on age at diagnosis, survival time and the event indicator) with 100 replications [11]. They investigated four MI strategies, using POLR or POLYREG, and either the survival time and its logarithm, or the Nelson-Aalen estimate of the cumulative hazard [11]. They found that imputing the tumor stage using POLYREG with the Nelson-Aalen estimator performed best [11].

In line with those previous studies, this study was based on cancer registry data as real-world dataset, confirming their findings by demonstrating that MICE with POLYREG performed best in handling missing ordinal data [11, 12], highlighting its robustness across different levels of missingness proportions and mechanisms. Moreover, different sample sizes were considered, as MI methods generally yield more accurate estimates in larger sample sizes. While sample sizes of 500 and 1,000 would not typically be considered small from a statistical perspective, such sample sizes are often regarded as small in cancer epidemiology [42, 43]. Depending on the cancer entity of interest, sample sizes might not always be large, potentially reducing the accuracy of MI methods. Interestingly, it was found in this study that the prevalence and distribution of the categories of the ordinal variable are of considerable importance, as for low-prevalence categories and smaller sample sizes with more than 30% missing values, the log(HR) were extremely biased across all examined MI methods. Importantly, the bias diminished with increasing sample size, indicating finite-sample effects rather than systematic bias of the imputation methods.

Following a reviewer’s suggestion, additional simulations with a sample size of *N* = 3,000 for the MAR scenarios were conducted to further investigate the transition in performance between moderate and large sample sizes. The corresponding results, reported in Additional File 3, are very similar to those obtained for *N* = 5,000, indicating that the large-sample behavior observed at *N* = 5,000 is already largely approached at *N* = 3,000.

Similarly, while CCA was biased in most MAR scenarios at sample sizes of *N* = 500 and *N* = 1,000 with missingness proportions of 20% or higher, no MI method consistently outperformed CCA under these conditions. As missingness was restricted to a single covariate and the outcome was fully observed, CCA yielded unbiased estimates at *N* = 5,000 across all missingness mechanisms, as well as at *N* = 3000 under MAR. In these settings, excluding individuals with missing information in a single covariate did not induce systematic bias.

The deviations observed at smaller sample sizes, affecting both CCA and MI approaches, are therefore best interpreted as finite-sample effects rather than as consequence of the missingness mechanism itself. Accordingly, our results indicate that, when only one covariate is missing and the outcome is fully observed, MI may yield narrower 95%CI in some settings, however, CCA performs at least as well as MI in terms of bias and MSE. Under limited sample sizes and selective missingness, MI may provide little to no benefit and can introduce additional variability, as observed in several simulation scenarios. As this study focused on a controlled simulation setting with missingness restricted to a single covariate in order to compare the behavior of commonly used MI methods, our findings are not directly transferable to the full complexity of real-world cancer registry data.

The great advantage of the RF method is its robustness and accuracy in imputations, even in the presence of complex interactions, multicollinearity, outliers, skewed distributions, and non-linear relationships [21, 44, 45]. However, for categorical variables, RF tend to bias predictions toward the larger category, particularly when class distributions are highly imbalanced and smaller categories are sparsely represented, leaving insufficient data to accurately model patterns for these categories [21, 46, 47]. This ‘class imbalance problem’ is a known limitation of the RF imputation [46], aligning with the findings of this study, with RF consistently imputing lower ECOG-PS values than those observed across all scenarios. Nonetheless, RF performed comparatively well in comparison to MICE with POLYREG.

In contrast to MICE, the JM assumes a joint multivariate model for all partially observed data, theoretically leading to unbiased inferences under MAR [13, 48]. The JM and MICE with ordered logistic regression (POLR) were compared on real data of breast cancer patients. In 1,000 sampled datasets, they introduced missing values in tumor grade (20% MAR), menopausal status (20% MAR) and the number of active nodes (20% MCAR). The authors reported close to 95% coverage in an Cox proportional hazards model for both imputation methods and concluded that in practice, the two methods can be used interchangeably [13]. In the present study, the JM demonstrated poor performance.

The performance of MICE with POLR, which relies on the proportional odds assumption was similarly poor [49]. Consistent with the conclusions of Falcaro et al., the results of our study indicate that an ordinal model (POLR) might not be suitable for imputing missing ECOG-PS values in cancer registry datasets [11].

One limitation of our study was the data-generation mechanism, reducing the original cancer registry dataset by over 75%, which may result in samples that do not fully capture all relevant real-world conditions [16]. Another limitation was that the generation of the MNAR missingness mechanism could not be fully implemented due to the observed data structure, i.e. the missing values were not exclusively related to the missing value itself. Ultimately, an MAR-like mechanism was generated because the survival status was still associated with the ECOG-PS. This fact was also highlighted by Schouten and Vink, stating that assuming MNAR missingness on highly correlated data may be unnecessary as the essence of the missing data may be sufficiently covered by the observed information [50]. Finally, this study focused on the Cox proportional hazards regression model, assuming that the hazard function was constant over time and that censoring was non-informative without explicit testing in the various simulation scenarios.

Although zero-inflated ordinal covariates can arise in practice and require specialized modeling approaches, the ECOG-PS distribution in this study was not dominated by zeros, and standard ordinal imputation methods were therefore appropriate.

Future research could expand this simulation framework to include additional MI methods not analyzed here, such as imputation strategies specifically tailored to zero-inflated ordinal data [51], as well as the nonparametric multiple imputation approach proposed by Hsu and Yu, which may offer advantages over traditional MICE [52]. In this approach, two working regression models are used to derive two predictive scores: one for predicting the missing covariates and one for predicting the missing probabilities. These scores are used to select an imputing set for each observation with missing covariate data, from which the missing values are then non-parametrically imputed [52]. In addition, future research could extend the present simulation framework to investigate the performance of MI methods in alternative inferential settings, including multivariate missingness or scenarios involving variable selection in Cox regression models with partially observed covariates [53].

## Conclusion

In conclusion, this study highlights the impact of sample size and class imbalance on MI performance, as severe bias might be introduced when sample sizes are smaller and prevalence of categories is low, indicating finite-sample effects rather than systematic bias of the imputation methods. In line with previous studies, our study confirmed that MICE with POLYREG yielded the best performance when handling missing ordinal cancer registry data. However, when missingness is restricted to a single covariate and the outcome is fully observed, MI may offer little to no benefit compared to CCA and can introduce additional variability. The findings stress the need for further research in the context of MI of ordinal data in time-to-event analysis, and practical recommendations to address these challenges and bridge the gap between theory and real-world application. 

## Supplementary Information

Additional file 1.

Additional file 2. R Code for the simulation.

Additional file 3. Simulation scenarios for N=3,000 with MAR missingness.

## Data Availability

Due to the statutory regulations of the North Rhine-Westphalia State Cancer Registry, data may not be passed on to third parties. A request for data usage can be submitted directly to the North Rhine-Westphalia State Cancer Registry or forwarded to the responsible authorities via the corresponding author.
