# Supplementary material for: Multiple imputation for missing values in ordinal variables from cancer registry data when performing Cox proportional hazards regression
Source: BMC Med Res Methodol. 2026 Feb 6;26:47. doi: 10.1186/s12874-026-02790-8 (PMC12930733; doi:10.1186/s12874-026-02790-8)
Supplement: Supplementary file 1 — Additional file 1. [file 12874_2026_2790_MOESM1_ESM.pdf]

Supplementary Table S1 – Multivariable logistic regression model for missing ECOG-PS values (N=22,956)

**Variable**	***β* regression coefficient**	**Odds Ratio**	**95% Confidence Interval**	***p*-value**
Age	-0.005	0.995	0.992, 0.998	0.002
Sex	0.001	1.00	0.95, 1.06	0.975
Diagnosis Year	-0.478	0.62	0.60, 0.64	< 0.001
Survival Status	0.083	1.09	0.99, 1.19	0.073
Nelson Aalen Estimate	0.147	1.16	1.03, 1.30	0.011
C34.0	-0.061	0.94	0.84, 1.06	0.305
C34.1	-0.032	0.97	0.90, 1.05	0.434
C34.3	-0.103	0.90	0.83, 0.98	0.020
Grading	-0.060	0.94	0.90, 0.99	0.013
Adenocarcinoma	0.045	1.05	0.94, 1.17	0.422
Squamous cell carcinoma	-0.033	0.97	0.86,1.09	0.577
Small cell carcinoma	0.097	1.10	0.96, 1.26	0.160
Tumor stage	-0.021	0.98	0.97, 0.99	< 0.001
Intention of first-line therapy	-0.163	0.85	0.80, 0.90	< 0.001
First-line systemic therapy	-0.143	0.87	0.84, 0.90	< 0.001
First-line radiotherapy	-0.291	0.75	0.69, 0.80	< 0.001
Residual status after surgery	0.001	1.00	0.95, 1.05	0.970

Supplementary Table S2 – Classification of lung cancer tumors by ICD-O-3 morphology codes

**Histologic subtype**	**ICD-O-3 morphology code(s)**
Adenocarcinoma	8050/3, 8140/3, 8141/3, 8144/3, 8190/3, 8201/3, 8210/3, 8211/3, 8230/3, 8243/3, 8244/3, 8250/3, 8251/3, 8252/3, 8253/3, 8254/3, 8255/3, 8256/3, 8257/3, 8260/3, 8262/3, 8263/3, 8265/3, 8310/3, 8323/3, 8333/3, 8430/3, 8440/3, 8480/3, 8481/3, 8490/3, 8550/3, 8551/3, 8570/3, 8572/3, 8574/3, 8576/3
Squamous cell carcinoma	8052/3, 8054/3, 8070/3, 8071/3, 8072/3, 8073/3, 8074/3, 8075/3, 8076/3, 8078/3, 8082/3, 8083/3, 8084/3, 8085/3, 8086/3, 8120/3, 8123/3
Large cell carcinoma	8012/3, 8021/3
Small cell carcinoma	8002/3, 8041/3, 8042/3, 8043/3, 8045/3
Neuroendocrine tumor	8013/3, 8240/3, 8245/3, 8246/3, 8249/3
Adenosquamous carcinoma	8560/3
Sarcomatoid carcinoma	8003/3, 8004/3, 8022/3, 8031/3, 8032/3, 8033/3, 8972/3, 8940/3, 8980/3
Salivary gland-type tumor	8023/3, 8044/3, 8200/3, 8562/3
Other tumors	8158/3, 8680/3, 8800/3, 8801/3, 8802/3, 8804/3, 8805/3, 8811/3, 8815/3, 8830/3, 8840/3, 8842/3, 8854/3, 8890/3, 8901/3, 9040/3, 9041/3, 9120/3, 9130/3, 9133/3, 9364/3, 9712/3
Not further specified	8000/3, 8001/3, 8010/3, 8020/3, 8046/3

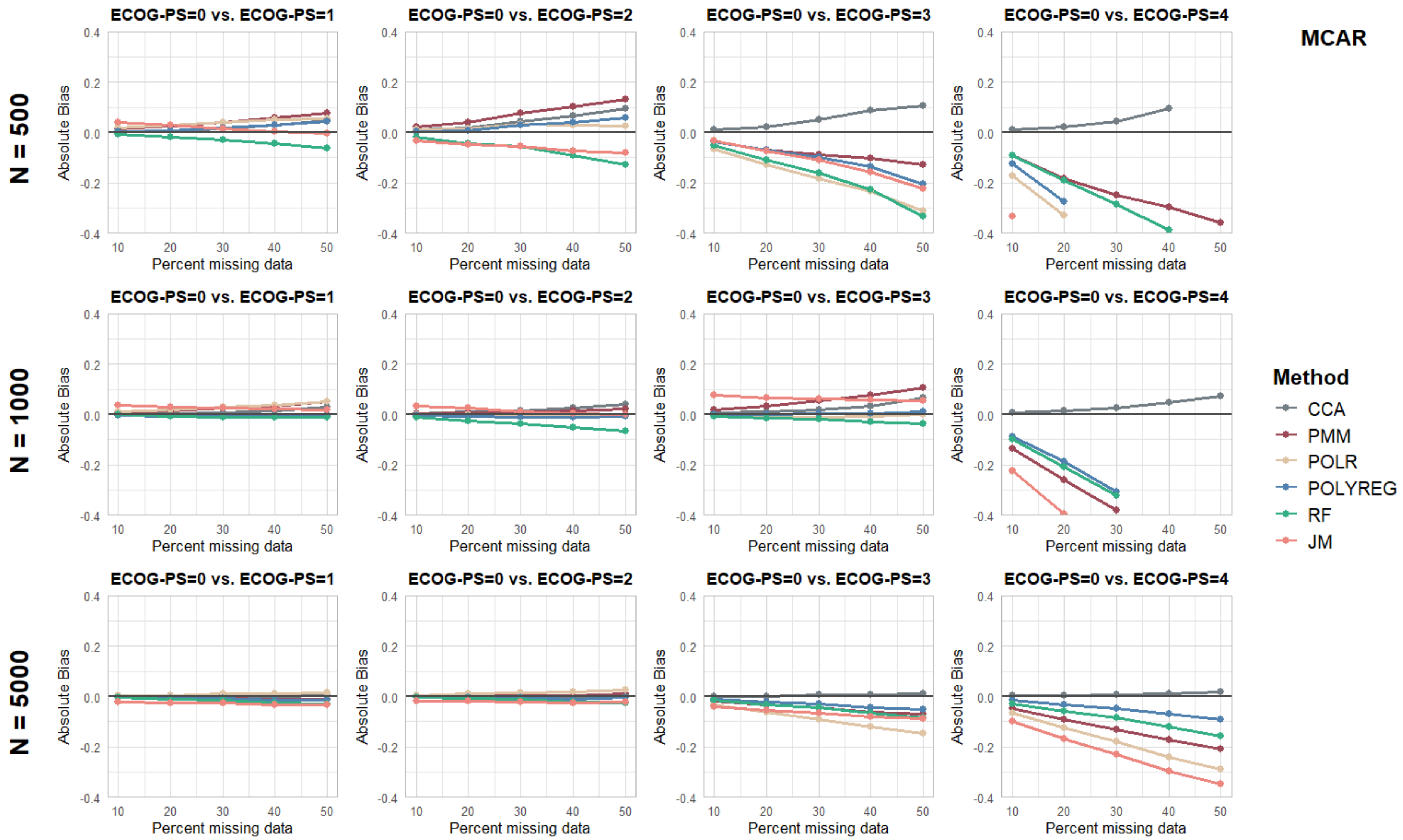

Supplementary Figure S1 – Results of the Performance Parameter 'Absolute Bias' (Missingness Mechanism: MCAR)

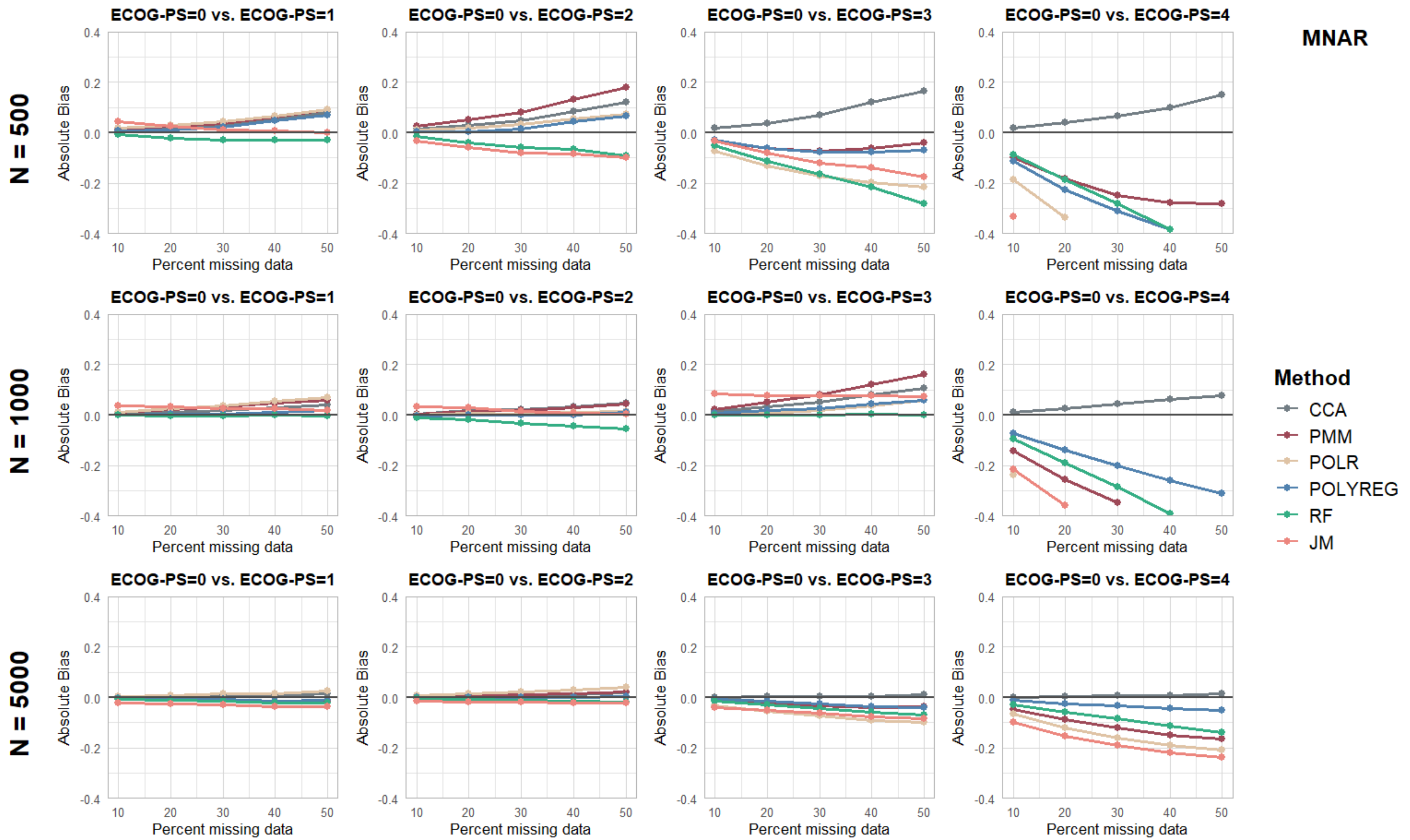

Supplementary Figure S2 – Results of the Performance Parameter 'Absolute Bias' (Missingness Mechanism: MNAR)

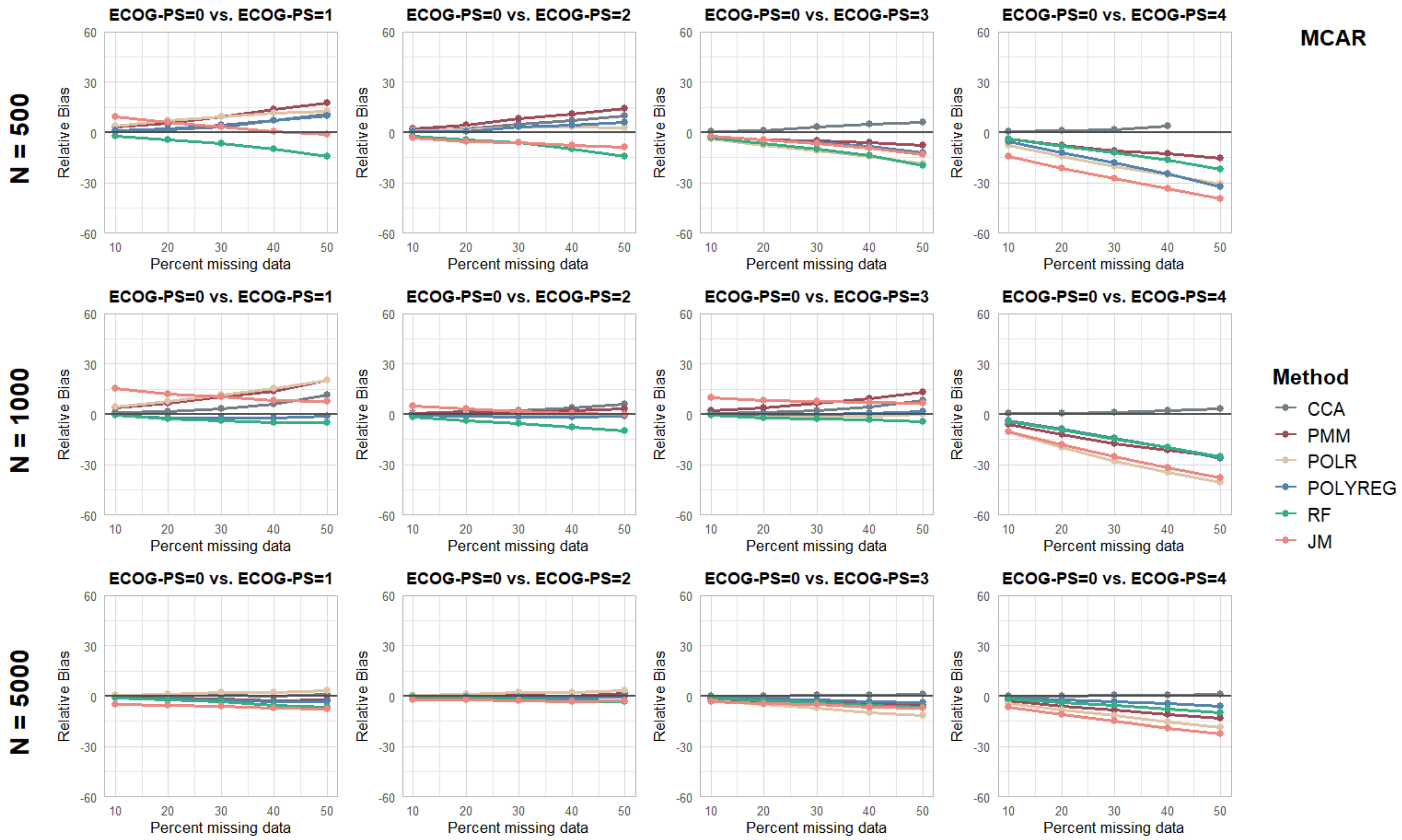

Supplementary Figure S3 – Results of the Performance Parameter 'Relative Bias' (Missingness Mechanism: MCAR)

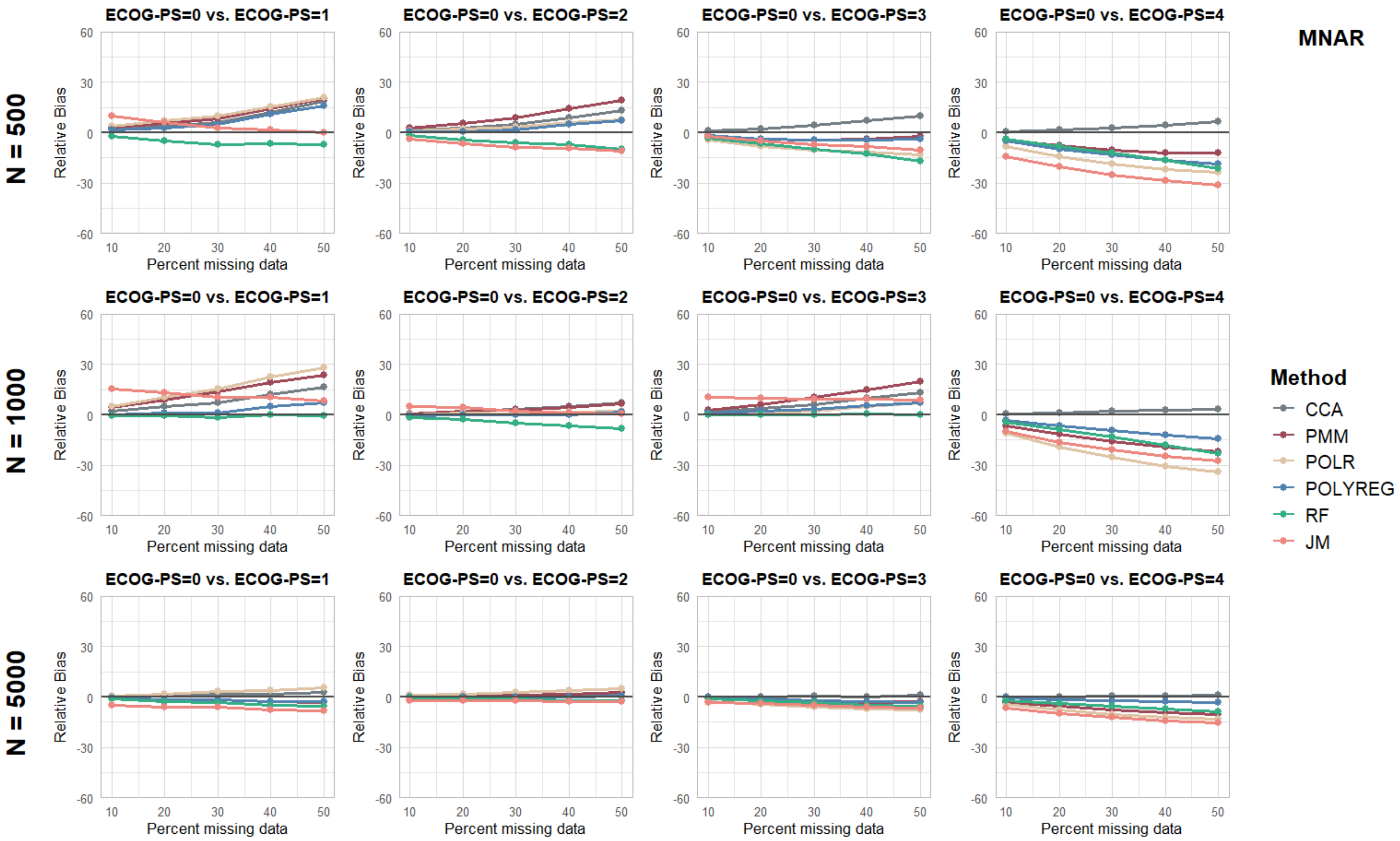

Supplementary Figure S4 - Results of the Performance Parameter 'Relative Bias' (Missingness Mechanism: MNAR)

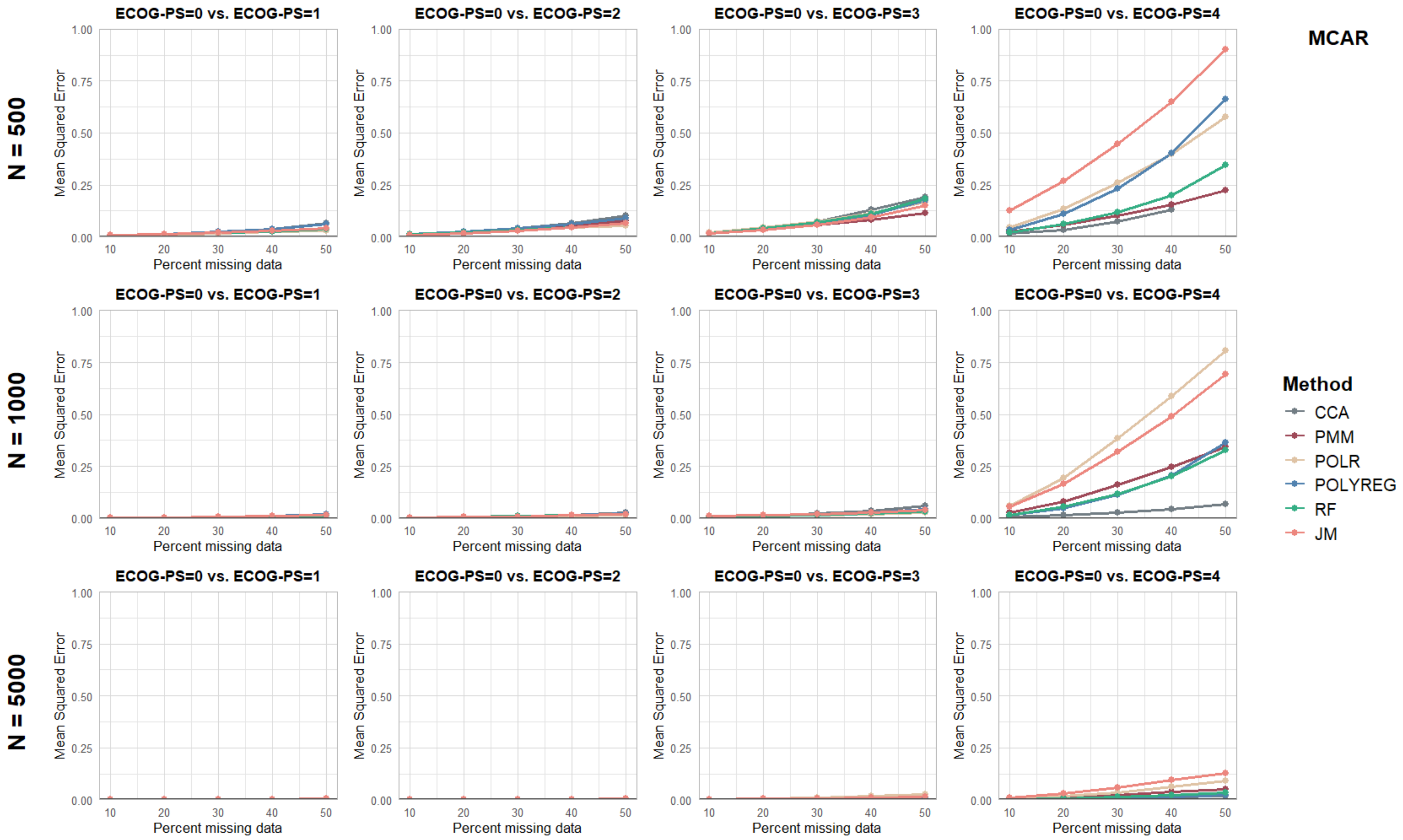

Supplementary Figure S5 – Results of the Performance Parameter 'Mean Squared Error' (Missingness Mechanism: MCAR)

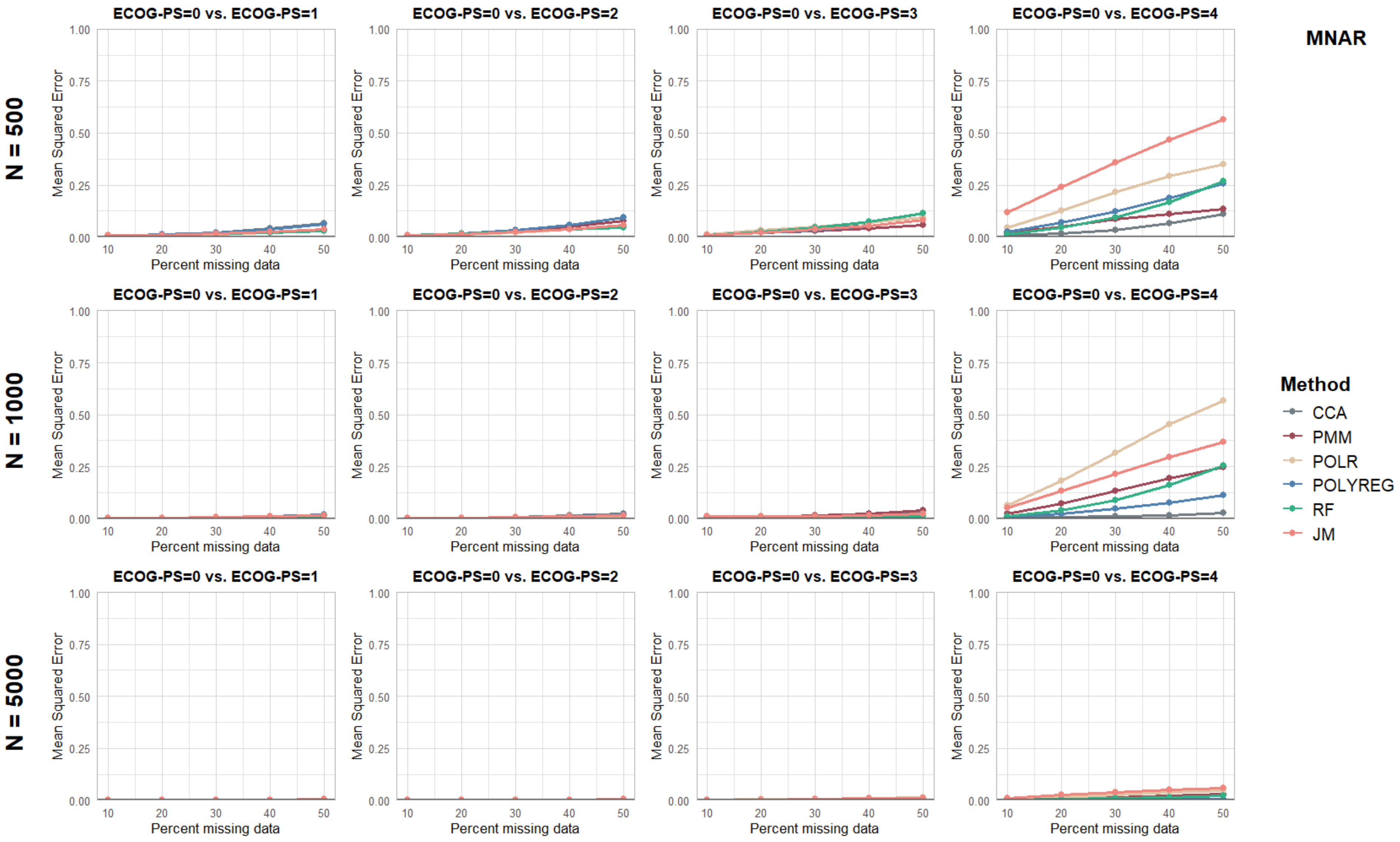

Supplementary Figure S6 – Results of the Performance Parameter 'Mean Squared Error' (Missingness Mechanism: MNAR)

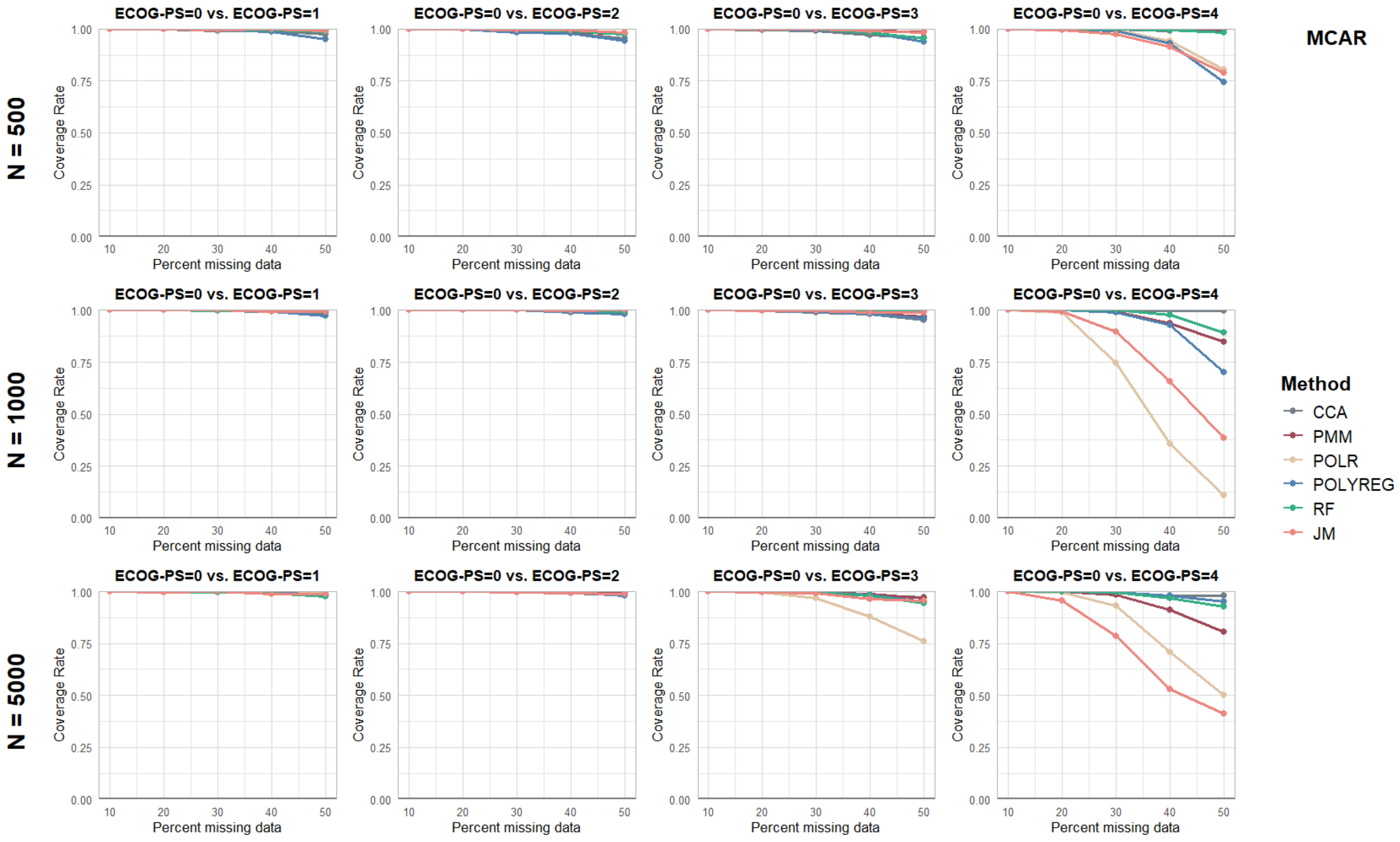

Supplementary Figure S7 – Results of the Performance Parameter 'Coverage Rate' (Missingness Mechanism: MCAR)

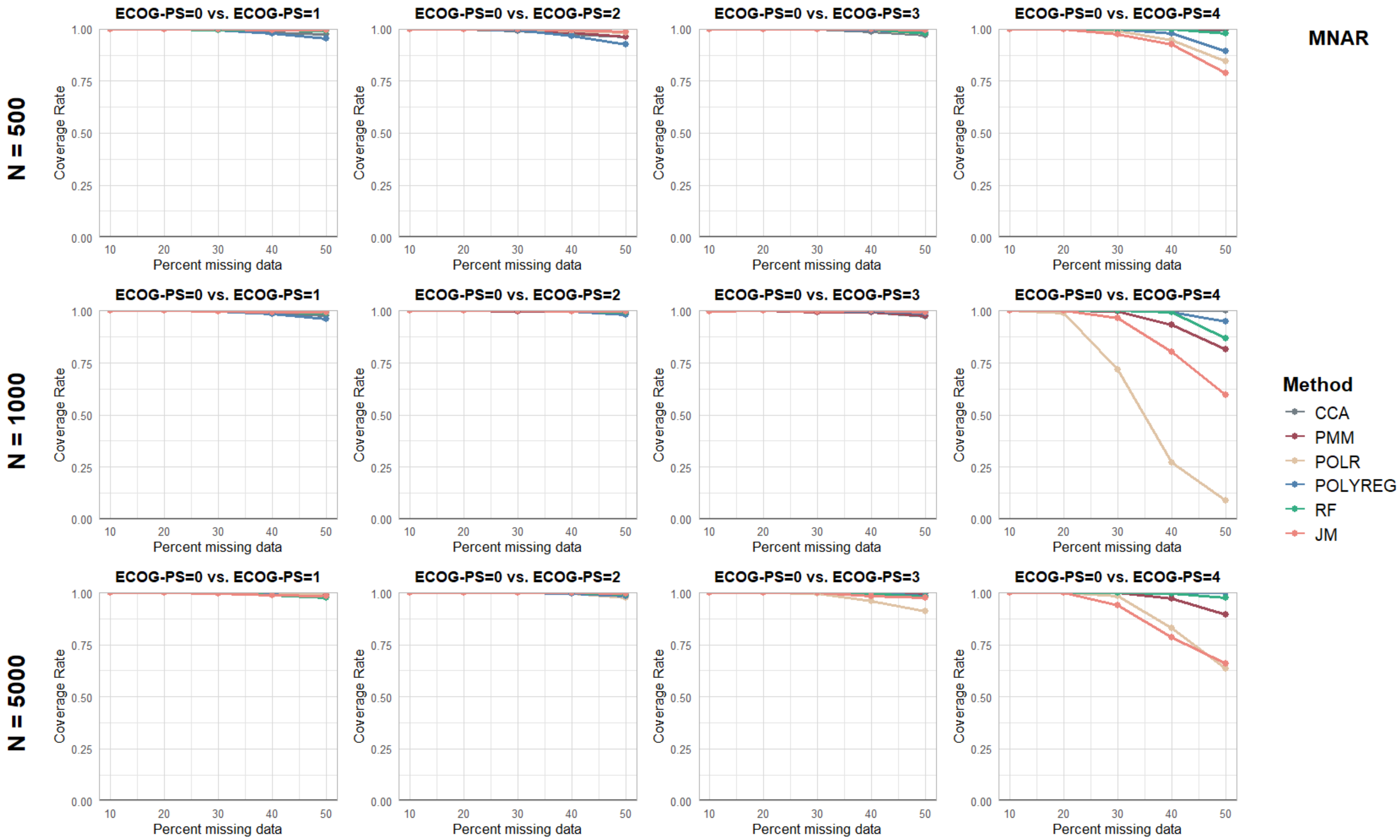

Supplementary Figure S8 – Results of the Performance Parameter ‘Coverage Rate’ (Missingness Mechanism: MNAR)

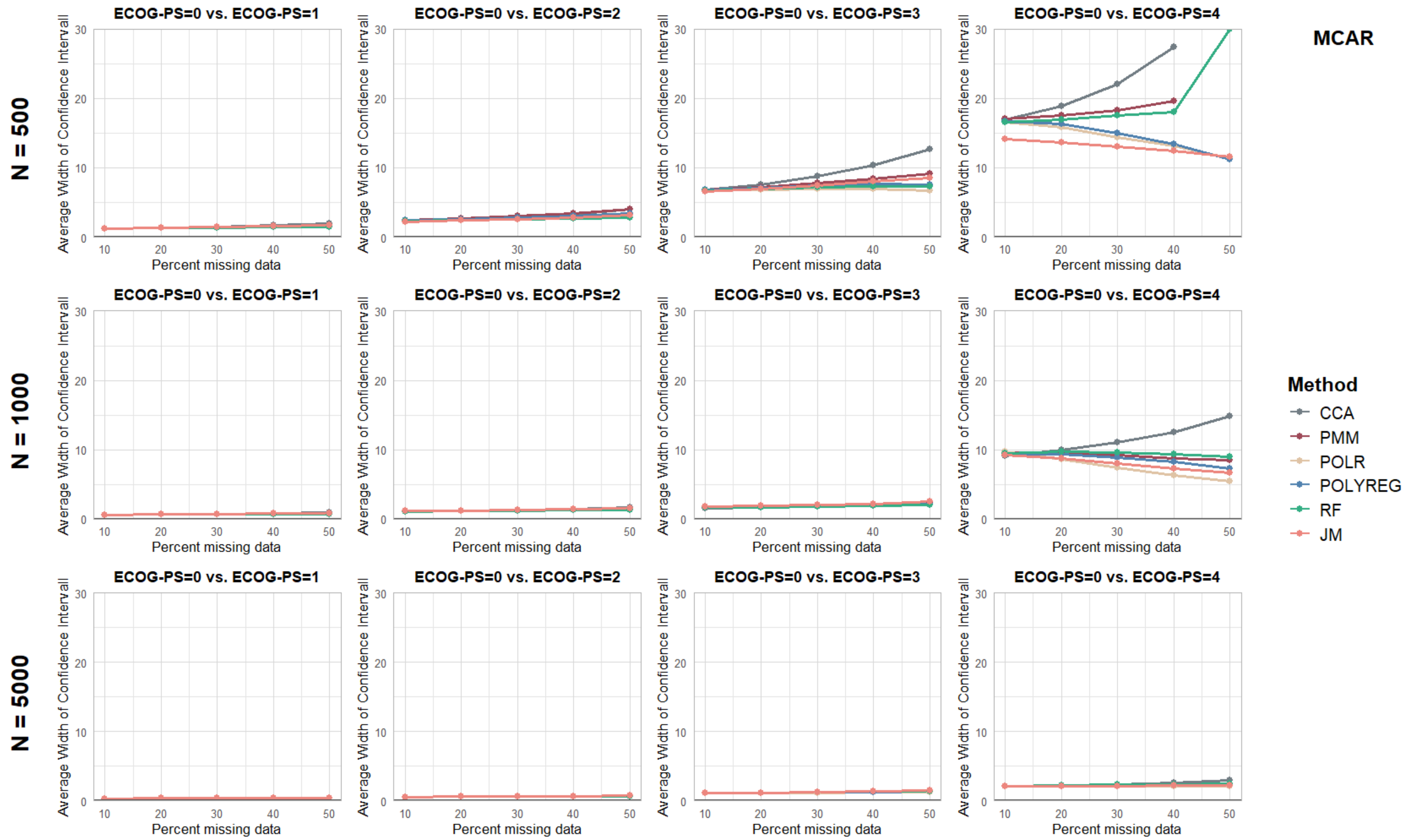

Supplementary Figure S9 – Results of the Performance Parameter 'Average Width of the 95% CI' (Missingness Mechanism: MCAR)

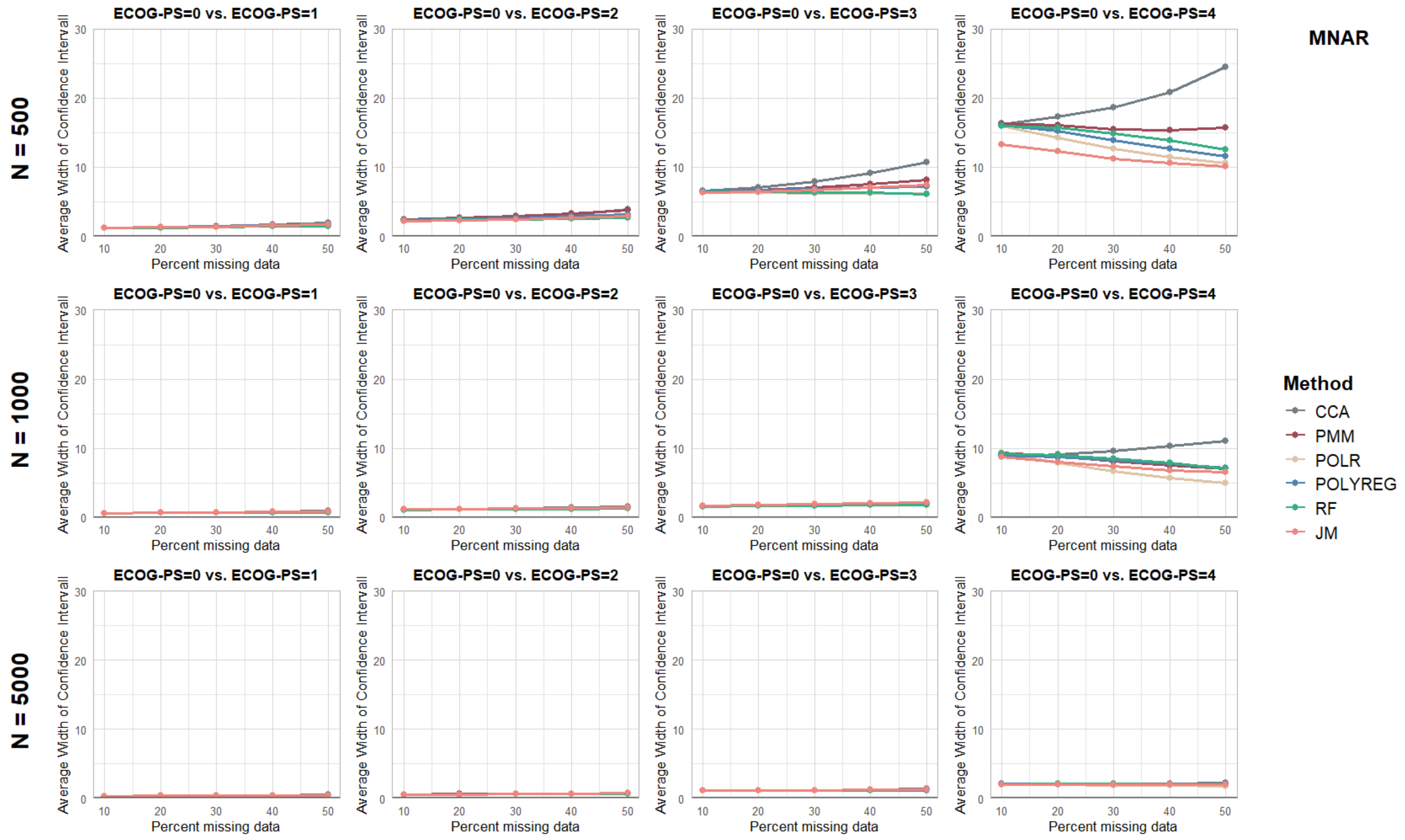

Supplementary Figure S10 – Results of the Performance Parameter 'Average Width of the 95% CI' (Missingness Mechanism: MNAR)

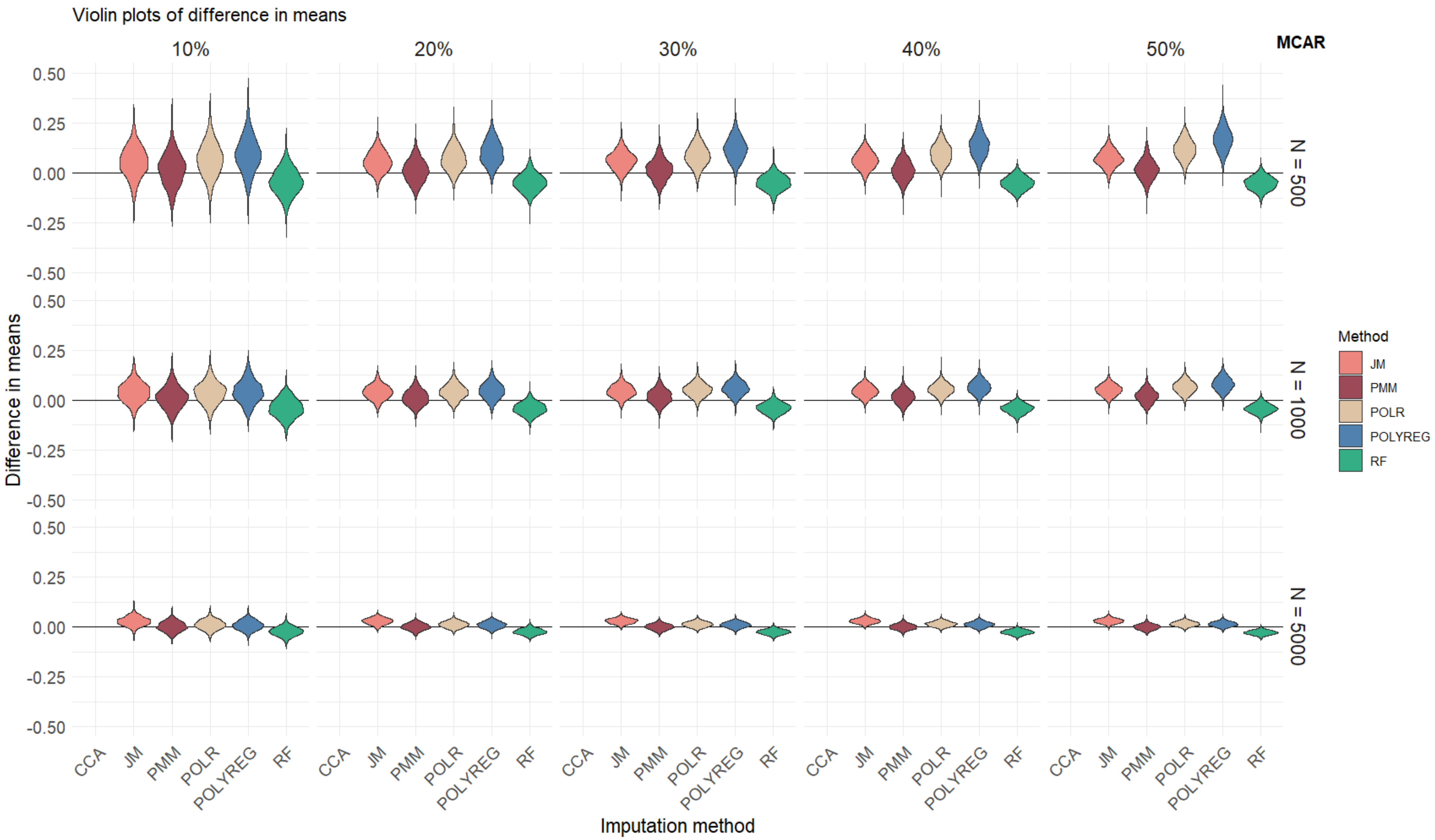

Supplementary Figure S11 – Violin plot of the difference in means between observed and imputed ECOG-PS values (Missingness Mechanism: MCAR)

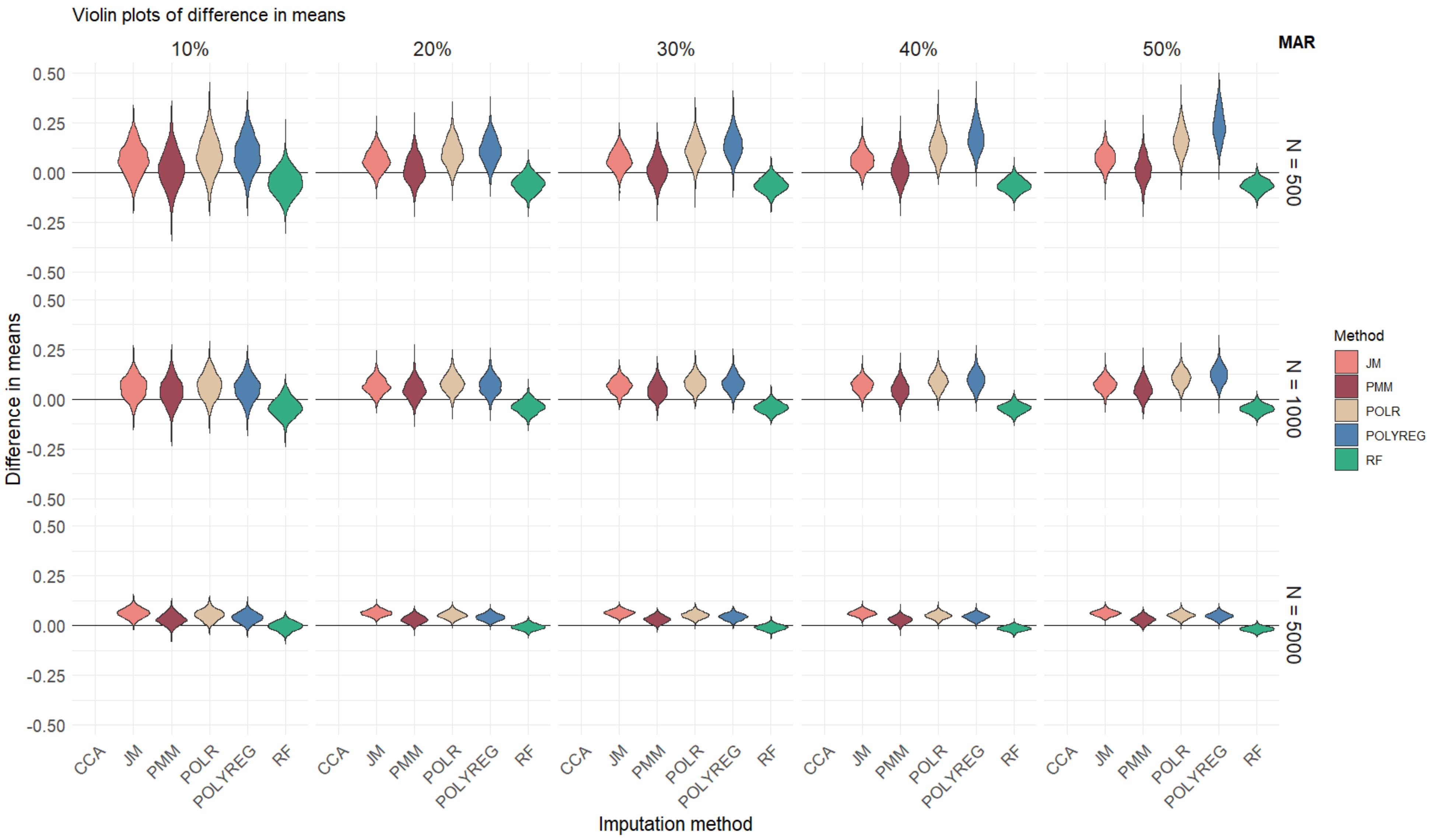

Supplementary Figure S12 - Violin plot of the difference in means between observed and imputed ECOG-PS values (Missingness Mechanism: MAR)

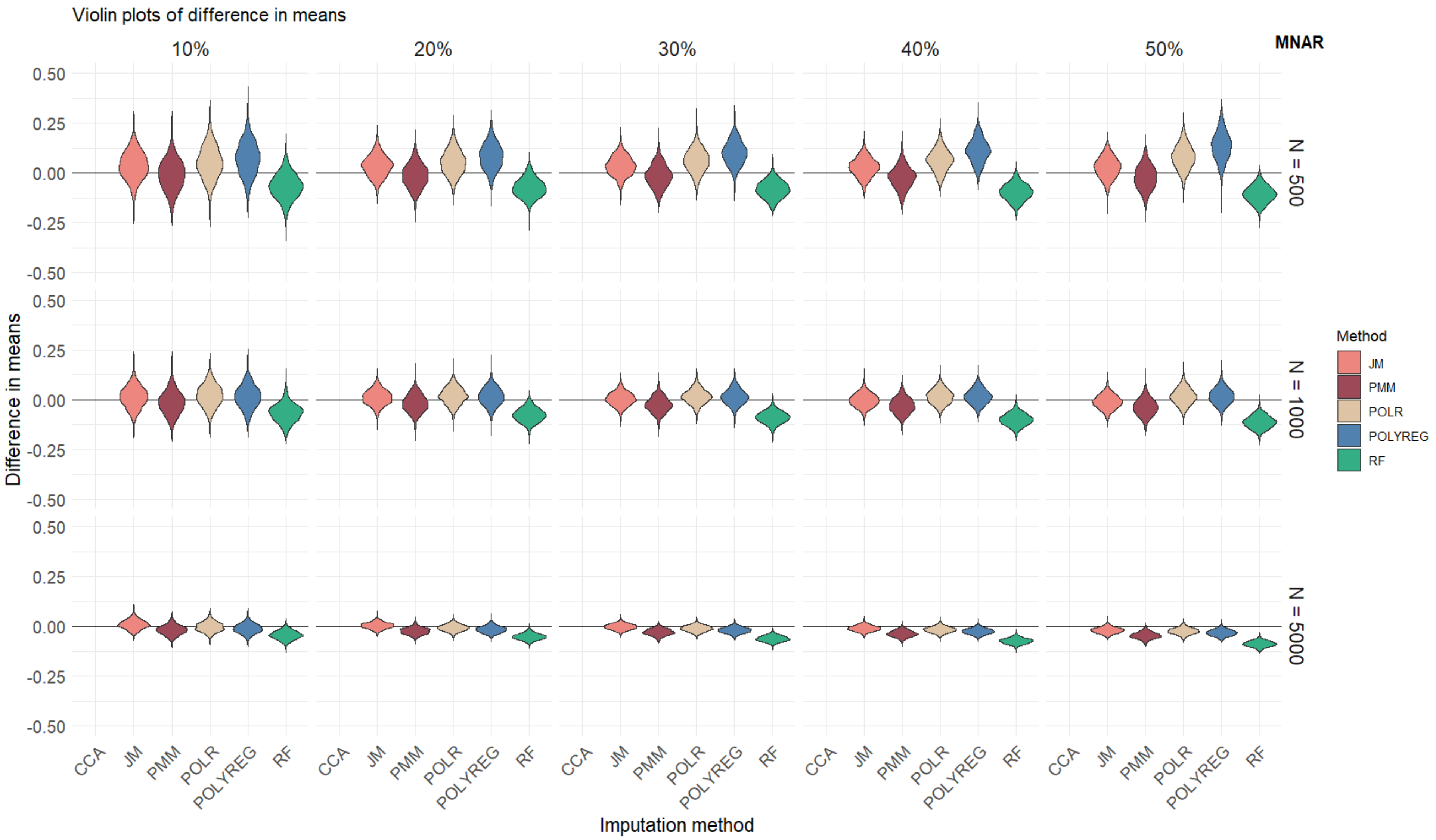

Supplementary Figure S13 - Violin plot of the difference in means between observed and imputed ECOG-PS values (Missingness Mechanism: MNAR)

Supplementary Table S3 – Results of Monte Carlo Simulation with regard to absolute bias, relative bias, mean squared error, coverage rate and average width of 95% confidence interval

Scenario	Miss-mech	Miss-prop	Method	n	Absolute Bias				Relative Bias				Mean Squared Error				Coverage Rate				Average Width of 95%CI			
					0 vs 1	0 vs 2	0 vs 3	0 vs 4	0 vs 1	0 vs 2	0 vs 3	0 vs 4	0 vs 1	0 vs 2	0 vs 3	0 vs 4	0 vs 1	0 vs 2	0 vs 3	0 vs 4	0 vs 1	0 vs 2	0 vs 3	0 vs 4
1	MAR	0.1	CCA	500	0.032	0.049	0.049	-0.008	7.4	5.3	2.9	-0.3	0.006	0.015	0.024	0.012	1.000	1.000	1.000	1.000	1.24	2.44	7.05	16.36
1	MAR	0.1	PMM	500	0.046	0.076	0.022	-0.094	10.6	8.2	1.3	-4.0	0.007	0.019	0.020	0.021	1.000	1.000	1.000	1.000	1.33	2.65	7.31	17.25
1	MAR	0.1	POLR	500	0.054	0.060	-0.014	-0.187	12.3	6.5	-0.9	-8.0	0.008	0.016	0.018	0.051	1.000	1.000	1.000	1.000	1.34	2.61	7.29	16.70
1	MAR	0.1	POLYREG	500	0.045	0.067	0.040	-0.093	10.2	7.3	2.4	-4.0	0.007	0.018	0.023	0.024	1.000	0.999	1.000	1.000	1.33	2.62	7.43	17.31
1	MAR	0.1	RF	500	0.007	0.012	-0.013	-0.090	1.6	1.3	-0.8	-3.9	0.005	0.012	0.021	0.020	1.000	1.000	1.000	1.000	1.27	2.48	7.16	16.63
1	MAR	0.1	JM	500	0.051	-0.012	0.028	-0.289	11.7	-1.3	1.7	-12.4	0.007	0.008	0.021	0.098	1.000	1.000	1.000	1.000	1.28	2.31	7.05	14.82
2	MCAR	0.1	CCA	500	0.003	0.010	0.011	0.013	0.8	1.0	0.6	0.6	0.006	0.011	0.017	0.015	1.000	1.000	1.000	1.000	1.23	2.37	6.83	16.99
2	MCAR	0.1	PMM	500	0.014	0.023	-0.037	-0.092	3.2	2.5	-2.2	-3.9	0.006	0.011	0.016	0.024	1.000	1.000	1.000	1.000	1.27	2.46	6.77	17.09
2	MCAR	0.1	POLR	500	0.018	0.010	-0.067	-0.172	4.0	1.1	-4.0	-7.4	0.005	0.010	0.019	0.046	1.000	1.000	1.000	1.000	1.27	2.42	6.70	16.61
2	MCAR	0.1	POLYREG	500	0.005	0.003	-0.035	-0.125	1.0	0.4	-2.1	-5.4	0.006	0.011	0.019	0.035	1.000	1.000	1.000	1.000	1.26	2.41	6.80	16.72
2	MCAR	0.1	RF	500	-0.008	-0.018	-0.052	-0.093	-1.9	-2.0	-3.1	-4.0	0.005	0.011	0.018	0.022	1.000	1.000	1.000	1.000	1.23	2.35	6.66	16.57
2	MCAR	0.1	JM	500	0.042	-0.031	-0.031	-0.331	9.5	-3.4	-1.9	-14.2	0.007	0.009	0.015	0.126	1.000	1.000	1.000	1.000	1.26	2.25	6.56	14.10
3	MNAR	0.1	CCA	500	0.009	0.016	0.019	0.019	2.0	1.7	1.2	0.8	0.005	0.008	0.008	0.008	1.000	1.000	1.000	1.000	1.22	2.34	6.64	16.26
3	MNAR	0.1	PMM	500	0.015	0.026	-0.031	-0.100	3.5	2.8	-1.9	-4.3	0.005	0.008	0.009	0.020	1.000	1.000	1.000	1.000	1.25	2.42	6.60	16.35
3	MNAR	0.1	POLR	500	0.018	0.013	-0.073	-0.186	4.1	1.4	-4.3	-8.0	0.005	0.008	0.013	0.045	1.000	1.000	1.000	1.000	1.25	2.38	6.49	15.94
3	MNAR	0.1	POLYREG	500	0.008	0.005	-0.030	-0.112	1.7	0.6	-1.8	-4.8	0.006	0.009	0.009	0.024	1.000	1.000	1.000	1.000	1.24	2.37	6.62	16.11
3	MNAR	0.1	RF	500	-0.009	-0.014	-0.050	-0.086	-2.0	-1.6	-3.0	-3.7	0.005	0.007	0.009	0.014	1.000	1.000	1.000	1.000	1.22	2.33	6.49	15.92
3	MNAR	0.1	JM	500	0.043	-0.033	-0.033	-0.331	9.8	-3.5	-2.0	-14.2	0.007	0.007	0.008	0.120	1.000	1.000	1.000	1.000	1.25	2.21	6.37	13.29

4	MAR	0.2	CCA	500	0.062	0.074	0.109	0.008	14.0	8.0	6.5	0.3	0.015	0.028	0.057	0.028	1.000	0.998	0.990	1.000	1.37	2.70	8.16	17.87
4	MAR	0.2	PMM	500	0.083	0.133	0.041	-0.153	18.9	14.4	2.5	-6.6	0.016	0.037	0.035	0.045	1.000	0.999	0.997	1.000	1.49	3.07	8.24	17.78
4	MAR	0.2	POLR	500	0.090	0.090	-0.037	-0.322	20.7	9.7	-2.2	-13.8	0.017	0.027	0.030	0.125	0.999	0.999	0.999	1.000	1.48	2.91	7.78	15.64
4	MAR	0.2	POLYREG	500	0.087	0.108	0.070	-0.202	19.8	11.7	4.2	-8.6	0.019	0.035	0.046	0.070	1.000	0.998	0.995	1.000	1.50	2.97	8.37	17.38
4	MAR	0.2	RF	500	0.004	-0.010	-0.047	-0.184	1.0	-1.1	-2.8	-7.9	0.009	0.017	0.035	0.052	1.000	1.000	0.999	1.000	1.36	2.62	7.72	17.04
4	MAR	0.2	JM	500	0.048	-0.027	0.036	-0.415	10.9	-3.0	2.1	-17.8	0.012	0.015	0.038	0.191	1.000	1.000	0.997	1.000	1.39	2.51	7.90	14.96
5	MCAR	0.2	CCA	500	0.008	0.019	0.023	0.022	1.8	2.1	1.4	1.0	0.013	0.023	0.038	0.034	1.000	0.999	0.996	1.000	1.33	2.59	7.56	18.90
5	MCAR	0.2	PMM	500	0.025	0.041	-0.071	-0.182	5.7	4.5	-4.2	-7.8	0.011	0.021	0.035	0.058	1.000	1.000	1.000	1.000	1.36	2.71	7.17	17.55
5	MCAR	0.2	POLR	500	0.031	0.015	-0.127	-0.330	7.0	1.6	-7.6	-14.1	0.010	0.019	0.043	0.134	1.000	1.000	1.000	1.000	1.36	2.61	6.88	15.81
5	MCAR	0.2	POLYREG	500	0.009	0.006	-0.071	-0.275	2.2	0.7	-4.2	-11.8	0.013	0.023	0.039	0.108	1.000	0.999	1.000	1.000	1.35	2.59	7.12	16.38
5	MCAR	0.2	RF	500	-0.018	-0.042	-0.108	-0.190	-4.1	-4.6	-6.4	-8.1	0.011	0.020	0.039	0.060	1.000	1.000	1.000	1.000	1.30	2.47	6.92	16.99
5	MCAR	0.2	JM	500	0.028	-0.048	-0.072	-0.492	6.4	-5.2	-4.3	-21.1	0.011	0.018	0.034	0.267	1.000	1.000	1.000	0.997	1.34	2.40	6.95	13.65
6	MNAR	0.2	CCA	500	0.016	0.028	0.037	0.040	3.8	3.0	2.2	1.7	0.012	0.017	0.020	0.018	1.000	0.999	1.000	1.000	1.33	2.54	7.13	17.30
6	MNAR	0.2	PMM	500	0.025	0.051	-0.064	-0.182	5.7	5.5	-3.8	-7.8	0.009	0.017	0.019	0.048	1.000	0.999	1.000	1.000	1.33	2.63	6.75	16.11
6	MNAR	0.2	POLR	500	0.030	0.018	-0.133	-0.335	6.9	2.0	-7.9	-14.3	0.009	0.014	0.032	0.127	1.000	1.000	0.999	1.000	1.33	2.52	6.38	14.22
6	MNAR	0.2	POLYREG	500	0.013	0.005	-0.062	-0.226	3.0	0.6	-3.7	-9.7	0.012	0.017	0.021	0.070	1.000	0.999	1.000	1.000	1.33	2.52	6.73	15.20
6	MNAR	0.2	RF	500	-0.022	-0.039	-0.114	-0.186	-5.0	-4.2	-6.8	-8	0.010	0.015	0.026	0.046	1.000	1.000	1.000	1.000	1.28	2.40	6.48	15.67
6	MNAR	0.2	JM	500	0.027	-0.058	-0.082	-0.476	6.2	-6.3	-4.9	-20.4	0.010	0.015	0.021	0.239	1.000	1.000	1.000	0.999	1.32	2.31	6.48	12.35
7	MAR	0.3	CCA	500	0.079	0.083	0.180	0.014	18.1	9.0	10.7	0.6	0.029	0.045	0.111	0.052	0.988	0.985	0.959	1.000	1.50	2.97	9.74	19.81
7	MAR	0.3	PMM	500	0.101	0.167	0.055	-0.217	23.0	18.1	3.3	-9.3	0.025	0.057	0.055	0.081	0.999	0.990	0.999	1.000	1.62	3.45	9.05	17.97
7	MAR	0.3	POLR	500	0.108	0.099	-0.069	-0.450	24.7	10.8	-4.1	-19.3	0.026	0.038	0.047	0.234	0.999	0.998	1.000	0.997	1.60	3.14	8.02	14.12
7	MAR	0.3	POLYREG	500	0.121	0.136	0.090	-0.346	27.6	14.7	5.3	-14.8	0.038	0.057	0.071	0.165	0.986	0.982	0.995	0.998	1.67	3.30	9.18	15.96

7	MAR	0.3	RF	500	-0.015	-0.052	-0.090	-0.296	-3.4	-5.6	-5.3	-12.7	0.017	0.028	0.055	0.116	0.999	0.997	0.998	1.000	1.42	2.70	8.10	16.98
7	MAR	0.3	JM	500	0.031	-0.060	0.040	-0.542	7.0	-6.5	2.4	-23.2	0.018	0.028	0.057	0.325	1.000	0.997	0.997	0.994	1.50	2.69	8.75	14.42
8	MCAR	0.3	CCA	500	0.015	0.045	0.052	0.045	3.5	4.9	3.1	1.9	0.023	0.043	0.073	0.072	0.993	0.987	0.991	0.999	1.46	2.93	8.72	22.06
8	MCAR	0.3	PMM	500	0.041	0.079	-0.086	-0.250	9.4	8.5	-5.1	-10.7	0.017	0.038	0.056	0.102	0.999	0.992	0.998	0.999	1.47	3.06	7.75	18.27
8	MCAR	0.3	POLR	500	0.042	0.032	-0.181	-0.473	9.6	3.4	-10.8	-20.3	0.017	0.032	0.073	0.258	0.999	0.997	0.999	0.994	1.46	2.83	6.98	14.36
8	MCAR	0.3	POLYREG	500	0.020	0.031	-0.097	-0.425	4.5	3.4	-5.8	-18.2	0.024	0.041	0.066	0.232	0.995	0.984	0.996	0.990	1.46	2.87	7.49	15.01
8	MCAR	0.3	RF	500	-0.028	-0.055	-0.160	-0.285	-6.4	-6.0	-9.6	-12.2	0.019	0.033	0.070	0.117	0.997	0.997	0.999	1.000	1.38	2.63	7.19	17.53
8	MCAR	0.3	JM	500	0.015	-0.054	-0.109	-0.644	3.4	-5.8	-6.5	-27.6	0.019	0.030	0.059	0.449	0.997	0.996	0.999	0.975	1.44	2.61	7.49	13.00
9	MNAR	0.3	CCA	500	0.026	0.046	0.070	0.066	6.0	5.0	4.2	2.8	0.022	0.031	0.036	0.034	0.996	0.993	0.999	1.000	1.46	2.80	7.87	18.68
9	MNAR	0.3	PMM	500	0.037	0.082	-0.074	-0.248	8.5	8.8	-4.4	-10.6	0.013	0.029	0.028	0.084	1.000	0.996	1.000	1.000	1.42	2.89	7.02	15.46
9	MNAR	0.3	POLR	500	0.044	0.032	-0.173	-0.439	10.0	3.4	-10.3	-18.8	0.013	0.023	0.051	0.214	1.000	1.000	0.999	0.992	1.41	2.66	6.24	12.68
9	MNAR	0.3	POLYREG	500	0.022	0.016	-0.077	-0.311	5.1	1.7	-4.6	-13.3	0.021	0.032	0.033	0.124	0.995	0.994	1.000	1.000	1.43	2.69	6.82	13.87
9	MNAR	0.3	RF	500	-0.030	-0.057	-0.166	-0.281	-6.8	-6.2	-9.9	-12	0.015	0.024	0.046	0.094	0.997	0.998	1.000	1.000	1.33	2.47	6.40	14.89
9	MNAR	0.3	JM	500	0.011	-0.079	-0.120	-0.586	2.6	-8.5	-7.1	-25.1	0.015	0.026	0.037	0.358	1.000	0.998	1.000	0.975	1.40	2.43	6.68	11.27
10	MAR	0.4	CCA	500	0.103	0.089	0.236	0.043	23.5	9.7	14.0	1.8	0.048	0.065	0.177	0.082	0.973	0.978	0.926	0.997	1.70	3.34	11.77	22.97
10	MAR	0.4	PMM	500	0.123	0.200	0.056	-0.274	28.1	21.7	3.3	-11.7	0.035	0.079	0.074	0.121	0.994	0.975	1.000	1.000	1.78	3.93	9.98	18.36
10	MAR	0.4	POLR	500	0.121	0.097	-0.129	-0.555	27.7	10.5	-7.6	-23.8	0.035	0.048	0.071	0.352	0.995	0.996	0.999	0.958	1.71	3.31	7.91	12.68
10	MAR	0.4	POLYREG	500	0.162	0.152	0.073	-0.507	36.9	16.5	4.4	-21.7	0.066	0.080	0.094	0.324	0.953	0.958	0.984	0.954	1.85	3.60	9.52	13.71
10	MAR	0.4	RF	500	-0.038	-0.112	-0.174	-0.424	-8.7	-12.1	-10.3	-18.2	0.024	0.044	0.089	0.214	0.998	0.996	0.992	1.000	1.46	2.71	8.12	16.39
10	MAR	0.4	JM	500	0.013	-0.099	0.017	-0.648	3.0	-10.8	1.0	-27.8	0.027	0.047	0.074	0.459	0.997	0.992	0.998	0.980	1.64	2.89	9.57	13.90
11	MCAR	0.4	CCA	500	0.031	0.065	0.087	0.096	7.1	7.0	5.2	4.1	0.039	0.067	0.129	0.132	0.993	0.977	0.969	0.991	1.65	3.35	10.41	27.46
11	MCAR	0.4	PMM	500	0.060	0.102	-0.101	-0.296	13.8	11.1	-6.0	-12.7	0.025	0.055	0.083	0.153	0.997	0.988	0.993	0.999	1.61	3.44	8.42	19.57

11	MCAR	0.4	POLR	500	0.050	0.030	-0.235	-0.591	11.5	3.2	-14.0	-25.3	0.023	0.043	0.112	0.399	1.000	0.992	0.981	0.943	1.54	3.00	6.98	13.11
11	MCAR	0.4	POLYREG	500	0.030	0.039	-0.134	-0.572	6.9	4.3	-8.0	-24.5	0.036	0.061	0.107	0.402	0.987	0.977	0.982	0.929	1.58	3.11	7.73	13.46
11	MCAR	0.4	RF	500	-0.043	-0.089	-0.227	-0.386	-9.7	-9.7	-13.5	-16.5	0.026	0.046	0.111	0.201	0.994	0.992	0.983	0.997	1.44	2.70	7.37	18.01
11	MCAR	0.4	JM	500	0.004	-0.071	-0.155	-0.777	0.9	-7.7	-9.3	-33.3	0.027	0.044	0.093	0.652	0.999	0.995	0.993	0.913	1.58	2.85	8.06	12.39
12	MNAR	0.4	CCA	500	0.053	0.083	0.120	0.100	12.0	9.0	7.2	4.3	0.041	0.057	0.071	0.065	0.984	0.975	0.988	1.000	1.68	3.23	9.10	20.90
12	MNAR	0.4	PMM	500	0.063	0.133	-0.061	-0.277	14.4	14.4	-3.6	-11.9	0.022	0.051	0.040	0.112	0.998	0.984	0.999	1.000	1.55	3.27	7.55	15.36
12	MNAR	0.4	POLR	500	0.068	0.056	-0.197	-0.509	15.5	6.1	-11.7	-21.8	0.022	0.035	0.071	0.292	0.998	0.993	0.994	0.948	1.52	2.86	6.23	11.47
12	MNAR	0.4	POLYREG	500	0.049	0.045	-0.076	-0.382	11.2	4.9	-4.5	-16.4	0.039	0.056	0.052	0.189	0.978	0.968	0.995	0.978	1.57	2.95	7.04	12.68
12	MNAR	0.4	RF	500	-0.028	-0.066	-0.215	-0.382	-6.5	-7.2	-12.8	-16.4	0.023	0.035	0.074	0.167	0.995	0.995	0.999	1.000	1.41	2.58	6.38	13.86
12	MNAR	0.4	JM	500	0.008	-0.083	-0.140	-0.667	1.9	-9.0	-8.3	-28.6	0.024	0.039	0.053	0.466	0.996	0.994	1.000	0.928	1.54	2.66	7.06	10.63
13	MAR	0.5	CCA	500	0.120	0.099	0.303	0.100	27.3	10.8	18.0	4.3	0.074	0.100	0.267	0.139	0.952	0.952	0.900	0.996	1.96	3.88	14.88	28.66
13	MAR	0.5	PMM	500	0.143	0.243	0.074	-0.309	32.6	26.3	4.4	-13.3	0.049	0.116	0.100	0.169	0.984	0.949	0.990	1.000	1.94	4.53	11.25	19.24
13	MAR	0.5	POLR	500	0.130	0.098	-0.184	-0.643	29.6	10.6	-10.9	-27.5	0.043	0.061	0.103	0.478	0.992	0.987	0.980	0.834	1.81	3.47	7.72	11.30
13	MAR	0.5	POLYREG	500	0.187	0.169	0.052	-0.654	42.6	18.3	3.1	-28.0	0.096	0.120	0.137	0.525	0.901	0.924	0.961	0.797	2.02	3.92	9.72	11.43
13	MAR	0.5	RF	500	-0.070	-0.173	-0.268	-0.565	-16.0	-18.7	-16.0	-24.2	0.033	0.071	0.140	0.363	0.989	0.975	0.979	0.989	1.48	2.68	8.02	15.55
13	MAR	0.5	JM	500	-0.011	-0.131	-0.016	-0.762	-2.5	-14.2	-1.0	-32.7	0.038	0.076	0.097	0.629	0.997	0.979	0.995	0.941	1.80	3.17	10.42	13.18
14	MCAR	0.5	CCA	500	0.047	0.094	0.105	NA	10.8	10.2	6.3	NA	0.064	0.103	0.191	NA	0.974	0.954	0.958	NA	1.93	4.02	12.63	NA
14	MCAR	0.5	PMM	500	0.077	0.130	-0.129	-0.360	17.5	14.1	-7.7	-15.4	0.033	0.076	0.115	0.224	0.997	0.975	0.987	0.994	1.77	3.99	9.20	Inf
14	MCAR	0.5	POLR	500	0.055	0.025	-0.309	-0.714	12.5	2.8	-18.4	-30.6	0.029	0.055	0.167	0.578	0.997	0.980	0.954	0.806	1.63	3.17	6.75	11.39
14	MCAR	0.5	POLYREG	500	0.043	0.057	-0.205	-0.749	9.9	6.2	-12.2	-32.1	0.060	0.090	0.175	0.664	0.952	0.941	0.938	0.745	1.71	3.38	7.55	11.22
14	MCAR	0.5	RF	500	-0.062	-0.129	-0.331	-0.517	-14.1	-14.0	-19.7	-22.1	0.037	0.066	0.184	0.345	0.986	0.974	0.955	0.983	1.51	2.79	7.28	Inf
14	MCAR	0.5	JM	500	-0.003	-0.080	-0.222	-0.918	-0.8	-8.6	-13.2	-39.3	0.042	0.065	0.149	0.902	0.992	0.982	0.981	0.790	1.76	3.20	8.49	11.58

15	MNAR	0.5	CCA	500	0.082	0.122	0.164	0.150	18.7	13.2	9.7	6.4	0.067	0.095	0.114	0.111	0.973	0.961	0.972	0.996	2.00	3.88	10.73	24.47
15	MNAR	0.5	PMM	500	0.090	0.179	-0.040	-0.281	20.6	19.4	-2.4	-12.1	0.032	0.079	0.059	0.133	0.993	0.964	0.997	0.998	1.70	3.75	8.23	15.70
15	MNAR	0.5	POLR	500	0.091	0.073	-0.216	-0.551	20.7	7.9	-12.8	-23.6	0.031	0.049	0.093	0.351	0.991	0.983	0.977	0.846	1.63	3.04	6.16	10.57
15	MNAR	0.5	POLYREG	500	0.070	0.066	-0.068	-0.433	16.0	7.1	-4.0	-18.6	0.063	0.094	0.081	0.254	0.953	0.927	0.981	0.892	1.71	3.21	7.21	11.56
15	MNAR	0.5	RF	500	-0.030	-0.091	-0.281	-0.493	-6.9	-9.8	-16.7	-21.1	0.030	0.047	0.114	0.269	0.992	0.988	0.979	0.980	1.48	2.63	6.15	12.56
15	MNAR	0.5	JM	500	0.000	-0.099	-0.174	-0.733	-0.1	-10.8	-10.3	-31.4	0.036	0.058	0.082	0.566	0.994	0.987	0.996	0.787	1.71	2.93	7.40	10.07
16	MAR	0.1	CCA	1000	0.022	0.034	-0.001	-0.005	8.9	5.0	-0.1	-0.2	0.002	0.004	0.005	0.005	1.000	1.000	0.999	1.000	0.62	1.14	1.59	8.91
16	MAR	0.1	PMM	1000	0.027	0.038	0.024	-0.157	11.2	5.7	3.0	-7.2	0.002	0.005	0.005	0.032	1.000	1.000	0.999	1.000	0.65	1.19	1.69	9.78
16	MAR	0.1	POLR	1000	0.026	0.030	-0.005	-0.262	10.8	4.5	-0.7	-12	0.002	0.004	0.005	0.078	1.000	1.000	1.000	0.999	0.64	1.18	1.64	9.80
16	MAR	0.1	POLYREG	1000	0.021	0.029	0.010	-0.080	8.5	4.3	1.3	-3.7	0.002	0.004	0.005	0.013	1.000	1.000	1.000	1.000	0.64	1.17	1.67	9.30
16	MAR	0.1	RF	1000	0.003	0.012	-0.016	-0.122	1.1	1.8	-2.0	-5.6	0.002	0.003	0.005	0.021	1.000	1.000	0.999	1.000	0.63	1.16	1.63	9.57
16	MAR	0.1	JM	1000	0.058	0.062	0.085	-0.236	23.7	9.2	10.5	-10.8	0.005	0.007	0.012	0.064	1.000	1.000	0.999	1.000	0.66	1.20	1.77	9.53
17	MCAR	0.1	CCA	1000	0.003	0.005	0.006	0.008	1.2	0.8	0.8	0.4	0.002	0.003	0.006	0.006	1.000	1.000	1.000	1.000	0.62	1.12	1.60	9.14
17	MCAR	0.1	PMM	1000	0.009	0.003	0.017	-0.136	3.8	0.4	2.2	-6.3	0.002	0.003	0.006	0.026	1.000	1.000	1.000	1.000	0.62	1.14	1.65	9.64
17	MCAR	0.1	POLR	1000	0.010	0.000	-0.006	-0.225	4.3	0.0	-0.7	-10.4	0.002	0.003	0.005	0.060	1.000	1.000	1.000	1.000	0.63	1.13	1.62	9.67
17	MCAR	0.1	POLYREG	1000	-0.002	-0.004	0.002	-0.086	-0.7	-0.6	0.2	-3.9	0.002	0.003	0.006	0.014	1.000	1.000	1.000	1.000	0.62	1.12	1.64	9.27
17	MCAR	0.1	RF	1000	-0.001	-0.011	-0.005	-0.100	-0.4	-1.6	-0.6	-4.6	0.002	0.003	0.006	0.017	1.000	1.000	1.000	1.000	0.62	1.12	1.62	9.48
17	MCAR	0.1	JM	1000	0.038	0.035	0.078	-0.223	15.3	5.2	9.7	-10.3	0.003	0.004	0.011	0.058	1.000	1.000	1.000	1.000	0.63	1.14	1.74	9.22
18	MNAR	0.1	CCA	1000	0.005	0.005	0.016	0.011	2.1	0.7	2.0	0.5	0.002	0.002	0.003	0.002	1.000	1.000	1.000	1.000	0.61	1.10	1.57	8.78
18	MNAR	0.1	PMM	1000	0.011	0.004	0.024	-0.141	4.3	0.5	3.0	-6.5	0.001	0.002	0.003	0.025	1.000	1.000	1.000	1.000	0.62	1.12	1.61	9.34
18	MNAR	0.1	POLR	1000	0.012	0.000	0.002	-0.237	4.8	0.0	0.2	-10.9	0.002	0.002	0.003	0.063	1.000	1.000	1.000	1.000	0.62	1.11	1.58	9.35
18	MNAR	0.1	POLYREG	1000	0.001	-0.004	0.009	-0.074	0.3	-0.6	1.2	-3.4	0.002	0.002	0.003	0.009	1.000	1.000	1.000	1.000	0.62	1.11	1.60	8.94

18	MNAR	0.1	RF	1000	-0.001	-0.011	0.001	-0.093	-0.5	-1.7	0.2	-4.3	0.001	0.002	0.003	0.013	1.000	1.000	1.000	1.000	0.61	1.10	1.58	9.19
18	MNAR	0.1	JM	1000	0.037	0.033	0.083	-0.216	15.3	5.0	10.3	-9.9	0.003	0.003	0.009	0.052	1.000	1.000	0.999	1.000	0.63	1.13	1.70	8.76
19	MAR	0.2	CCA	1000	0.039	0.065	-0.004	-0.008	15.8	9.6	-0.6	-0.4	0.005	0.010	0.010	0.011	0.999	0.997	1.000	1.000	0.68	1.26	1.70	9.53
19	MAR	0.2	PMM	1000	0.050	0.071	0.048	-0.276	20.2	10.6	6.0	-12.7	0.005	0.010	0.010	0.088	1.000	0.999	1.000	0.999	0.70	1.33	1.86	9.43
19	MAR	0.2	POLR	1000	0.050	0.056	0.001	-0.468	20.4	8.3	0.1	-21.5	0.005	0.009	0.007	0.230	1.000	0.999	1.000	0.975	0.70	1.29	1.76	8.45
19	MAR	0.2	POLYREG	1000	0.036	0.055	0.020	-0.187	14.7	8.2	2.4	-8.6	0.005	0.010	0.010	0.046	1.000	0.999	1.000	1.000	0.69	1.29	1.81	9.27
19	MAR	0.2	RF	1000	0.004	0.018	-0.036	-0.244	1.6	2.6	-4.4	-11.2	0.003	0.006	0.010	0.069	1.000	0.999	1.000	1.000	0.67	1.24	1.71	9.69
19	MAR	0.2	JM	1000	0.068	0.076	0.084	-0.404	27.8	11.3	10.4	-18.6	0.008	0.012	0.015	0.174	0.999	0.996	1.000	0.997	0.71	1.31	1.93	8.96
20	MCAR	0.2	CCA	1000	0.005	0.012	0.011	0.017	1.8	1.7	1.3	0.8	0.004	0.006	0.012	0.015	1.000	1.000	0.996	1.000	0.66	1.20	1.73	9.94
20	MCAR	0.2	PMM	1000	0.016	0.007	0.033	-0.261	6.4	1.0	4.0	-12.0	0.003	0.005	0.011	0.082	1.000	1.000	0.999	1.000	0.66	1.21	1.79	9.52
20	MCAR	0.2	POLR	1000	0.019	-0.001	-0.010	-0.429	7.7	-0.1	-1.3	-19.7	0.004	0.005	0.010	0.196	1.000	1.000	1.000	0.990	0.66	1.20	1.71	8.66
20	MCAR	0.2	POLYREG	1000	-0.006	-0.006	-0.001	-0.186	-2.3	-0.9	-0.1	-8.6	0.004	0.006	0.011	0.047	1.000	1.000	0.997	1.000	0.65	1.18	1.74	9.30
20	MCAR	0.2	RF	1000	-0.007	-0.024	-0.015	-0.208	-3.0	-3.5	-1.9	-9.5	0.004	0.006	0.011	0.055	1.000	1.000	0.999	1.000	0.65	1.17	1.72	9.66
20	MCAR	0.2	JM	1000	0.029	0.024	0.067	-0.395	12.0	3.6	8.3	-18.1	0.004	0.006	0.014	0.167	1.000	1.000	0.999	0.995	0.67	1.21	1.87	8.72
21	MNAR	0.2	CCA	1000	0.012	0.017	0.032	0.026	4.8	2.5	3.9	1.2	0.004	0.005	0.007	0.006	1.000	1.000	1.000	1.000	0.66	1.18	1.66	9.16
21	MNAR	0.2	PMM	1000	0.022	0.014	0.051	-0.255	8.9	2.0	6.3	-11.7	0.003	0.004	0.007	0.072	1.000	1.000	1.000	1.000	0.66	1.19	1.71	8.84
21	MNAR	0.2	POLR	1000	0.026	0.006	0.007	-0.418	10.7	0.9	0.9	-19.2	0.003	0.004	0.005	0.182	1.000	1.000	1.000	0.989	0.66	1.17	1.64	7.92
21	MNAR	0.2	POLYREG	1000	0.003	0.000	0.018	-0.140	1.4	0.1	2.2	-6.4	0.004	0.005	0.006	0.025	1.000	1.000	1.000	1.000	0.65	1.16	1.67	8.69
21	MNAR	0.2	RF	1000	-0.002	-0.019	0.002	-0.190	-0.7	-2.8	0.2	-8.7	0.003	0.004	0.005	0.041	1.000	1.000	1.000	1.000	0.64	1.14	1.64	9.03
21	MNAR	0.2	JM	1000	0.033	0.028	0.079	-0.357	13.4	4.2	9.8	-16.4	0.004	0.005	0.011	0.133	1.000	1.000	1.000	1.000	0.67	1.19	1.77	8.04
22	MAR	0.3	CCA	1000	0.053	0.081	-0.001	0.004	21.6	12.1	-0.2	0.2	0.009	0.016	0.019	0.020	0.999	0.994	0.997	0.999	0.74	1.37	1.85	10.54
22	MAR	0.3	PMM	1000	0.069	0.092	0.078	-0.355	28.0	13.7	9.7	-16.3	0.009	0.016	0.019	0.144	1.000	0.999	0.998	0.999	0.75	1.45	2.06	9.20

22	MAR	0.3	POLR	1000	0.069	0.066	0.013	-0.623	28.0	9.8	1.7	-28.6	0.009	0.012	0.013	0.402	0.999	0.997	0.999	0.714	0.75	1.37	1.88	7.22
22	MAR	0.3	POLYREG	1000	0.046	0.068	0.030	-0.306	18.9	10.1	3.8	-14.1	0.009	0.014	0.017	0.111	0.998	0.994	0.998	0.997	0.74	1.37	1.95	8.87
22	MAR	0.3	RF	1000	0.005	0.011	-0.048	-0.358	1.9	1.6	-6.0	-16.4	0.005	0.008	0.016	0.140	1.000	1.000	1.000	1.000	0.70	1.31	1.82	9.53
22	MAR	0.3	JM	1000	0.073	0.080	0.088	-0.551	29.7	11.9	10.9	-25.3	0.011	0.014	0.021	0.317	0.997	0.997	0.999	0.931	0.78	1.42	2.12	8.30
23	MCAR	0.3	CCA	1000	0.009	0.015	0.019	0.026	3.5	2.2	2.4	1.2	0.007	0.010	0.022	0.026	0.998	1.000	0.988	0.999	0.71	1.31	1.90	11.02
23	MCAR	0.3	PMM	1000	0.026	0.008	0.054	-0.380	10.4	1.2	6.7	-17.5	0.005	0.008	0.018	0.162	1.000	1.000	0.998	0.993	0.71	1.30	1.97	9.18
23	MCAR	0.3	POLR	1000	0.028	-0.006	-0.011	-0.609	11.3	-0.9	-1.4	-28.0	0.005	0.008	0.015	0.386	1.000	1.000	0.999	0.747	0.70	1.26	1.81	7.40
23	MCAR	0.3	POLYREG	1000	-0.006	-0.011	0.003	-0.307	-2.4	-1.6	0.3	-14.1	0.006	0.009	0.019	0.113	1.000	1.000	0.994	0.991	0.69	1.25	1.87	8.90
23	MCAR	0.3	RF	1000	-0.009	-0.038	-0.020	-0.320	-3.9	-5.7	-2.4	-14.7	0.005	0.009	0.017	0.118	0.999	1.000	0.998	1.000	0.68	1.22	1.83	9.57
23	MCAR	0.3	JM	1000	0.026	0.013	0.062	-0.551	10.5	1.9	7.7	-25.3	0.006	0.008	0.020	0.318	1.000	1.000	0.997	0.896	0.72	1.29	2.04	7.97
24	MNAR	0.3	CCA	1000	0.017	0.022	0.051	0.044	7.1	3.3	6.3	2.0	0.007	0.008	0.012	0.010	0.997	0.999	0.996	1.000	0.71	1.26	1.78	9.64
24	MNAR	0.3	PMM	1000	0.033	0.020	0.083	-0.349	13.5	2.9	10.3	-16.0	0.005	0.006	0.014	0.131	1.000	0.999	0.995	0.996	0.70	1.26	1.84	8.07
24	MNAR	0.3	POLR	1000	0.038	0.005	0.018	-0.554	15.5	0.8	2.2	-25.5	0.005	0.006	0.008	0.316	0.999	1.000	1.000	0.719	0.70	1.22	1.70	6.66
24	MNAR	0.3	POLYREG	1000	0.003	-0.001	0.027	-0.201	1.3	-0.2	3.3	-9.2	0.007	0.008	0.009	0.048	0.999	1.000	1.000	1.000	0.69	1.21	1.73	8.27
24	MNAR	0.3	RF	1000	-0.004	-0.033	-0.001	-0.284	-1.5	-4.9	-0.2	-13.1	0.005	0.007	0.007	0.087	0.999	1.000	1.000	1.000	0.67	1.17	1.69	8.55
24	MNAR	0.3	JM	1000	0.026	0.016	0.075	-0.457	10.7	2.3	9.4	-21.0	0.006	0.007	0.013	0.215	0.998	1.000	0.999	0.967	0.72	1.25	1.88	7.36
25	MAR	0.4	CCA	1000	0.064	0.094	0.013	-0.006	26.1	14.0	1.6	-0.3	0.015	0.023	0.032	0.033	0.987	0.984	0.985	1.000	0.81	1.51	2.07	11.51
25	MAR	0.4	PMM	1000	0.090	0.114	0.125	-0.427	36.7	16.9	15.4	-19.6	0.014	0.024	0.036	0.206	0.991	0.990	0.981	0.968	0.81	1.60	2.33	8.87
25	MAR	0.4	POLR	1000	0.088	0.076	0.036	-0.751	35.9	11.3	4.5	-34.5	0.014	0.017	0.019	0.583	0.996	0.994	0.999	0.310	0.80	1.46	2.02	6.18
25	MAR	0.4	POLYREG	1000	0.057	0.080	0.056	-0.454	23.4	11.9	6.9	-20.9	0.015	0.021	0.028	0.232	0.986	0.983	0.988	0.888	0.79	1.46	2.11	7.91
25	MAR	0.4	RF	1000	0.007	0.000	-0.051	-0.487	2.7	0.1	-6.3	-22.4	0.008	0.010	0.023	0.251	0.998	1.000	0.999	0.972	0.74	1.36	1.95	8.98
25	MAR	0.4	JM	1000	0.074	0.082	0.102	-0.686	30.2	12.2	12.7	-31.5	0.015	0.019	0.032	0.488	0.993	0.992	0.992	0.727	0.85	1.54	2.38	7.58

26	MCAR	0.4	CCA	1000	0.015	0.026	0.035	0.046	6.3	3.9	4.4	2.1	0.012	0.017	0.035	0.042	0.994	0.994	0.981	0.998	0.79	1.45	2.12	12.46
26	MCAR	0.4	PMM	1000	0.034	0.014	0.078	-0.468	14.0	2.0	9.6	-21.5	0.007	0.012	0.028	0.247	1.000	1.000	0.987	0.935	0.75	1.41	2.18	8.76
26	MCAR	0.4	POLR	1000	0.038	-0.007	-0.009	-0.753	15.6	-1.0	-1.1	-34.6	0.008	0.012	0.020	0.587	0.999	0.997	0.997	0.357	0.74	1.33	1.90	6.35
26	MCAR	0.4	POLYREG	1000	-0.006	-0.012	0.005	-0.425	-2.3	-1.8	0.7	-19.5	0.011	0.016	0.029	0.208	0.992	0.990	0.982	0.928	0.73	1.31	1.97	8.21
26	MCAR	0.4	RF	1000	-0.012	-0.052	-0.027	-0.426	-4.8	-7.7	-3.4	-19.6	0.008	0.015	0.023	0.202	0.997	0.999	0.996	0.977	0.71	1.27	1.94	9.40
26	MCAR	0.4	JM	1000	0.021	0.005	0.059	-0.686	8.4	0.8	7.3	-31.5	0.009	0.013	0.027	0.488	0.995	0.997	0.990	0.655	0.78	1.40	2.22	7.30
27	MNAR	0.4	CCA	1000	0.029	0.034	0.080	0.064	12.0	5.0	10.0	2.9	0.013	0.015	0.022	0.017	0.989	0.996	0.994	1.000	0.79	1.39	1.96	10.30
27	MNAR	0.4	PMM	1000	0.047	0.031	0.121	-0.422	19.3	4.6	15.0	-19.4	0.008	0.010	0.025	0.193	0.998	0.999	0.995	0.932	0.75	1.35	2.01	7.49
27	MNAR	0.4	POLR	1000	0.056	0.010	0.039	-0.664	22.8	1.5	4.8	-30.5	0.009	0.009	0.012	0.453	0.997	1.000	1.000	0.269	0.74	1.27	1.76	5.69
27	MNAR	0.4	POLYREG	1000	0.013	0.001	0.043	-0.258	5.1	0.2	5.4	-11.8	0.013	0.013	0.015	0.078	0.984	0.997	0.999	0.993	0.73	1.26	1.81	7.76
27	MNAR	0.4	RF	1000	0.000	-0.045	0.005	-0.391	0.1	-6.7	0.6	-18.0	0.008	0.011	0.010	0.161	0.994	0.999	1.000	0.993	0.71	1.21	1.76	7.84
27	MNAR	0.4	JM	1000	0.025	0.009	0.078	-0.536	10.4	1.4	9.6	-24.6	0.010	0.010	0.017	0.295	0.995	0.998	1.000	0.802	0.78	1.34	1.99	6.83
28	MAR	0.5	CCA	1000	0.073	0.101	0.018	0.001	29.9	15.0	2.2	0.0	0.022	0.035	0.053	0.059	0.969	0.959	0.959	0.998	0.91	1.70	2.35	13.35
28	MAR	0.5	PMM	1000	0.109	0.127	0.164	-0.486	44.5	18.8	20.3	-22.3	0.020	0.033	0.058	0.275	0.981	0.976	0.953	0.906	0.88	1.78	2.68	8.82
28	MAR	0.5	POLR	1000	0.101	0.075	0.047	-0.868	41.3	11.2	5.8	-39.9	0.019	0.023	0.030	0.783	0.979	0.983	0.989	0.129	0.85	1.54	2.12	5.34
28	MAR	0.5	POLYREG	1000	0.068	0.086	0.073	-0.620	27.7	12.8	9.0	-28.5	0.022	0.031	0.046	0.428	0.955	0.955	0.957	0.566	0.85	1.54	2.26	6.71
28	MAR	0.5	RF	1000	0.008	-0.021	-0.065	-0.612	3.1	-3.1	-8.1	-28.1	0.010	0.015	0.032	0.395	0.996	0.995	0.995	0.864	0.77	1.41	2.04	8.46
28	MAR	0.5	JM	1000	0.078	0.081	0.111	-0.810	31.7	12.0	13.8	-37.2	0.019	0.025	0.045	0.681	0.984	0.985	0.972	0.482	0.95	1.72	2.68	7.06
29	MCAR	0.5	CCA	1000	0.028	0.042	0.065	0.074	11.5	6.2	8.0	3.4	0.018	0.026	0.059	0.069	0.987	0.984	0.953	0.996	0.89	1.65	2.48	14.78
29	MCAR	0.5	PMM	1000	0.050	0.023	0.108	-0.554	20.5	3.5	13.4	-25.4	0.011	0.017	0.044	0.343	0.998	0.997	0.970	0.847	0.81	1.55	2.46	8.53
29	MCAR	0.5	POLR	1000	0.050	-0.007	-0.001	-0.885	20.5	-1.0	-0.1	-40.7	0.011	0.017	0.028	0.809	0.996	0.996	0.992	0.107	0.79	1.39	2.02	5.47
29	MCAR	0.5	POLYREG	1000	-0.002	-0.008	0.012	-0.572	-0.8	-1.2	1.5	-26.3	0.018	0.023	0.046	0.364	0.975	0.981	0.961	0.702	0.77	1.39	2.11	7.23

29	MCAR	0.5	RF	1000	-0.012	-0.066	-0.034	-0.554	-4.8	-9.9	-4.3	-25.5	0.011	0.019	0.032	0.330	0.995	0.992	0.994	0.891	0.75	1.33	2.07	8.96
29	MCAR	0.5	JM	1000	0.020	-0.001	0.056	-0.820	8.0	-0.2	6.9	-37.7	0.014	0.018	0.039	0.695	0.994	1.000	0.988	0.385	0.86	1.53	2.47	6.68
30	MNAR	0.5	CCA	1000	0.041	0.050	0.108	0.075	16.6	7.4	13.4	3.5	0.020	0.023	0.036	0.028	0.977	0.984	0.975	1.000	0.90	1.57	2.19	11.11
30	MNAR	0.5	PMM	1000	0.058	0.044	0.160	-0.476	23.5	6.6	19.9	-21.9	0.010	0.015	0.041	0.248	0.997	0.995	0.977	0.816	0.79	1.46	2.21	7.09
30	MNAR	0.5	POLR	1000	0.069	0.015	0.059	-0.741	28.2	2.2	7.3	-34.1	0.012	0.013	0.019	0.567	0.993	0.997	0.997	0.090	0.79	1.33	1.83	4.95
30	MNAR	0.5	POLYREG	1000	0.017	0.012	0.059	-0.309	7.0	1.7	7.3	-14.2	0.019	0.020	0.024	0.114	0.963	0.981	0.989	0.949	0.78	1.33	1.88	7.17
30	MNAR	0.5	RF	1000	-0.002	-0.055	0.002	-0.494	-0.7	-8.1	0.2	-22.7	0.011	0.015	0.012	0.254	0.989	0.994	1.000	0.869	0.74	1.25	1.80	7.07
30	MNAR	0.5	JM	1000	0.020	0.006	0.073	-0.598	8.3	0.8	9.0	-27.5	0.014	0.016	0.022	0.369	0.994	0.996	0.995	0.595	0.87	1.46	2.15	6.50
31	MAR	0.1	CCA	5000	0.007	0.009	0.012	0.005	1.5	1.2	0.9	0.3	0.000	0.001	0.001	0.002	1.000	1.000	1.000	1.000	0.35	0.58	1.14	2.10
31	MAR	0.1	PMM	5000	0.015	0.025	0.010	-0.036	3.4	3.2	0.8	-2.3	0.001	0.001	0.001	0.003	1.000	0.999	1.000	1.000	0.36	0.60	1.20	2.24
31	MAR	0.1	POLR	5000	0.017	0.025	-0.014	-0.061	3.8	3.2	-1.1	-4.0	0.001	0.001	0.001	0.006	1.000	0.999	1.000	1.000	0.36	0.60	1.19	2.24
31	MAR	0.1	POLYREG	5000	0.004	0.014	0.021	0.009	1.0	1.8	1.7	0.6	0.000	0.001	0.002	0.003	1.000	1.000	1.000	1.000	0.36	0.59	1.21	2.23
31	MAR	0.1	RF	5000	-0.011	-0.001	-0.006	-0.030	-2.5	-0.2	-0.5	-1.9	0.001	0.001	0.001	0.003	1.000	1.000	1.000	1.000	0.35	0.59	1.19	2.20
31	MAR	0.1	JM	5000	-0.009	0.003	-0.006	-0.093	-2.1	0.4	-0.4	-6.0	0.000	0.001	0.002	0.011	1.000	1.000	1.000	1.000	0.35	0.59	1.18	2.22
32	MCAR	0.1	CCA	5000	0.000	-0.001	0.000	0.002	0.0	-0.1	0.0	0.1	0.000	0.000	0.001	0.002	1.000	1.000	1.000	1.000	0.35	0.57	1.13	2.10
32	MCAR	0.1	PMM	5000	-0.003	-0.001	-0.017	-0.045	-0.8	-0.1	-1.3	-2.9	0.000	0.000	0.001	0.004	1.000	1.000	1.000	1.000	0.35	0.58	1.14	2.12
32	MCAR	0.1	POLR	5000	0.003	0.004	-0.032	-0.065	0.6	0.5	-2.6	-4.2	0.000	0.000	0.002	0.006	1.000	1.000	1.000	1.000	0.35	0.58	1.13	2.13
32	MCAR	0.1	POLYREG	5000	-0.004	-0.003	-0.011	-0.013	-0.8	-0.4	-0.8	-0.8	0.000	0.001	0.001	0.002	1.000	1.000	1.000	1.000	0.35	0.58	1.15	2.14
32	MCAR	0.1	RF	5000	-0.005	-0.005	-0.015	-0.027	-1.1	-0.6	-1.2	-1.7	0.000	0.001	0.001	0.002	1.000	1.000	1.000	1.000	0.35	0.58	1.14	2.13
32	MCAR	0.1	JM	5000	-0.022	-0.017	-0.041	-0.098	-4.9	-2.2	-3.2	-6.3	0.001	0.001	0.003	0.011	1.000	1.000	1.000	1.000	0.34	0.57	1.12	2.08
33	MNAR	0.1	CCA	5000	0.002	0.003	0.001	0.002	0.4	0.3	0.1	0.1	0.000	0.000	0.000	0.000	1.000	1.000	1.000	1.000	0.35	0.57	1.10	2.01
33	MNAR	0.1	PMM	5000	-0.004	0.002	-0.015	-0.046	-0.9	0.3	-1.2	-3.0	0.000	0.000	0.001	0.003	1.000	1.000	1.000	1.000	0.35	0.57	1.12	2.04

33	MNAR	0.1	POLR	5000	0.003	0.007	-0.031	-0.066	0.8	0.9	-2.5	-4.2	0.000	0.000	0.001	0.005	1.000	1.000	1.000	1.000	0.35	0.57	1.11	2.05
33	MNAR	0.1	POLYREG	5000	-0.003	0.000	-0.008	-0.011	-0.7	0	-0.7	-0.7	0.000	0.000	0.001	0.001	1.000	1.000	1.000	1.000	0.35	0.57	1.12	2.07
33	MNAR	0.1	RF	5000	-0.006	-0.004	-0.015	-0.029	-1.3	-0.4	-1.2	-1.9	0.000	0.000	0.001	0.001	1.000	1.000	1.000	1.000	0.35	0.57	1.12	2.06
33	MNAR	0.1	JM	5000	-0.022	-0.016	-0.040	-0.098	-5.0	-2.0	-3.2	-6.3	0.001	0.001	0.002	0.010	1.000	1.000	1.000	1.000	0.34	0.56	1.09	2.01
34	MAR	0.2	CCA	5000	0.013	0.017	0.020	0.009	3.0	2.2	1.6	0.6	0.001	0.001	0.003	0.005	1.000	1.000	0.999	1.000	0.37	0.61	1.22	2.25
34	MAR	0.2	PMM	5000	0.027	0.046	0.011	-0.068	5.9	5.8	0.9	-4.4	0.001	0.003	0.002	0.008	1.000	0.999	1.000	0.999	0.38	0.66	1.30	2.35
34	MAR	0.2	POLR	5000	0.031	0.046	-0.033	-0.117	6.8	5.8	-2.6	-7.5	0.002	0.003	0.003	0.016	1.000	1.000	0.999	0.997	0.38	0.65	1.25	2.29
34	MAR	0.2	POLYREG	5000	0.008	0.024	0.032	0.009	1.9	3.0	2.6	0.6	0.001	0.002	0.003	0.005	1.000	1.000	0.998	1.000	0.38	0.63	1.30	2.40
34	MAR	0.2	RF	5000	-0.020	-0.003	-0.019	-0.062	-4.4	-0.4	-1.5	-4.0	0.001	0.001	0.003	0.008	1.000	1.000	1.000	0.999	0.37	0.62	1.27	2.34
34	MAR	0.2	JM	5000	-0.002	0.015	0.006	-0.155	-0.4	1.9	0.5	-10.0	0.001	0.001	0.002	0.028	1.000	1.000	1.000	0.980	0.38	0.65	1.31	2.32
35	MCAR	0.2	CCA	5000	0.001	0.001	0.001	0.003	0.1	0.2	0.1	0.2	0.001	0.001	0.002	0.004	1.000	1.000	1.000	1.000	0.37	0.61	1.21	2.24
35	MCAR	0.2	PMM	5000	-0.007	0.001	-0.033	-0.092	-1.5	0.1	-2.6	-5.9	0.001	0.001	0.003	0.012	1.000	1.000	1.000	1.000	0.37	0.61	1.20	2.18
35	MCAR	0.2	POLR	5000	0.006	0.010	-0.063	-0.125	1.3	1.3	-5.0	-8.0	0.001	0.001	0.006	0.018	1.000	1.000	0.998	0.997	0.37	0.61	1.17	2.15
35	MCAR	0.2	POLYREG	5000	-0.007	-0.003	-0.022	-0.031	-1.6	-0.4	-1.7	-2.0	0.001	0.001	0.003	0.005	1.000	1.000	1.000	1.000	0.36	0.60	1.21	2.25
35	MCAR	0.2	RF	5000	-0.010	-0.009	-0.032	-0.057	-2.2	-1.1	-2.5	-3.7	0.001	0.001	0.003	0.007	1.000	1.000	1.000	1.000	0.37	0.61	1.20	2.23
35	MCAR	0.2	JM	5000	-0.025	-0.019	-0.056	-0.169	-5.7	-2.4	-4.4	-10.8	0.001	0.002	0.005	0.032	0.998	0.999	0.996	0.956	0.36	0.61	1.19	2.14
36	MNAR	0.2	CCA	5000	0.003	0.006	0.004	0.003	0.8	0.8	0.3	0.2	0.001	0.001	0.001	0.001	1.000	1.000	1.000	1.000	0.37	0.60	1.14	2.05
36	MNAR	0.2	PMM	5000	-0.008	0.006	-0.024	-0.087	-1.7	0.7	-1.9	-5.6	0.001	0.001	0.001	0.009	1.000	1.000	1.000	1.000	0.36	0.60	1.15	2.02
36	MNAR	0.2	POLR	5000	0.007	0.015	-0.054	-0.119	1.6	1.9	-4.3	-7.7	0.001	0.001	0.004	0.015	1.000	1.000	1.000	1.000	0.36	0.60	1.11	2.00
36	MNAR	0.2	POLYREG	5000	-0.007	0.000	-0.016	-0.024	-1.5	0.0	-1.3	-1.6	0.001	0.001	0.001	0.001	1.000	1.000	1.000	1.000	0.36	0.59	1.15	2.08
36	MNAR	0.2	RF	5000	-0.011	-0.006	-0.028	-0.059	-2.5	-0.8	-2.2	-3.8	0.001	0.001	0.002	0.004	1.000	1.000	1.000	1.000	0.36	0.59	1.15	2.07
36	MNAR	0.2	JM	5000	-0.026	-0.017	-0.051	-0.152	-5.9	-2.2	-4.1	-9.8	0.001	0.001	0.004	0.024	1.000	1.000	1.000	0.999	0.36	0.59	1.13	1.99

37	MAR	0.3	CCA	5000	0.018	0.021	0.020	0.009	4.1	2.7	1.6	0.6	0.002	0.002	0.004	0.008	0.999	0.998	0.996	0.997	0.40	0.66	1.31	2.41
37	MAR	0.3	PMM	5000	0.035	0.061	0.004	-0.099	7.8	7.6	0.3	-6.4	0.002	0.005	0.003	0.014	0.997	0.990	1.000	0.995	0.41	0.71	1.37	2.40
37	MAR	0.3	POLR	5000	0.041	0.061	-0.057	-0.170	9.2	7.7	-4.5	-10.9	0.003	0.005	0.005	0.032	0.996	0.985	0.997	0.970	0.40	0.68	1.27	2.26
37	MAR	0.3	POLYREG	5000	0.012	0.030	0.032	-0.003	2.6	3.8	2.6	-0.2	0.001	0.003	0.004	0.006	0.999	0.997	0.996	1.000	0.39	0.67	1.38	2.51
37	MAR	0.3	RF	5000	-0.029	-0.009	-0.042	-0.104	-6.6	-1.1	-3.3	-6.7	0.002	0.002	0.005	0.016	0.990	0.999	0.997	0.994	0.38	0.65	1.33	2.43
37	MAR	0.3	JM	5000	0.004	0.023	0.008	-0.212	0.9	2.9	0.7	-13.6	0.001	0.002	0.004	0.049	1.000	0.999	1.000	0.894	0.41	0.70	1.43	2.36
38	MCAR	0.3	CCA	5000	0.003	0.004	0.006	0.009	0.7	0.5	0.5	0.6	0.001	0.002	0.004	0.007	1.000	0.998	0.996	0.996	0.40	0.66	1.30	2.42
38	MCAR	0.3	PMM	5000	-0.006	0.004	-0.044	-0.130	-1.4	0.6	-3.5	-8.4	0.001	0.002	0.005	0.022	1.000	1.000	0.998	0.985	0.38	0.65	1.26	2.24
38	MCAR	0.3	POLR	5000	0.010	0.017	-0.090	-0.181	2.2	2.1	-7.2	-11.6	0.001	0.002	0.011	0.036	1.000	0.999	0.969	0.933	0.39	0.65	1.19	2.16
38	MCAR	0.3	POLYREG	5000	-0.008	-0.004	-0.029	-0.046	-1.9	-0.4	-2.3	-3.0	0.001	0.002	0.004	0.008	0.999	1.000	0.998	0.996	0.38	0.63	1.26	2.35
38	MCAR	0.3	RF	5000	-0.014	-0.012	-0.045	-0.085	-3.1	-1.5	-3.6	-5.5	0.002	0.002	0.005	0.012	0.998	0.999	0.995	0.996	0.38	0.64	1.27	2.33
38	MCAR	0.3	JM	5000	-0.026	-0.020	-0.065	-0.230	-5.9	-2.5	-5.1	-14.8	0.002	0.002	0.008	0.057	0.999	0.998	0.992	0.785	0.39	0.65	1.28	2.17
39	MNAR	0.3	CCA	5000	0.009	0.010	0.006	0.006	1.9	1.3	0.5	0.4	0.001	0.002	0.002	0.002	0.999	1.000	1.000	1.000	0.40	0.63	1.19	2.11
39	MNAR	0.3	PMM	5000	-0.008	0.011	-0.031	-0.119	-1.8	1.3	-2.5	-7.7	0.001	0.001	0.002	0.016	1.000	1.000	1.000	1.000	0.38	0.63	1.17	1.98
39	MNAR	0.3	POLR	5000	0.014	0.024	-0.073	-0.160	3.1	3.0	-5.8	-10.3	0.001	0.002	0.007	0.027	1.000	0.999	0.997	0.986	0.38	0.62	1.11	1.92
39	MNAR	0.3	POLYREG	5000	-0.007	0.002	-0.024	-0.031	-1.5	0.2	-1.9	-2.0	0.001	0.002	0.002	0.002	0.999	1.000	1.000	1.000	0.37	0.61	1.17	2.07
39	MNAR	0.3	RF	5000	-0.014	-0.009	-0.042	-0.084	-3.1	-1.2	-3.3	-5.4	0.001	0.002	0.003	0.008	0.999	1.000	1.000	1.000	0.38	0.61	1.18	2.07
39	MNAR	0.3	JM	5000	-0.027	-0.018	-0.062	-0.190	-6.1	-2.3	-4.9	-12.2	0.002	0.002	0.005	0.037	0.997	1.000	1.000	0.941	0.39	0.62	1.18	1.96
40	MAR	0.4	CCA	5000	0.025	0.022	0.019	0.004	5.6	2.8	1.5	0.3	0.002	0.003	0.006	0.010	0.993	0.994	0.989	0.994	0.43	0.71	1.41	2.61
40	MAR	0.4	PMM	5000	0.041	0.072	-0.004	-0.129	9.2	9.1	-0.3	-8.3	0.003	0.008	0.004	0.023	0.990	0.960	0.997	0.975	0.43	0.77	1.45	2.44
40	MAR	0.4	POLR	5000	0.049	0.070	-0.080	-0.218	10.9	8.9	-6.4	-14.0	0.004	0.007	0.009	0.051	0.987	0.963	0.975	0.854	0.42	0.72	1.29	2.21
40	MAR	0.4	POLYREG	5000	0.015	0.031	0.029	-0.024	3.3	4.0	2.3	-1.6	0.002	0.004	0.006	0.009	0.997	0.990	0.989	0.995	0.41	0.70	1.44	2.57

40	MAR	0.4	RF	5000	-0.035	-0.018	-0.067	-0.146	-7.8	-2.3	-5.3	-9.4	0.003	0.003	0.009	0.028	0.982	0.999	0.979	0.960	0.40	0.68	1.38	2.48
40	MAR	0.4	JM	5000	0.010	0.027	0.009	-0.267	2.2	3.4	0.7	-17.2	0.002	0.003	0.005	0.077	0.999	0.997	0.998	0.777	0.45	0.76	1.56	2.41
41	MCAR	0.4	CCA	5000	0.001	0.001	0.007	0.010	0.1	0.1	0.6	0.7	0.002	0.003	0.006	0.013	0.997	0.994	0.985	0.980	0.43	0.71	1.41	2.64
41	MCAR	0.4	PMM	5000	-0.011	0.002	-0.060	-0.173	-2.5	0.3	-4.8	-11.1	0.002	0.002	0.008	0.037	0.999	0.997	0.989	0.913	0.40	0.70	1.32	2.29
41	MCAR	0.4	POLR	5000	0.010	0.017	-0.122	-0.240	2.2	2.2	-9.7	-15.4	0.001	0.003	0.018	0.062	0.999	0.995	0.878	0.707	0.40	0.67	1.21	2.12
41	MCAR	0.4	POLYREG	5000	-0.015	-0.010	-0.042	-0.070	-3.2	-1.2	-3.3	-4.5	0.002	0.003	0.007	0.015	0.995	0.997	0.985	0.980	0.39	0.66	1.31	2.42
41	MCAR	0.4	RF	5000	-0.024	-0.023	-0.065	-0.121	-5.4	-2.9	-5.2	-7.8	0.003	0.003	0.009	0.023	0.992	0.993	0.980	0.967	0.40	0.67	1.33	2.41
41	MCAR	0.4	JM	5000	-0.032	-0.027	-0.081	-0.295	-7.2	-3.4	-6.4	-19.0	0.003	0.004	0.012	0.094	0.989	0.992	0.964	0.531	0.42	0.70	1.37	2.19
42	MNAR	0.4	CCA	5000	0.008	0.012	0.004	0.008	1.8	1.5	0.3	0.5	0.002	0.003	0.002	0.002	0.998	0.999	1.000	1.000	0.43	0.68	1.25	2.17
42	MNAR	0.4	PMM	5000	-0.013	0.015	-0.038	-0.148	-2.9	1.8	-3.1	-9.5	0.002	0.002	0.003	0.024	0.998	0.999	1.000	0.972	0.39	0.67	1.20	1.93
42	MNAR	0.4	POLR	5000	0.017	0.030	-0.091	-0.190	3.7	3.8	-7.2	-12.2	0.001	0.003	0.010	0.038	1.000	0.997	0.958	0.828	0.40	0.64	1.10	1.83
42	MNAR	0.4	POLYREG	5000	-0.013	0.001	-0.036	-0.044	-2.8	0.1	-2.9	-2.8	0.002	0.002	0.004	0.004	0.991	0.998	0.999	1.000	0.39	0.62	1.17	2.05
42	MNAR	0.4	RF	5000	-0.021	-0.015	-0.059	-0.113	-4.8	-1.9	-4.7	-7.3	0.002	0.002	0.006	0.015	0.987	0.999	0.997	0.998	0.39	0.63	1.20	2.04
42	MNAR	0.4	JM	5000	-0.034	-0.023	-0.078	-0.221	-7.7	-2.9	-6.2	-14.2	0.003	0.003	0.008	0.050	0.990	0.999	0.986	0.787	0.42	0.67	1.23	1.95
43	MAR	0.5	CCA	5000	0.027	0.025	0.019	0.009	6.0	3.1	1.5	0.6	0.003	0.005	0.008	0.016	0.993	0.987	0.987	0.988	0.47	0.78	1.55	2.90
43	MAR	0.5	PMM	5000	0.046	0.086	-0.012	-0.146	10.3	10.9	-1.0	-9.4	0.004	0.010	0.005	0.029	0.992	0.944	0.998	0.951	0.46	0.85	1.54	2.53
43	MAR	0.5	POLR	5000	0.055	0.082	-0.102	-0.258	12.3	10.3	-8.1	-16.6	0.004	0.009	0.014	0.071	0.984	0.913	0.938	0.680	0.44	0.75	1.29	2.15
43	MAR	0.5	POLYREG	5000	0.014	0.033	0.024	-0.049	3.1	4.2	1.9	-3.1	0.003	0.005	0.007	0.014	0.994	0.980	0.993	0.980	0.43	0.72	1.49	2.60
43	MAR	0.5	RF	5000	-0.047	-0.028	-0.098	-0.191	-10.6	-3.6	-7.8	-12.3	0.005	0.004	0.015	0.045	0.944	0.991	0.938	0.906	0.41	0.71	1.43	2.53
43	MAR	0.5	JM	5000	0.010	0.032	0.008	-0.315	2.3	4.1	0.7	-20.3	0.002	0.005	0.006	0.106	0.998	0.994	1.000	0.669	0.49	0.84	1.73	2.45
44	MCAR	0.5	CCA	5000	0.004	0.008	0.012	0.017	0.9	1.1	1.0	1.1	0.003	0.005	0.009	0.017	0.994	0.986	0.972	0.982	0.47	0.79	1.56	2.93
44	MCAR	0.5	PMM	5000	-0.009	0.012	-0.071	-0.208	-2.0	1.5	-5.6	-13.4	0.002	0.004	0.011	0.052	0.997	0.994	0.967	0.806	0.43	0.76	1.41	2.33

44	MCAR	0.5	POLR	5000	0.015	0.027	-0.148	-0.290	3.3	3.4	-11.7	-18.6	0.002	0.004	0.027	0.090	0.998	0.983	0.762	0.500	0.42	0.71	1.22	2.07
44	MCAR	0.5	POLYREG	5000	-0.015	-0.005	-0.051	-0.092	-3.3	-0.6	-4.0	-5.9	0.003	0.004	0.010	0.022	0.986	0.982	0.951	0.952	0.41	0.69	1.36	2.47
44	MCAR	0.5	RF	5000	-0.030	-0.024	-0.083	-0.155	-6.7	-3.1	-6.6	-10.0	0.004	0.004	0.013	0.033	0.975	0.987	0.945	0.928	0.42	0.71	1.40	2.50
44	MCAR	0.5	JM	5000	-0.033	-0.023	-0.089	-0.348	-7.5	-2.9	-7.1	-22.4	0.004	0.005	0.015	0.129	0.989	0.990	0.954	0.410	0.46	0.77	1.50	2.23
45	MNAR	0.5	CCA	5000	0.014	0.018	0.012	0.014	3.0	2.3	0.9	0.9	0.003	0.004	0.004	0.004	0.991	0.995	0.996	1.000	0.48	0.75	1.35	2.28
45	MNAR	0.5	PMM	5000	-0.013	0.024	-0.035	-0.164	-3.0	3.0	-2.8	-10.6	0.002	0.003	0.004	0.030	0.994	0.995	0.998	0.895	0.41	0.72	1.26	1.91
45	MNAR	0.5	POLR	5000	0.024	0.040	-0.098	-0.208	5.4	5.0	-7.8	-13.4	0.002	0.004	0.012	0.046	0.998	0.978	0.911	0.635	0.41	0.67	1.09	1.74
45	MNAR	0.5	POLYREG	5000	-0.012	0.005	-0.039	-0.050	-2.6	0.6	-3.1	-3.2	0.003	0.003	0.005	0.006	0.978	0.984	0.986	0.999	0.40	0.64	1.17	2.01
45	MNAR	0.5	RF	5000	-0.023	-0.017	-0.069	-0.137	-5.2	-2.2	-5.5	-8.8	0.003	0.003	0.008	0.021	0.978	0.994	0.980	0.976	0.41	0.66	1.23	2.02
45	MNAR	0.5	JM	5000	-0.035	-0.022	-0.084	-0.237	-7.9	-2.8	-6.7	-15.2	0.004	0.004	0.011	0.059	0.983	0.997	0.977	0.658	0.46	0.72	1.31	1.96

Abbreviations: CI: confidence interval; MCAR: missing completely at random; MAR: missing at random; MNAR: missing not at random; CCA: complete case analysis; PMM: predictive mean matching; POLR: proportional odds logistic regression; POLYREG: polytomous regression model; RF: random forest; JM: joint model; NA: not available; Inf: infinity

Legend: cells highlighted in this color: percent bias >10%; cells highlighted in this color: no values available
