# Supplementary material for: Multiple imputation for missing values in ordinal variables from cancer registry data when performing Cox proportional hazards regression
Source: BMC Med Res Methodol. 2026 Feb 6;26:47. doi: 10.1186/s12874-026-02790-8 (PMC12930733; doi:10.1186/s12874-026-02790-8)
Supplement: Supplementary file 2 — Additional file 2. R Code for the simulation. [file 12874_2026_2790_MOESM2_ESM.pdf]

```r
#-----------------------------------------------------------------------------#
# Simulation Set Up ---------------------------------------------------
#-----------------------------------------------------------------------------#

# 3 missingness mechanisms: MCAR, MAR, MNAR
# 5 missingness proportions: 10%, 20%, 30%, 40%, 50%
# 3 sample sizes: 500, 1000, 5000

# 5 different MI procedures:
# - Predictive Mean Matching (pmm)
# - Proportional Odds Model (polr)
# - Multinominal Regression (polyreg)
# - Random Forest (rf)
# - Joint Model (jm)

# + Complete Case Analysis

# For one nsim n=45 data generation scenarios: 3*5*3=45
# for each data generation scenarios all methods are applied (45*6=270)

# Separate loops for each sample size scenario -> saves some time per loop

#-----------------------------------------------------------------------------#
# Load Packages ------------------------------------------------------------
#-----------------------------------------------------------------------------#

library(dplyr)      # for data manipulation
library(mice)       # for imputation and amputation
library(survival)   # for Cox proportional hazards model
library(MASS)       # for imputation
library(jomo)       # for imputation
library(mitml)      # for pooling JM
library(parallel)   # for parallel computation

#-----------------------------------------------------------------------------#
# Set Nsim -------------------------------------------------------------------
#-----------------------------------------------------------------------------#

nsim <- 1000

#-----------------------------------------------------------------------------#
# Generate Grid for MI Results ---------------------------------------
#-----------------------------------------------------------------------------#

grid=expand.grid(
  sim = 1:nsim,
  missmech = c("MCAR", "MAR", "MNAR"),
  missprop = seq(0.1,0.5,0.1),
  MImethod = c("cca", "pmm", "polr", "polyreg", "rf", "jm"),
  nobs = c(500) # CAVE! Change the number according to the sample size!
)

grid$scenario <- as.numeric(interaction(grid$missmech, grid$missprop, grid$sim))
```

```r
# Include the number of scenarios
nscen <- nrow(grid)/nsim

# Include a number for the simulation replication + vector for results
grid$rep = sort(c(rep(1:nscen,nsim)))

# Include columns for all results
grid$true.HR.ECOG4 <- grid$true.HR.ECOG3 <- grid$true.HR.ECOG2 <- grid$true.HR.ECOG1 <- NA
grid$Up_95CI.ECOG1 <- grid$Low_95CI.ECOG1 <- grid$HR.ECOG1 <- NA
grid$Up_95CI.ECOG2 <- grid$Low_95CI.ECOG2 <- grid$HR.ECOG2 <- NA
grid$Up_95CI.ECOG3 <- grid$Low_95CI.ECOG3 <- grid$HR.ECOG3 <- NA
grid$Up_95CI.ECOG4 <- grid$Low_95CI.ECOG4 <- grid$HR.ECOG4 <- NA

# Include columns for diagnostic evaluation
grid$Median.obs <- grid$Mean.obs <- grid$N.obs <- NA
grid$Max.obs <- grid$Min.obs <- grid$SD.obs <- NA
grid$Median.imp <- grid$Mean.imp <- grid$N.imp <- NA
grid$Max.imp <- grid$Min.imp <- grid$SD.imp <- NA

grid$missmech <- as.character(grid$missmech)
grid$MImethod <- as.character(grid$MImethod)

# Columns to be converted to numeric
ColNum <- c("true.HR.ECOG4", "true.HR.ECOG3", "true.HR.ECOG2", "true.HR.ECOG1",
        "Up_95CI.ECOG1", "Low_95CI.ECOG1", "HR.ECOG1",
        "Up_95CI.ECOG2", "Low_95CI.ECOG2", "HR.ECOG2",
        "Up_95CI.ECOG3", "Low_95CI.ECOG3", "HR.ECOG3",
        "Up_95CI.ECOG4", "Low_95CI.ECOG4", "HR.ECOG4",
        "Median.obs", "Mean.obs", "N.obs", "Max.obs",
        "Min.obs", "SD.obs", "Median.imp", "Mean.imp",
        "N.imp", "Max.imp", "Min.imp", "SD.imp")

# Apply the conversion to the selected columns
grid[ColNum] <- lapply(grid[ColNum], as.numeric)

#----------------------------------------------------------------------------#
# True Values Complete Data Set Analysis ------------------------
#----------------------------------------------------------------------------#

# Factor Variables
ColFac <- c("Sex", "DiagnosisYear", "C34.0", "C34.1", "C34.3",
        "AdenoCa", "SquamousCa", "SCLC", "TherIntent", "RadioTher",
        "SurResStatus", "SystemTher")

# Apply the conversion to the selected columns
data[ColFac] <- lapply(data[ColFac], as.factor)

# Define right levels for ECOG variable
data$ECOG <- as.numeric(data$ECOG)-1
data$ECOG <- as.factor(data$ECOG)

# Ordinal Variables
```

```
data$Grading <- factor(data$Grading, order = TRUE)
data$UICC_Stage <- factor(data$UICC_Stage, order = TRUE)

# Cox Proportional Hazards Model
TrueValue <- coxph(Surv(SurvivalTime, Event) ~ Sex + Age + DiagnosisYear + ECOG
                   + C34.0 + C34.1 + C34.3 + Grading + AdenoCa + SquamousCa
                   + SCLC + UICC_Stage + TherIntent + RadioTher + SurResStatus
                   + SystemTher, data=data)

summary_cox <- summary(TrueValue)

# Extract the ECOG-PS results of the Cox Survival Analysis
ECOGres <- summary_cox$conf.int[grep("ECOG", rownames(summary_cox$conf.int)), ]
ECOGrestrue <- data.frame(
  ID = c(1, 1, 1, 1),
  ECOG = c("ECOG1", "ECOG2", "ECOG3", "ECOG4"),
  HR = ECOGres[, "exp(coef)"]
)

# Reshape the results for ECOG-PS into a wide format
ECOGrestrue.wide <- reshape(data = ECOGrestrue,
                       idvar = "ID",
                       v.names = c("HR"),
                       timevar = "ECOG",
                       direction = "wide")

# Save the HR of the Cox Survival Analysis into the grid
grid$true.HR.ECOG4 <- ECOGrestrue.wide$HR.ECOG4
grid$true.HR.ECOG3 <- ECOGrestrue.wide$HR.ECOG3
grid$true.HR.ECOG2 <- ECOGrestrue.wide$HR.ECOG2
grid$true.HR.ECOG1 <- ECOGrestrue.wide$HR.ECOG1

#--------------------------------------------------------------------------#
# Function for Generating Missing ECOG-Values  ----------------
#--------------------------------------------------------------------------#

F_generate_missing_data <- function(data, missmech, missprop, myfreq) {

  # Columns to be converted to numeric
  ColNum <- c("Sex", "DiagnosisYear", "C34.0", "C34.1", "C34.3",
          "Grading", "AdenoCa", "SquamousCa", "SCLC", "UICC_Stage",
          "TherIntent", "RadioTher", "SurResStatus", "SystemTher")

  # Apply the conversion to the selected columns
  data[ColNum] <- lapply(data[ColNum], as.numeric)
  data$ECOG <- as.numeric(data$ECOG)-1

  # Define Missingness Pattern -> univariate pattern only for ECOG-PS
  pattern <- matrix(c(1, 1, 1, 1, 1, 1, 0, 1, 1, 1, 1, 1, 1, 1, 1, 1, 1, 1, 1),
              nrow = 1, ncol = 19, byrow = TRUE)

  # Define Weights & perform 'ampute' procedure
  if (missmech == "MCAR") {
```

```
    weight <- matrix(c(0, 0, 0, 0, 0, 0, 0, 0, 0, 0, 0, 0, 0, 0, 0, 0, 0, 0, 0),
              nrow = 1, ncol = 19, byrow = TRUE)

    # Generate missing ECOG-PS values
    res <- ampute(data, freq = myfreq, prop = missprop, patterns = pattern, weights = weight)

  } else if (missmech == "MAR") {
    weight <- matrix(c( 0, ECOGMiss_B$Age, ECOGMiss_B$DiagnosisYear, 0, 0,
                ECOGMiss_B$NelsonAalenEst, 0, 0, 0,
                ECOGMiss_B$C34.31, ECOGMiss_B$Grading, 0, 0, 0,
                ECOGMiss_B$UICC_Stage, ECOGMiss_B$TherIntent,
                ECOGMiss_B$RadioTher2, 0, ECOGMiss_B$SystemTher),
                nrow = 1, ncol = 19, byrow = TRUE)

    # Generate missing ECOG-PS values
    res <- ampute(data, freq = myfreq, prop = missprop, patterns = pattern, weights = weight)

    } else if (missmech == "MNAR") {
    weight <- matrix(c(0, 0, 0, 0, 0, 0, 1, 0, 0, 0, 0, 0, 0, 0, 0, 0, 0, 0, 0),
              nrow = 1, ncol = 19, byrow = TRUE)

    # Generate missing ECOG-PS values
    res <- ampute(data, freq = myfreq, prop = missprop, patterns = pattern, weights = weight, type =
"MID")

  }

  # Return Data Sets with Missing Values
  return(res$amp)
}

#-----------------------------------------------------------------------------#
# Function for MI and Cox Regression ------------------------------
#-----------------------------------------------------------------------------#

F_perform_mi_and_cox <- function(data, missprop, m, method) {

  # Create indicator variable for missing ECOG-PS values
  data$ECOG_Miss.Ind <- ifelse(is.na(data$ECOG), 1, 0)

  # Mean, median, SD, min, max for the observed ECOG values without imputation
  observed_stats <- data.frame(
    N = sum(data$ECOG_Miss.Ind == 0),
    Mean = mean(data$ECOG, na.rm = TRUE),
    Median = median(data$ECOG, na.rm = TRUE),
    SD = sd(data$ECOG, na.rm = TRUE),
    Min = min(data$ECOG, na.rm = TRUE),
    Max = max(data$ECOG, na.rm = TRUE)
  )

  #Remove ECOG-PS missingness indicator variable from data for MI
  data <- subset(data, select = -ECOG_Miss.Ind)
```

```
# Define factor variables
ColFac <- c("Sex", "DiagnosisYear", "ECOG", "C34.0", "C34.1", "C34.3",
          "AdenoCa", "SquamousCa", "SCLC", "TherIntent",
          "RadioTher", "SurResStatus", "SystemTher")
data[ColFac] <- lapply(data[ColFac], as.factor)

# Define ordinal variables for Cox Regression
data$Grading <- factor(data$Grading, order = TRUE)
data$UICC_Stage <- factor(data$UICC_Stage, order = TRUE)

if (method == "cca") {

  # Perform Cox Proportional Hazards Model
  CCA <- coxph(Surv(SurvivalTime, Event) ~ Sex + Age + DiagnosisYear + ECOG
           + C34.0 + C34.1 + C34.3 + Grading + AdenoCa + SquamousCa
           + SCLC + UICC_Stage + TherIntent + RadioTher + SurResStatus
           + SystemTher, data=data)

  summary_cox <- summary(CCA)

  # Store Results in Data Frame
  ECOGres <- summary_cox$conf.int[grep("ECOG", rownames(summary_cox$conf.int)), ]
  ECOGresdf <- data.frame(
    ID = c(1, 1, 1, 1),
    ECOG = c("ECOG1", "ECOG2", "ECOG3", "ECOG4"),
    HR = ECOGres[, "exp(coef)"],
    Low_95CI = ECOGres[, "lower .95"],
    Up_95CI = ECOGres[, "upper .95"]
  )

  # Create indicator variable for missing ECOG-PS values
  data$ECOG_Miss.Ind <- ifelse(is.na(data$ECOG), 1, 0)

  # Give NAs for imputed values
  pooled_stats <- data.frame(
    N = sum(data$ECOG_Miss.Ind == 1),
    Mean = NA,
    Median = NA,
    SD = NA,
    Min = NA,
    Max = NA
  )

  #Remove ECOG-PS indicator variable from data
  data <- subset(data, select = -ECOG_Miss.Ind)

} else if (method %in% c("pmm", "polr", "polyreg", "rf")){

  # Removal of variable "SurvivalTime" from data for MI
  SurvTime <- data[, "SurvivalTime"]
  data <- data[, !(names(data) %in% "SurvivalTime")]

  # Define ordinal variables
```

```r
data$ECOG <- factor(data$ECOG, order = TRUE)

# Perform Multiple Imputation
MIsurv <- mice(data, m = m, method = method)

# Add the "SurvivalTime" variable
MIsurvLong <- complete(MIsurv, action = "long", include = TRUE)
MIsurvLong$SurvivalTime <- rep(SurvTime, times = m+1)

# Create indicator variable for ECOG missing
data$ECOG_Miss.Ind <- ifelse(is.na(data$ECOG), 1, 0)

# Replicate the variable m times on the imputed dataset
MIsurvLong$ECOG_Miss.Ind <- rep(data$ECOG_Miss.Ind, times = m+1)

# Define ECOG as numeric variable for diagnostic evaluation
MIsurvLong$ECOG <- as.numeric(MIsurvLong$ECOG)-1

# Pooled mean, median, SD, min, max for the imputed ECOG values
# Using function pool.scalar from MICE
MEAN <- sapply(1:m, function(i) mean(MIsurvLong$ECOG[MIsurvLong$.imp == i &
MIsurvLong$ECOG_Miss.Ind == 1]))
VAR.MEAN <- sapply(1:m, function(i) var(MIsurvLong$ECOG[MIsurvLong$.imp == i &
MIsurvLong$ECOG_Miss.Ind == 1]))
POOLED <- pool.scalar(Q = MEAN, U= VAR.MEAN, n = sum(data$ECOG_Miss.Ind == 1))

# Can't use Rubin's Rules to pool Median
# Therefore take the median from the medians
MEDIAN <- sapply(1:m, function(i) {
  median(MIsurvLong$ECOG[MIsurvLong$.imp == i & MIsurvLong$ECOG_Miss.Ind == 1])})

pooled_stats <- data.frame(
  N = sum(data$ECOG_Miss.Ind == 1),
  Mean = POOLED$qbar,
  Median = median(MEDIAN),
  SD = sqrt(POOLED$t),
  Min = min(MIsurvLong$ECOG[MIsurvLong$ECOG_Miss.Ind == 1], na.rm = TRUE),
  Max = max(MIsurvLong$ECOG[MIsurvLong$ECOG_Miss.Ind == 1], na.rm = TRUE)
)

#Remove ECOG-PS indicator variable from data
data <- subset(data, select = -ECOG_Miss.Ind)

# Recode ECOG-PS as factor variable for HR estimates for each ECOG category
MIsurvLong$ECOG <- factor(MIsurvLong$ECOG, order=FALSE)
MIsurv <- as.mids(MIsurvLong)

# Fit the Cox proportional hazards model
MIpool <- with(MIsurv, coxph(Surv(SurvivalTime, Event) ~ Sex + Age + DiagnosisYear + ECOG
                 + C34.0 + C34.1 + C34.3 + Grading + AdenoCa + SquamousCa
                 + SCLC + UICC_Stage + TherIntent + RadioTher + SurResStatus
                 + SystemTher))
```

```
    # Pool the results with Rubin's rules and summarize with confidence intervals
    summary_cox <- summary(pool(MIpool), conf.int = TRUE, exponentiate = TRUE)

    # Extract the results for the ECOG-PS scores
    ecog_res <- summary_cox[grep("ECOG", summary_cox$term), ]
    ECOGresdf <- data.frame(
      ID = c(1, 1, 1, 1),
      ECOG = c("ECOG1", "ECOG2", "ECOG3", "ECOG4"),
      HR = ecog_res$estimate,
      Low_95CI = ecog_res$`2.5 %`,
      Up_95CI = ecog_res$`97.5 %`
    )

  } else if (method == "jm") {

    # Define ordinal variables for imputation
    data$ECOG <- factor(data$ECOG, order = TRUE)

    # Define all the inputs:
    nburn=as.integer(500)
    nbetween=as.integer(500)
    nimp=as.integer(m)

    # Removal of variable "SurvivalTime" from data for MI
    SurvTime <- data[, "SurvivalTime"]

    # Define the outcome variable (Y) and predictors (X)
    Y <- data[, "ECOG"]   # Outcome variable with missing values

    # All other predictor variables except ECOG and SurvivalTime
    X <- data[, !(names(data) %in% c("ECOG", "SurvivalTime"))]

    # Run the Joint Model (JM) imputation
    MIsurvJM <- jomo1(Y = as.data.frame(Y), X = X, nburn = nburn, nbetween = nbetween, nimp = 
nimp)

    # Rename Y variable to ECOG
    colnames(MIsurvJM)[colnames(MIsurvJM) == "Y"] <- "ECOG"
    MIsurvJM$SurvivalTime <- rep(SurvTime, times = nimp + 1)

    # Create indicator variable for missing ECOG-PS values
    data$ECOG_Miss.Ind <- ifelse(is.na(data$ECOG), 1, 0)

    # Replicate the variable m times on the imputed dataset
    MIsurvJM$ECOG_Miss.Ind <- rep(data$ECOG_Miss.Ind, times = nimp + 1)

    # Define ECOG-PS as numeric variable
    MIsurvJM$ECOG <- as.numeric(MIsurvJM$ECOG)-1

    # Pooled mean, median, SD, min, max for the imputed ECOG-PS values
    # Using function pool.scalar from MICE
    MEAN <- sapply(1:m, function(i) mean(MIsurvJM$ECOG[MIsurvJM$Imputation == i & 
MIsurvJM$ECOG_Miss.Ind == 1]))
```

```
  VAR.MEAN <- sapply(1:m, function(i) var(MIsurvJM$ECOG[MIsurvJM$Imputation == i &
MIsurvJM$ECOG_Miss.Ind == 1]))
  POOLED <- pool.scalar(Q = MEAN, U= VAR.MEAN, n = sum(data$ECOG_Miss.Ind == 1))

   MEDIAN <- sapply(1:m, function(i) {
    median(MIsurvJM$ECOG[MIsurvJM$Imputation == i & MIsurvJM$ECOG_Miss.Ind == 1])})

  pooled_stats <- data.frame(
    N = sum(data$ECOG_Miss.Ind == 1),
    Mean = POOLED$qbar,
    Median = median(MEDIAN),
    SD = sqrt(POOLED$t),
    Min <- min(MIsurvJM$ECOG[MIsurvJM$ECOG_Miss.Ind == 1 & MIsurvJM$Imputation >= 1]),
    Max <- max(MIsurvJM$ECOG[MIsurvJM$ECOG_Miss.Ind == 1 & MIsurvJM$Imputation >= 1])
  )

  # Define ECOG-PS as factor variable for HR estimates for each ECOG-PS category
  MIsurvJM$ECOG <- factor(MIsurvJM$ECOG, order=FALSE)

  # Remove ECOG-PS indicator variable from data set
  data <- subset(data, select = -ECOG_Miss.Ind)

  # Transform the long Format into a List Format
  MIsurv <-jomo2mitml.list(MIsurvJM)

  # Fit the Cox proportional hazards model
  MIpool <- with(MIsurv, coxph(Surv(SurvivalTime, Event) ~ Sex + Age + DiagnosisYear + ECOG
                      + C34.0 + C34.1 + C34.3 + Grading + AdenoCa + SquamousCa
                      + SCLC + UICC_Stage + TherIntent + RadioTher + SurResStatus
                      + SystemTher))

  # Pool the results with Rubin's rules and summarize with confidence intervals
  summary_cox <- summary(pool(MIpool), conf.int = TRUE, exponentiate = TRUE)

  # Extract the results for the ECOG-PS scores
  ecog_res <- summary_cox[grep("ECOG", summary_cox$term), ]
  ECOGresdf <- data.frame(
    ID = c(1, 1, 1, 1),
    ECOG = c("ECOG1", "ECOG2", "ECOG3", "ECOG4"),
    HR = ecog_res$estimate,
    Low_95CI = ecog_res$`2.5 %`,
    Up_95CI = ecog_res$`97.5 %`
  )

}

# Reshape the results for ECOG-PS into a wide format
ecog_results.wide <- reshape(data = ECOGresdf,
                     idvar = "ID",
                     v.names = c("HR", "Low_95CI", "Up_95CI"),
                     timevar = "ECOG",
                     direction = "wide")
```

```
  # Return the hazard ratios and confidence intervals for ECOG1 to ECOG4
  return(c(HR.ECOG1 = ecog_results.wide$HR.ECOG1, Low_95CI.ECOG1 =
ecog_results.wide$Low_95CI.ECOG1, Up_95CI.ECOG1 = ecog_results.wide$Up_95CI.ECOG1,
        HR.ECOG2 = ecog_results.wide$HR.ECOG2, Low_95CI.ECOG2 =
ecog_results.wide$Low_95CI.ECOG2, Up_95CI.ECOG2 = ecog_results.wide$Up_95CI.ECOG2,
        HR.ECOG3 = ecog_results.wide$HR.ECOG3, Low_95CI.ECOG3 =
ecog_results.wide$Low_95CI.ECOG3, Up_95CI.ECOG3 = ecog_results.wide$Up_95CI.ECOG3,
        HR.ECOG4 = ecog_results.wide$HR.ECOG4, Low_95CI.ECOG4 =
ecog_results.wide$Low_95CI.ECOG4, Up_95CI.ECOG4 = ecog_results.wide$Up_95CI.ECOG4,
        N.obs = observed_stats$N, Mean.obs = observed_stats$Mean, Median.obs =
observed_stats$Median, SD.obs = observed_stats$SD,
        Min.obs = observed_stats$Min, Max.obs = observed_stats$Max,
        N.imp = pooled_stats$N, Mean.imp = pooled_stats$Mean, Median.imp =
pooled_stats$Median, SD.imp = pooled_stats$SD,
        Min.imp = pooled_stats$Min, Max.imp = pooled_stats$Max))
}

#----------------------------------------------------------------------------#
# Function for Parallel Computation --------------------------------
#----------------------------------------------------------------------------#

F_SIMClusterScenario <- function(scenario) {

  # Generate the data frame with missing ECOG-PS values
  df <- F_generate_missing_data(
    data = data,
    missmech = grid$missmech[grid$scenario == scenario][1],
    missprop = grid$missprop[grid$scenario == scenario][1],
    myfreq = c(1.0)
  )

  # Create list to save results for each scenario & replication
  scenario_results <- list()

  # Run the MI and perform Cox Regression for each data set
  for (MImethod in unique(grid$MImethod)) {

    results <- F_perform_mi_and_cox(
      df,
      missprop = grid$missprop[grid$scenario == scenario][1],
      m = (grid$missprop[grid$scenario == scenario][1] * 100),
      method = MImethod
    )

    # Identify the right line in the grid with the specific scenario & MImethod
    grid_index <- which(grid$scenario == scenario & grid$MImethod == MImethod)

    # Save the results
    scenario_results[[as.character(grid_index)]] <- results
  }

  # return the results
  return(scenario_results)
```

```
}

#---------------------------------------------------------------------------#
# Setup for Parallel Computation -----------------------------------
#---------------------------------------------------------------------------#

# Detect number of cores
numCores <- detectCores() - 1

# Set up the cluster
cl <- makeCluster(numCores)

# +++ Set seed ++++
clusterSetRNGStream(cl, 10102024)

# Load R packages on each of the cluster
clusterEvalQ(cl, {
  library(mice)
  library(survival)
  library(MASS)
  library(jomo)
  library(mitml)
})

# Export the objects and functions required for the cluster
clusterExport(cl, varlist = c("grid", "data","ECOGMiss_B","F_generate_missing_data",
"F_perform_mi_and_cox"))

#---------------------------------------------------------------------------#
# Parallel Computation ---------------------------------------------
#---------------------------------------------------------------------------#

# Measure Time

start.time.1 <- Sys.time()

results_list <- parLapply(cl, unique(grid$scenario), F_SIMClusterScenario)

for (res in results_list) {
  for (grid_index in names(res)) {
    grid[as.numeric(grid_index), 12:35] <- res[[grid_index]]
  }
}

(time.1 <- Sys.time() - start.time.1)

stopCluster(cl)
```
