# Supplementary material for: Multiple imputation for missing values in ordinal variables from cancer registry data when performing Cox proportional hazards regression
Source: BMC Med Res Methodol. 2026 Feb 6;26:47. doi: 10.1186/s12874-026-02790-8 (PMC12930733; doi:10.1186/s12874-026-02790-8)
Supplement: Supplementary file 3 — Additional file 3. Simulation scenarios for N=3,000 with MAR missingness. [file 12874_2026_2790_MOESM3_ESM.pdf]

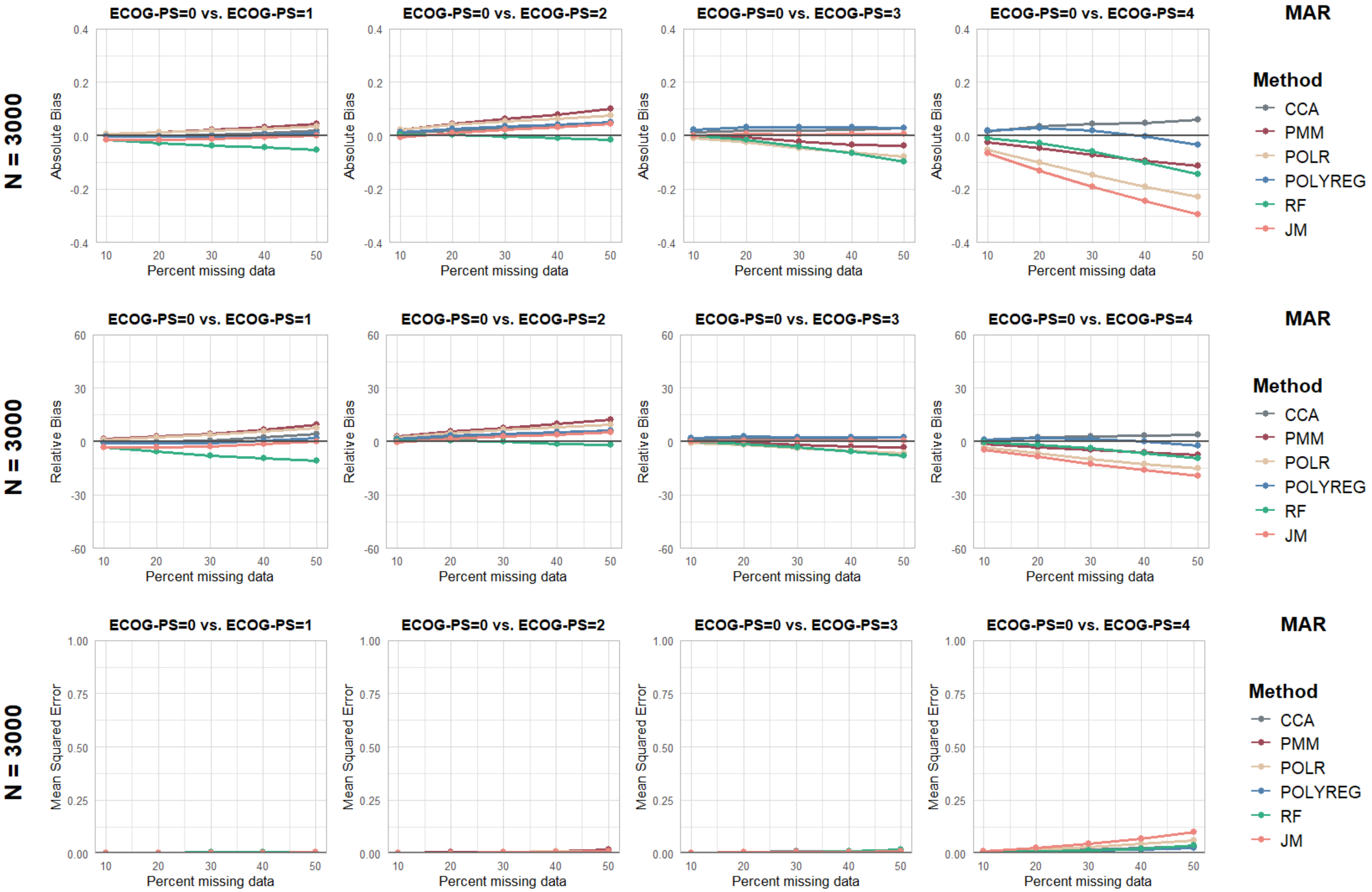
N = 3000
ECOG-PS=0 vs. ECOG-PS=1
ECOG-PS=0 vs. ECOG-PS=2
ECOG-PS=0 vs. ECOG-PS=3
ECOG-PS=0 vs. ECOG-PS=4
Absolute Bias
Relative Bias
Mean Squared Error
Percent missing data
MAR
Method
CCA
PMM
POLR
POLYREG
RF
JM

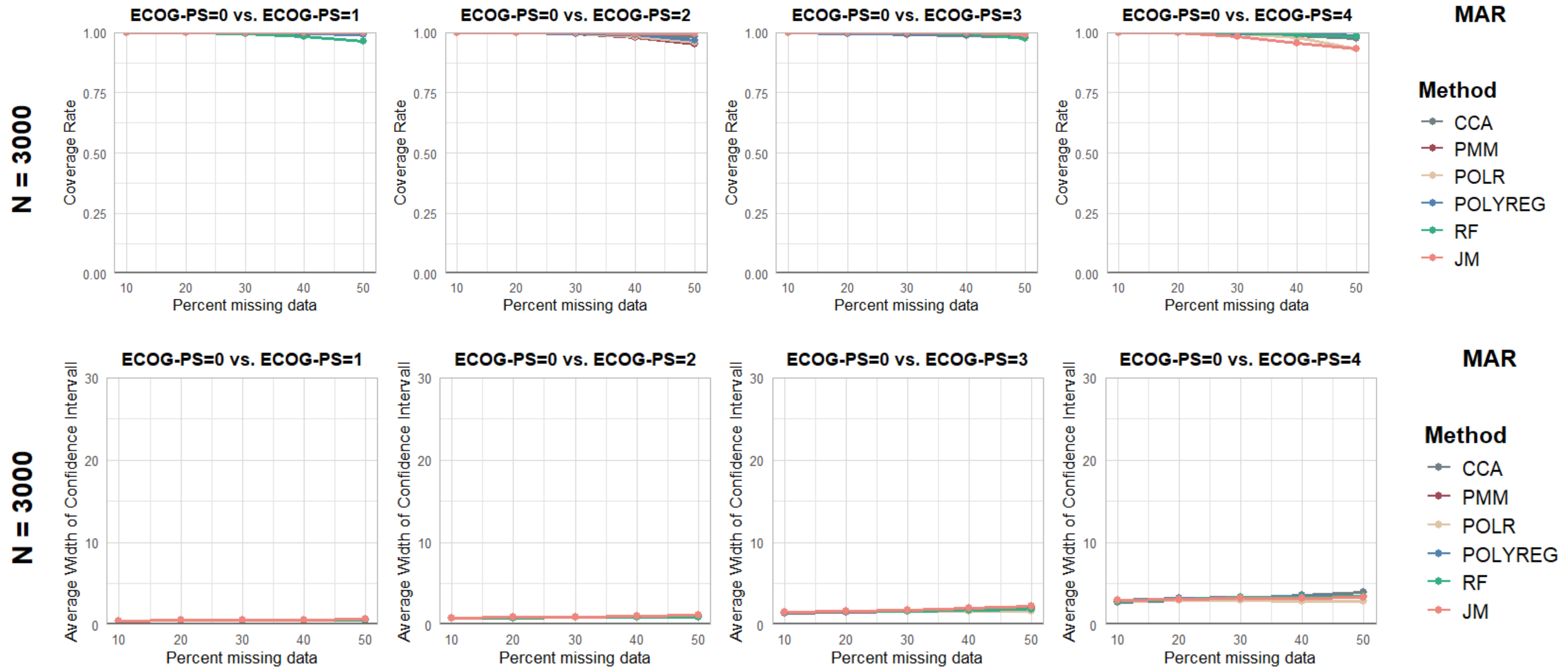

N = 3000
ECOG-PS=0 vs. ECOG-PS=1
ECOG-PS=0 vs. ECOG-PS=2
ECOG-PS=0 vs. ECOG-PS=3
ECOG-PS=0 vs. ECOG-PS=4
MAR
Coverage Rate
1.00
0.75
0.50
0.25
0.00
10
20
30
40
50
Percent missing data
Method
CCA
PMM
POLR
POLYREG
RF
JM
N = 3000
ECOG-PS=0 vs. ECOG-PS=1
ECOG-PS=0 vs. ECOG-PS=2
ECOG-PS=0 vs. ECOG-PS=3
ECOG-PS=0 vs. ECOG-PS=4
MAR
Average Width of Confidence Interval
30
20
10
0
10
20
30
40
50
Percent missing data
Method
CCA
PMM
POLR
POLYREG
RF
JM
